# Adaptive dynamic crayfish algorithm with multi-enhanced strategy for global high-dimensional optimization and real-engineering problems

**DOI:** 10.1038/s41598-024-81144-0

**Published:** 2025-03-27

**Authors:** Mohamed Elhosseny, Mahmoud Abdel-Salam, Ibrahim M. El-Hasnony

**Affiliations:** 1 College of Computing and Informatics, University of Sharjah, Sharjah, UAE; 2 Faculty of Computers and Information Science, Mansoura University, Mansoura, Egypt

**Keywords:** Crayfish, Engineering problems, Adaptive, Local escape operator, Inertia weight, Engineering, Mathematics and computing

## Abstract

The Crayfish Optimization Algorithm (COA) is a recent powerful algorithm that is sometimes plagued by poor convergence speed and a tendency to rapidly converge to the local optimum. This study introduces a variation of the COA called Adaptive Dynamic COA with a Locally enhanced escape operator (AD-COA-L) to tackle these issues. Firstly, the algorithm utilizes the Bernoulli map initialization strategy to quickly establish a high-quality population that is evenly distributed. This helps the algorithm to promptly reach the proper search area. Additionally, in order to mitigate the likelihood of getting trapped in local optima and improve the quality of the obtained solution, an Adaptive Lens Opposition-Based Learning (ALOBL) mechanism is applied. Moreover, the local escape operator (LEO) is utilized to aggressively discourage the adoption of isolated solutions and encourage the sharing of information within the search area. Finally, a new inertia weight is suggested to improve the search capability of COA and prevent it from being stuck in local optima by enhancing the exploitation capability of COA. AD-COA-L is evaluated against eight advanced state-of-the-art variations and ten classical and recent metaheuristic algorithms on 29 benchmark functions from CEC2017 of varying dimensions (50 and 100). AD-COA-L demonstrates superior accuracy, balanced exploration-exploitation and convergence speed, compared to other algorithms across most benchmark functions. Furthermore, we evaluated the proficiency of AD-COA-L in tackling seven demanding real-world and restricted engineering optimization challenges. The experimental findings clearly illustrate the competitiveness and advantages of the proposed AD-COA-L algorithm.

## Introduction

With the development of Artificial Intelligence (AI), optimization has become a crucial mathematical methodology to find an optimum solution among complex problems in all walks of life. Optimization has gained wide applications in fault diagnosis^1^, service composition^2^, the agriculture field^3^, path planning^4^, image segmentation^5^, intrusion detection^6,7^, feature selection^8,9^, and parameter identification of photovoltaic models^10^. Most of these optimization tasks involve high complexity due to large-scale dimensionality, non-linearity, and non-convexity, which are computationally challenging^11^. It is well known that single-objective optimization, dealing with the optimal solution for a single performance criterion, often faces complex landscapes. In contrast to multi-objective optimization, where a set of non-dominated solutions represented by a Pareto front is produced, the present study deals exclusively with single-objective optimization^12^. The challenge is increased due to the scale and complexity of the problem at hand.

Traditional methods for solving optimization problems include classical techniques such as linear programming^13^, Newton’s method^14^, and conjugate gradient methods^15^. While these methods might work quite well for small or relatively simple problems, they often tend to break down when real-world applications involving thousands of variables and a multitude of constraints are considered. These traditional methods are highly time-consuming and also tend to converge prematurely to local optima, especially in non-convex problem spaces^16^. Therefore, in more realistic high-dimensional optimization settings, these methods usually cannot produce a solution that would be satisfactory.

Therefore, in the last few decades, many researchers have been developing Metaheuristic Algorithms (MAs) in order to surmount the limitations of the classical methods, by allowing greater flexibility and robustness while handling complex optimization problems^17^. MAs represent a class of stochastic optimization techniques that do not use gradient information for the optimization process; hence, it is also suitable for solving nonlinear, nonconvex, high-dimensional problems. Unlike in the case of classical methods, these metaheuristics are superior to them because they can handle complex search spaces by effectively combining global and local search strategies that enable them to avoid local optima and reach near-optimal solutions with efficiency. Their ability to balance exploration and exploitation has made them highly popular in diverse optimization tasks^18^.

MAs are typically inspired by many natural phenomena, including physical principles biological behaviors, human habits, and more. Various categories of MAs are present in the literature. In^19^, the authors classify MAs into two main groups: evolutionary algorithms and swarm intelligence algorithms. Furthermore, in^20^, the authors classify MAs into three distinct categories: evolutionary algorithms, swarm intelligence algorithms, and physical algorithms. On the other hand, the authors in^21^ categorize MAs as either single- or population-based solutions. Generally, there is no widely agreed upon criterion for categorizing metaheuristic algorithms. Nevertheless, the classification criteria that are most frequently employed are derived from a wide range of sources of inspiration. This work categorizes MAs into five broad groups: physical, evolutionary, swarm-based, mathematical, and human-based^22^. Evolutionary algorithms primarily imitate biological strategies, such as reproduction, genetic diversity, and mutational adaptation. The search process begins with a random population and then iterates constantly to accomplish multi-generational evolution. This category includes, for instance, Genetic Algorithm (GA)^23^, Differential Evolution (DE)^24^, and Liver Cancer Algorithm (LCA)^25^. The second category refers to mathematical algorithms^26^, for instance, Arithmetic Optimization Algorithm (AOA)^27^, Gradient Based Optimizer (GBO)^28^, the Weighted Mean of Vectors (INFO)^29^, and the Sine-Cosine Algorithm (SCA)^30^. The third category of algorithms refers to physics-based algorithms which replicate the behavior of physical events and their governing principles, such as magnetic fields, gravity, and mass equilibrium. The examples of this category include Simulated Annealing (SA)^31^, Gravitational Search Algorithm (GSA)^32^, Kepler Optimization Algorithm (KOA)^33^, Rime Optimization Algorithm (RIME)^34^, and. The fourth category pertains to human cooperation and behaviors within a society, referred to as human-based algorithms such as Teaching Learning-Based Optimization (TLBO)^35^, Human memory optimization algorithm (HMO)^36^, and Human evolutionary optimization algorithm (HEOA)^37^. The final category relates to swarm-based techniques, which are based on the collective behaviors of organisms in clusters, such as breeding, foraging, and hunting. This category includes a diverse range of algorithms such as Particle Swarm Optimization (PSO)^38^, Slime Mould Optimizer (SMA)^39^, Crayfish optimization algorithm (COA)^40^, Harris Hawks Optimization (HHO)^41^, Spider Wasp Optimizer (SWO)^42^, and Dung Beetle Optimization (DBO)^43^.

Despite their successes, many issues appear regarding MAs. Among the most important ones, there is the trade-off between exploration and exploitation. Exploration is the process by which the algorithm explores new, unexplored regions of the solution space in order to find multiple possible solutions. On the other hand, the exploitation phase generally needs the intensification of known promising solutions in order to achieve an optimum. A good balance between these two processes basically poses the challenge for any MA to be successful^44^. Additionally, MAs generally suffer from slow convergence rates, loss of accuracy as the problem becomes highly complex, and the tendency to get stuck in local optima, especially in high-dimensional space^45^. Due to these challenges regarding MAs, much research effort has focused on improving existing MAs by adding new strategies and hybridizing strategies from multiple algorithms^46^. The improvements aim at increasing the convergence speed, enhancing the solution’s accuracy, and enhancing the algorithm’s capability of escaping from local optima.

The Crayfish Optimization Algorithm (COA) is an innovative MA developed by Jia in 2023, inspired by the survival strategies observed in crayfish populations. This algorithm draws inspiration from crayfish behaviors such as avoiding heat, competing for shelter, and searching for food. Previous investigations have emphasized that, when compared to several traditional MA, its shared advantages include a versatile structure, a reduced number of parameter settings, and high accuracy. However, COA unavoidably has certain limitations, which is why this paper advocates for an enhanced version of COA. The main limitations of COA include: (1) The COA approach demonstrates insufficient accuracy and slow convergence when dealing with high-dimensional and non-convex issues. 2) when faced with complicated engineering optimization difficulties, COA is susceptible to getting stuck in local optima due to a large number of non-linear constraints. 3) the No Free Lunch (NFL) theorem^47^ states that “no MA can be guaranteed to work for all optimization applications” which motivates employing suitable tactics to enhance the efficiency and potential success of COA in addressing practical engineering problems.

To address these limitations, an Adaptive Dynamic Crayfish Optimization Algorithm with the improved escape operator, namely AD-COA-L, is proposed. Four main strategies are embedded in this variant of COA to enhance the performance of this algorithm:

- **Bernoulli Map Initialization**: This strategy is used in initialization so that a uniformly distributed population can be formed to enhance diversity from the initialization for the better exploration of the search space.
- **Adaptive Dynamic Inertia Weight**: This strategy updates the inertia weight dynamically in the exploitation phase to reserve the superior solutions and build up the search capability of the algorithm during iterations.
- **Local Escape Operator (LEO)**: LEO strengthens local exploitation with a view to strengthening information exchange between search agents, balancing exploration and exploitation, and hence enhancing the quality of the solution.
- **Adaptive Lens Opposition-Based Learning**: the ALOBL strategy moves the current best solution in the opposite direction, in later iterations, in order to avoid local optima and therefore increase the probability of global convergence.

In this regard, convergence speed, solution accuracy, and robustness of AD-COA-L have been strictly tested on 29 benchmark functions selected from the IEEE CEC2017 dataset. Also, this work compares the performance of the AD-COA-L with that of several state-of-the-art MAs. Furthermore, AD-COA-L is applied to seven constrained real-world engineering design problems to validate its practical effectiveness. The primary contributions are outlined as follows:

- An improved version of COA is proposed, AD-COA-L, which includes four major strategies to enhance the overall performance of COA including Bernoulli map initialization for diversity in the population, adaptive dynamic inertia weight for enhancing exploitation, local escape operator for improving local exploration, and ALOBL to prevent local optima.
- The strength of AD-COA-L is verified using 29 CEC2017 test functions. The acquired results are compared with several state-of-the-art methodologies and high-performance modified variant algorithms.
- The effectiveness of AD-COA-L in addressing intricate real-world optimization difficulties is confirmed by analysis of seven engineering design scenarios.
- The Wilcoxon rank-sum test and Friedman ranking test provide evidence that AD-COA-L outperforms other competing algorithms in terms of solution correctness, convergence rate, and resilience.

The subsequent sections of this study are structured as follows: Sect. 2 provides a summarized overview for the recent literature works. Section 3 provides an in-depth explanation of the principles and mathematical models that form the foundation of COA. Section 4 introduces the development of a sophisticated crayfish optimization algorithm called AD-COA-L, which utilizes multiple strategies to optimize its performance. The evaluation of the optimization performance of AD-COA-L on the CEC2017 benchmark suites is conducted in Sect. 5. Section 6 demonstrates the efficacy of AD-COA-L in seven real-world applications by presenting several examples of limited engineering design. Section 7 summarizes the result and presents possible directions for future research.

## Related work

Hu et al.^48^ introduced an enhanced hybrid AOA named CSOAOA to improve exploitation, avoid local optima, and increase convergence accuracy. CSOAOA incorporated point set initialization, optimal neighborhood learning, and crisscross optimization strategies. It was validated on 23 classical benchmark functions, CEC2019, and CEC2020 test suites, showing significant improvements in precision and convergence rate. Statistical tests confirmed that CSOAOA’s potential as a powerful algorithm for complex engineering optimization problems.

Shen et al.^49^ proposed MEWOA, a WOA variant using multi-population evolution to improve convergence speed and avoid local optima. MEWOA divided individuals into exploratory, exploitative, and modest sub-populations with different search strategies. It was tested on 30 benchmarks and real-world problems; MEWOA outperformed five WOA variants and seven metaheuristics in convergence speed, runtime, and solution accuracy, demonstrating its competitiveness. Qiao et al.^50^ proposed a hybrid AOA-HHO algorithm for Multilevel Thresholding Image Segmentation (MTIS) to improve threshold selection for object detection. Combining AOA’s exploration strengths with HHO’s exploitation abilities, AOA-HHO outperformed AOA, HHO, and other MAs. It used the image features as the fitness function, experiments on seven test images show superior segmentation accuracy, PSNR, SSIM, and execution time. Qiu et al.^51^ proposed an improved Gray Wolf Optimization (IGWO) algorithm to enhance the traditional GWO’s convergence speed, solution accuracy, and ability to escape local minima. IGWO used lens imaging reverse learning for initial population optimization, a nonlinear control parameter strategy, and tuning inspired by TSA and PSO. It was tested on 23 benchmarks, 15 CEC2014 problems, and 2 engineering problems; IGWO showed superior performance and balance in global optimization. Houssein et al.^52^ proposed mSTOA, an improved Sooty Tern Optimization Algorithm for feature selection (FS) to avoid sub-optimal convergence. mSTOA employed strategies for balancing exploration/exploitation, self-adaptive control parameters, and population reduction. It was validated on CEC2020 benchmarks and tested against various algorithms; mSTOA demonstrated superior performance in extracting optimal feature subsets, with statistical analyses confirming its effectiveness.

Wu et al.^53^ proposed a novel variant of the Ant Colony Optimization algorithm (MAACO) for mobile robot path planning to address slow convergence and inefficiency. MAACO introduced orientation guidance, an improved heuristic function, a new state transition rule, and uneven pheromone distribution. Experiments demonstrated MAACO’s superiority over 13 existing approaches in reducing path length, turn times, and convergence speed, proving its efficiency and practicality.

Nadimi-Shahraki et al.^54^ proposed an enhanced Whale Optimization Algorithm (E-WOA) using a pooling mechanism and three effective search strategies to address WOA’s low population diversity and poor search strategy. E-WOA outperformed existing WOA variants in solving global optimization problems. The binary version, BE-WOA, was validated on medical datasets, showing superior performance in feature selection, particularly for COVID-19 detection, compared to other high-performing algorithms.

Askr et al.^55^ proposed Binary Enhanced Golden Jackal Optimization (BEGJO) for feature selection (FS) to tackle high-dimensional datasets. BEGJO improved the original GJO by incorporating Copula Entropy for dimensionality reduction and four enhancement strategies to boost exploration and exploitation. It used the sigmoid transfer function where BEGJO outperformed other algorithms in classification accuracy, feature dimension, and ranks fourth in processing time, validated through statistical evaluations.

Ozkaya et al.^56^ proposed a novel Adaptive Fitness-Distance Balance based Artificial Rabbits Optimization (AFDB-ARO) algorithm to solve the complex Combined Heat and Power Economic Dispatch (CHPED) problem. AFDB-ARO enhanced exploration and balances exploitation, outperforming the base ARO in benchmark tests. It was applied to CHPED systems with various unit configurations, AFDB-ARO achieved optimal solutions in most cases, demonstrating superior performance and stability compared to ARO. Yıldız et al.^57^ proposed a novel hybrid optimizer, AOA-NM, combining Arithmetic Optimization Algorithm (AOA) and Nelder–Mead local search to improve solution quality and avoid local optima traps. AOA-NM’s performance was validated on CEC2020 benchmarks and ten constrained engineering design problems, showing superior results compared to other metaheuristics. Comparative analysis confirmed AOA-NM’s robustness in solving complex engineering and manufacturing problems. Deng et al.^58^ proposed an improved Whale Optimization Algorithm (IWOA) to address WOA’s slow convergence, low precision, and tendency to fall into local optima. IWOA used chaotic mapping for population initialization, integrates black widow algorithm pheromone and opposition-based learning for population modification, and employed adaptive coefficients and new update modes. It was tested on 23 benchmark functions; IWOA demonstrated superior convergence speed, stability, accuracy, and global performance compared to other optimization algorithms. Tan and Mohamad-Saleh^59^ proposed a hybrid Equilibrium Whale Optimization Algorithm (EWOA), combining bio-inspired WOA and Equilibrium Optimizer (EO). EWOA integrated WOA’s encircling and attacking mechanisms with EO’s weight balance strategy. It was tested on multiple benchmark sets; EWOA outperformed six state-of-the-art algorithms in terms of statistical mean performance, convergence rate, and robustness. EWOA achieved the best results on 46 out of 101 functions, demonstrating superior optimization efficiency. The Mahajan et al.^60^ proposed a hybrid method combining Aquila optimizer (AO) and AOA to enhance convergence and result quality. It was tested on various problems, including image processing and engineering design, AO-AOA demonstrated effectiveness in both high- and low-dimensional problems. The results showed efficient search results, particularly in high-dimensional problems, validating the approach. Qian et al.^61^ introduced a hybrid SSACO method that combines the foraging model of the salp swarm algorithm with the ant colony optimizer. The salp foraging behavior in SSACO effectively improved the original algorithm’s capacity to avoid local optima, resulting in a large increase in convergence accuracy. The application of SSACO to remote sensing image segmentation had yielded successful results. The evaluation of these results, based on peak signal-to-noise ratio, structural similarity index, and feature similarity index, had demonstrated that this method possessed distinct benefits over comparable segmentation methods.

Zhu et al.^62^ proposed the QHDBO algorithm, an enhanced Dung Beetle Optimization algorithm incorporating quantum computing and multi-strategy hybridization to address local optimum issues. QHDBO improved initial population distribution, balances global and local search, and used a t-distribution variation strategy. It was tested on 37 functions and engineering problems, QHDBO showed improved convergence speed, optimization accuracy, and robustness. Table 1 summarize the reviewed related and existing works to highlight the points of strength and weakness to motivate the need for the proposed work in this paper.

**Table 1:** Summary of existing works.

Related work	Methodology	Strength	Weakness
Hu et al. ^48^	Enhanced hybrid AOA (CSOAOA)	Improves exploitation, avoids local optima, increases convergence accuracy	Imbalanced exploration-exploitation despite improved accuracy, potential for slow convergence in high-dimensional problems
Shen et al. ^49^	Multi-population evolved WOA (MEWOA)	Increases convergence speed, avoids local optima, competitive performance	May face challenges in extremely high-dimensional problems despite improved convergence speed
Qiao et al. ^50^	Hybrid AOA-HHO for MTIS	Improves segmentation accuracy, PSNR, SSIM, execution time	Focused on image segmentation, lacks general applicability across other domains
Qiu et al. ^51^	Improved Gray Wolf Optimization (IGWO)	Improves convergence speed, solution accuracy, escapes local minima	Requires fine-tuning to maintain performance across diverse problems, risk of local optima
Houssein et al. ^52^	Improved Sooty Tern Optimization Algorithm (mSTOA)	Balances exploration/exploitation, avoids sub-optimal convergence	Control parameter sensitivity may lead to inconsistent performance in complex cases
Wu et al. ^53^	Variant Ant Colony Optimization (MAACO)	Reduces path length, turn times, improves convergence speed	High computational cost for large-scale problems despite improved path planning performance
Nadimi-Shahraki et al. ^54^	Enhanced Whale Optimization Algorithm (E-WOA)	Improves population diversity and search strategy	Struggles with maintaining balance in multi-objective tasks, relies heavily on parameter adjustment
Askr et al. ^55^	Binary Enhanced Golden Jackal Optimization (BEGJO)	Boosts exploration and exploitation, outperforms in classification accuracy	Computationally expensive, may not generalize well to larger datasets despite classification improvements
Ozkaya et al. ^56^	Adaptive Fitness-Distance Balance ARO (AFDB-ARO)	Balances exploration/exploitation, achieves optimal solutions	May struggle with large-scale problems despite performance in benchmark tests
Yıldız et al. ^57^	Hybrid AOA-NM	Improves solution quality, avoids local optima traps	Limited applicability outside constrained design problems
Deng et al. ^58^	Improved Whale Optimization Algorithm (IWOA)	Improves convergence speed, stability, accuracy	Challenges in dealing with complex constraints, potential slow convergence
Tan and Mohamad-Saleh ^59^	Hybrid Equilibrium Whale Optimization Algorithm (EWOA)	Superior statistical performance, convergence rate, robustness	Improved robustness but limited efficiency in more complex, high-dimensional spaces
Mahajan et al. ^60^	Hybrid AO-AOA	Effective in high- and low-dimensional problems	limited exploration in certain complex tasks
Qian et al. ^61^	Hybrid SSACO	Avoids local optima, improves convergence accuracy	Limited exploration capabilities in high-dimensional, non-convex problems
Zhu et al. ^62^	Enhanced Dung Beetle Optimization (QHDBO)	Improves convergence speed, accuracy, robustness	Still prone to local optima in extremely challenging problems despite overall improvements

According to the analysis of related works in Table 1, although performances of various MAs have enhanced over many reviewed related works, a lot of their shortcomings remain unsolved. Most of the available methods suffer from an imbalance between the exploration-exploitation principle, though they have converged to an optimal solution on certain problem domains. Besides, they often result in a phenomenon called premature convergence, when the algorithm converges into local optima without proper exploration of the solution space. Also, several related works, though improved in enhancing the speed of convergence, depict poor performance on complex, high-dimensional problems including a large and non-convex search space.

Furthermore, most of the works done previously are mainly dependent on fine-tuning control parameters toward optimal results. This very dependence makes these algorithms less general, with increased computational costs especially when it deals with large-scale or real-world applications. Their effectiveness is immensely reduced in problems of higher dimensions due to limited explorative capabilities.

The proposed AD-COA-L will directly address these gaps through the incorporation of a number of adaptive mechanisms. With the Bernoulli map, initialization is guaranteed to result in greater diversity of population at the very beginning. Adaptive dynamic inertia weight maintains a balance between exploration and exploitation in the process to ensure that neither of these phases ever dominates, hence avoiding premature convergence. The local escape operator enhances local exploration and allows the algorithm to move away from local optima. In addition, the ALOBL mechanism strengthens the exploration power of the algorithm for high-dimensional spaces. These merits of enhancement indicate that the new algorithm, namely AD-COA-L, will have better convergence, ensure the solution quality, and be more effective for complex, high-dimensional optimization problems compared to the previously developed algorithms.

## Crayfish optimization algorithm (COA)

In 2023, researchers introduced the COA^40^, which replicates crayfish behaviors: competitive behavior, summer resort behavior, and foraging behavior. These behaviors align with the exploitation and exploration phases of optimization, influenced by temperature. Higher temperatures lead crayfish to seek cave refuge for rest or competition, while suitable temperatures promote foraging during exploration. Temperature adjustments produce unpredictability in finding optimal solutions. The main stages of COA are follows:

- **Initialization**: In COA, an optimization problem with dimensions is represented by each crayfish, which serves as a potential solution in the form of a $\:1\times\:d$ vector. Each variable $\:({X}_{1},\:{X}_{2},\:{X}_{3},\:…,\:{X}_{d})$ represents a particular point $\:X$ within the search space, which is constrained by an upper boundary $\:Ub$ and a lower boundary $\:Lb$. During each iteration of the process, the most optimal solution is computed. The solutions are compared in a step-by-step manner, and the most favorable choice is found and retained as the ultimate optimal solution. The initial distribution of the COA population is established using Eq. (1):

1 $$\:{X}_{i}=Lb+(Ub+Lb)\times\:rand$$

where the optimization problem’s borders are represented by $\:Ub$ and $\:Lb$. The temperature is a crucial factor in multiple stages of the crayfish and is defined by Eq. (2). When the temperature exceeds 30 degrees, the crayfish relocates to a cooler area as its summer sanctuary. The crayfish exhibits its foraging activity when the temperature is suitable.

2 $$\:\text{\:temp\:}=\text{\:rand\:}\times\:15+20$$

Therefore, the act of searching for food can be replicated by employing a Gaussian distribution, which is influenced by the temperature as described in Eq. (3):

3 $$\:p={C}_{1}\times\:\left(\frac{1}{\sqrt{2\times\:\pi\:}\times\:\sigma\:}\times\:\text{e}\text{x}\text{p}\right)\left(\frac{(temp-\mu\:{)}^{2}}{2{\sigma\:}^{2}}\right)$$

where the temperature of the best crayfish is represented by $\:\mu\:$, whereas the parameters $\:{C}_{1}$ and $\:\sigma\:$ regulate the different temperatures of crayfish.

- **Summer resort phase**: In the summer, when the temperature exceeds 30 °C, crayfish actively seek out cool and moist tunnels to avoid the harmful effects of the heat. The method for determining these caverns is defined in Eq. (4):

4 $$\:{X}_{S}={(X}_{B}+{X}_{L})/2$$

According to Eq. (4), the best position is denoted as $\:{X}_{B}$, whereas the current position of the population is called $\:{X}_{L}$. Conversely, if the random number is below 0.5, there is no rivalry among the crayfish. Instead, they promptly assume possession of the cave in the following manner:

5 $$\:{X}_{new}={X}_{i}+{C}_{2}\times\:\:\text{r}\text{a}\text{n}\text{d}\:\times\:\left({X}_{S}-{X}_{i}\right)$$

6 $$\:{C}_{2}=2-\left(\frac{t}{T}\right)$$

where the position of the crayfish in the next iteration is represented as $\:{X}_{new}$, the position of the current crayfish is represented as $\:{X}_{i}$, and the maximum number of iterations is denoted as $\:T$.

- **Competition phase**: When the temperature exceeds 30 °C and the random variable rand is 0.5 or higher, it signifies that the crayfish are experiencing competition from other crayfish for the cave. The new position is calculated using Eq. (7):

7 $$\:{X}_{new}={X}_{i}-{X}_{z}+{X}_{\text{S\:}}$$

8 $$\:z=round(rand\times\:(N-1\left)\right)+1$$

*N* represents the total count of agents in the current population.

- **Foraging phase**: when the temperature reaches or falls below 30 °C, crayfish are prompted to leave their caves in order to search for food. At elevated temperatures, crayfish emerge from their burrows and locate food by utilizing the optimal place they determined during their evaluation. The food’s position is determined using the following:

9 $$\:{X}_{\text{F}}={X}_{B}$$

The consumption of crayfish is influenced by both their feeding rate and the size of the food they consume. If the food is overly large, the crayfish are unable to swallow it instantly; instead, they must first deconstruct it with their pincers. The size of the food is calculated using Eq. (10):

10 $$\:Q={C}_{3}\times\:\left(\frac{{F}_{i}}{{F}_{food}}\right)$$

where $\:{C}_{3}$ represents the maximum size of food, which is set at a specific value of 3. The variable $\:{F}_{i}$ represents the fitness score of the crayfish with the index , while $\:{F}_{food}$ represents the fitness score of the crayfish with the index and a specific food source.

Crayfish assess the magnitude of the meal by taking into account its maximal nutritional worth, $\:Q$, in order to select their feeding approach. If the value of exceeds ($\:{C}_{3}$ + 1)/2, it indicates that the food is too huge to be consumed directly. The formula for crushing food is as stated:

11 $$\:{X}_{\text{F\:}}=exp\left(-\frac{1}{Q}\right)\times\:{X}_{F}$$

Then, the crayfish employ their second and third claws to alternately grip the food and move it into their mouth. The equation representing the alternative feeding behavior of crayfish is given by Eq. (12):

12 $$\:{X}_{\text{new\:}}={X}_{i}+{X}_{\text{F\:}}\times\:p\times\:(cos(2\times\:\pi\:\times\:\:\text{r}\text{a}\text{n}\text{d}\:)-sin(2\times\:\pi\:\times\:\:\text{r}\text{a}\text{n}\text{d}\:\left)\right)$$

If the value of is less than or equal to ($\:{C}_{3}$ + 1)/2, it indicates that the crayfish may consume the meal instantly because it is an adequate size. The equation representing the feeding behavior of crayfish is given by Eq. (13):

13 $$\:{X}_{\text{new\:}}=\left({X}_{i}-{X}_{\text{F\:}}\right)\times\:p+p\times\:\:\text{r}\text{a}\text{n}\text{d}\:\times\:{X}_{i}$$

Finally, the greedy selection process is utilized to choose between the newly updated position and the present solution as follows:

14 $$\:{X}_{i}\left(t+1\right)=\left\{\begin{array}{c}{X}_{new}\:\:\:\:,\:\:\:\:\:\:\:if\:f\left({X}_{new}\right)<f\left({X}_{i}\right)\\\:{X}_{i}\:\:\:\:,\:\:\:\:\:\:\:\:\:\:\:\:\:\:\:\:\:\:\:\:\:\:\:\:\:\:\:otherwise\end{array}\right.$$

## The proposed AD-COA-L algorithm

This research presents a novel approach called AD-COA-L and utilizes it to address global optimization and engineering design challenges. Four main improvements guide the COA toward better solutions and obtain high quality fitness solutions. The details of these introduced strategies are explained in the following subsequent subsections.

### Bernoulli map-based population initialization

The fundamental aspect of metaheuristic algorithms lies in the iterative process of evaluating potential solutions. Consequently, the beginning population plays a crucial role in determining the algorithm’s convergence and exploration. Furthermore, it is widely recognized that the initialization phase of the majority of MAs involves generating random values within a specific range, following a Gaussian distribution. This initialization process has a significant impact on the progress and optimization quality. On the other hand, Chaotic maps are employed to produce chaotic sequences, which are sequences of unpredictability generated by straightforward deterministic systems. Chaotic maps exhibit non-linearity, a strong sensitivity to beginning conditions, ergodicity, randomness, chaotic attractors, fractional maintenance, overall stability, local instability, and long-term unpredictability. Thus, in the realm of optimization, chaotic maps are frequently employed as substitutes for the pseudo-random number generator $\:rand$ to produce chaotic numbers within the range of 0 to 1. Experimental evidence has shown that employing chaotic sequences for population initialization, selection, crossover, and mutation has a significant impact on the algorithm’s performance, typically resulting in superior convergence compared to utilizing random sequences^9^. The Bernoulli map is a common example of a chaotic system. The system is characterized as a segmented chaotic system, using the following formula:

15 $$\:Z(k+1,\gamma\:)=\left\{\begin{array}{ll}\frac{Z(k,\gamma\:)}{1-\gamma\:},&\:Z(k,\gamma\:)\in\:(\text{0,1}-\gamma\:]\\\:\frac{Z(k,\gamma\:)-1+\gamma\:}{\gamma\:},&\:Z(k,\gamma\:)\in\:(1-\gamma\:,1)\end{array}\right.$$

16 $$\:{S}_{i,j}={lb}_{j}+Z(k,\gamma\:)\times\:\left(u{p}_{i}-{lb}_{j}\right),\:i=\text{1,2},\dots\:,N,j=\text{1,2},\dots\:,D$$

where the parameter is a randomly chosen value between 0 and 0.5, typically with a value of 0.29 ^63^. The notation $\:{S}_{i,j}$ represents the $\:{j}^{th}$ dimension of the $\:{i}^{th}$ monochromatic wave.

In other words, the AD-COA-L introduces an initialization of the population based on the Bernoulli map with the aim of improving the exploration capability since the early stages of the optimization process. In the original COA, the population is initialized randomly within a fixed range and can result in some uneven or even suboptimal distribution of solutions. Such random initialization may imply the algorithm has only a limited capability of exploring the search space in depth, getting trapped into premature convergence to local optima.

In the random initialization, the highly sensitive initial condition-dependent chaotic sequence is now the Bernoulli map. The use of the Bernoulli map in the AD-COA-L guaranteed uniformity in the spread of population across the search space besides ensuring diversity. This strategy will enhance the quality of the population by generating a more diverse set of initial solutions, which enables the algorithm to explore more promising areas much earlier in the search process. This also helps to reduce the possibility of getting trapped into a local minimum, thereby helping to accelerate convergence toward the global optimum.

### Dynamic inertia weight coefficient

In the basic COA algorithm, the inertia weight value remains constant at 1. Consequently, the algorithm is prone to getting stuck in local minima. To address this issue, it has been recommended in^64^ to set the inertia weight value to a variable $\:w$ that is updated during iterations, leading to improved convergence. In this regard, the proposed AD-COA-L algorithm utilizes a variable value for the inertia weight coefficient, as described in Eq. 17:

17 $$\:w=\left|\text{c}\text{o}\text{s}\left(\frac{nt\pi\:}{T}\right)\right|$$

The adaptive inertia weight function, denoted as , is periodic function with represents a varied value, with possible values ranging from 1 to 0 in increments of 0.1. The variable $\:t$ represents the current iteration, while $\:T$ represents the maximum number of iterations. The inertia weight is added to both the competition and foraging phases of COA to boost the convergence speed at later iterations and helps AD-COA-L to avoid falling the local optima. The updated competition and foraging phases of AD-COA-L are represented by Eqs. (18–20) instead of Eqs. (7), (12) and (13).

18 $$\:{X}_{new}={w\times\:X}_{i}-{X}_{z}+{X}_{\text{S\:}}$$

19 $$\:{X}_{\text{new\:}}={w\times\:X}_{i}+{X}_{\text{F\:}}\times\:p\times\:(cos(2\times\:\pi\:\times\:\:\text{r}\text{a}\text{n}\text{d}\:)-sin(2\times\:\pi\:\times\:\:\text{r}\text{a}\text{n}\text{d}\:\left)\right)$$

20 $$\:{X}_{\text{new\:}}=w\times\:\left({X}_{i}-{X}_{\text{F\:}}\right)\times\:p+p\times\:\:\text{r}\text{a}\text{n}\text{d}\:\times\:{X}_{i}$$

The dynamic inertia weight coefficient not only enhances the exploration and exploitation capabilities of AD-COA-L but also ensures an effective balance between the two throughout the optimization process. The inertia weight is set to higher values in the early iterations in order to give more emphasis on global exploration. This higher value of inertia weight inspires the solutions to traverse a larger area of the search space; thus, the algorithm does not get entrapped into the local optima at the beginning. Enabling solutions to travel larger distances, AD-COA-L increases the chances of finding new diversified regions, hence reinforcing its exploration power.

As iterations grow and the algorithm starts to converge toward potential promising regions, the inertia weight starts to decrease. As a result, the algorithm now moves its focus from a broad exploration toward the exploitation of the best solutions found so far. A smaller inertia weight makes the search more local, which can enable the algorithm to fine-tune and refine the solutions in these high-potential regions. This refined search process amplifies the algorithm’s capability for higher accuracy and attainment of optimal solutions.

Furthermore, the balance between exploration and exploitation depends on the value of the probability parameter $\:p$ in the AD-COA-L algorithm. Higher values of $\:p$ in early stages allow wider explorations because it enables the solutions to make larger movements across the search space, thus preventing it getting stuck in local optima. Conversely, during runtime, if the value of $\:p$ is decreased, it guides the algorithm toward exploitation for more refined, local adjustments in the solutions for fine-tuning and optimization. This dynamic adjustment of $\:p$, combined with the adaptive inertia weight, maintains appropriate exploration-exploitation trade-offs so that the algorithm is always effectively exploring new areas while continually exploiting the best-found solutions for better convergence without getting stuck prematurely.

The AD-COA-L algorithm operates to keep a good balance between exploration and exploitation by varying the inertia weight dynamically with iteration count. It starts giving importance to wide exploration in its early iterations to ensure that the algorithm has scanned the solution space well and, in later stages, gives more importance to exploitation in order to tune the best-found solutions. This is important in avoiding premature convergence and maintaining efficient convergence toward global optima. Dynamic adjustment can assure that AD-COA-L will adaptively switch between exploration and exploitation to obtain more robust optimization performance.

### Adaptive lens reverse learning strategy

It is analyzed that the COA depends on the best solution $\:{X}_{B}$ during the position update of different phases as mentioned in Eqs. (5), (7) and (12) where new candidate solution is created by directing the current individual towards the global optimal point $\:{X}_{B}$. During the optimization process, the majority of individuals in the population have a tendency to gather around the perceived current best solution. Therefore, the COA is prone to early convergence. The primary research focus in improving COA is centered on enhancing its ability to overcome local optima. One widely used approach in the existing literature to strengthen the worldwide investigation of MAs is Opposition-Based Learning (OBL)^65^. The OBL algorithm is based on the concurrent calculation of objective values for the present individual and its inverse solution, in order to reveal a more advantageous optimal solution for the optimization objective. Nguyen et al.^66^ used the OBL mechanism into the Slime Mould Algorithm (SMA) to circumvent the occurrence of local optima and enhance the optimization performance for achieving optimal solutions.

**Fig. 1:**
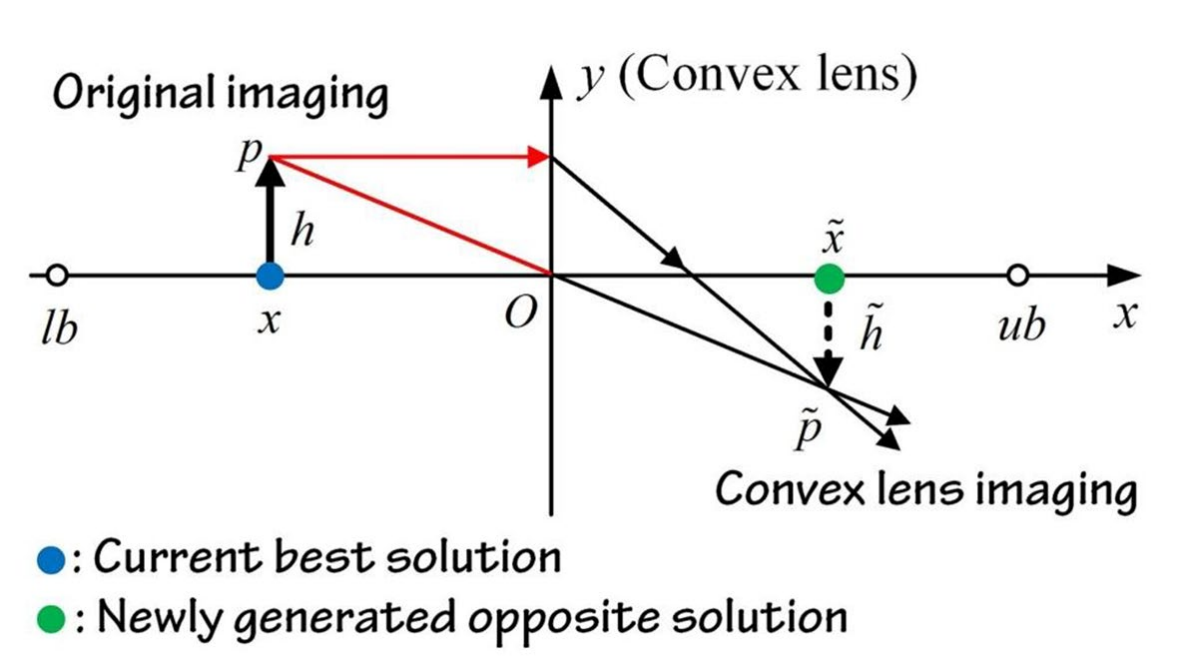
Schematic of Lens Opposite Based Learning.

On the other hand, Lens opposition-based learning (LOBL) is a novel adaptation of OBL that replicates the process of convex lens imaging in optical principles. More precisely, if an item is positioned at a distance equal to twice the focal length of a convex lens, a true image that is both inverted and reduced in size will be formed on the opposite side of the lens. In Fig. 1, the point $\:O$ represents the middle point of the search interval $\:[lb,\:ub]$ in a two-dimensional space. The y-axis is visualized as a convex lens. The assumption is that when a person with a height of $\:h$ is projected onto the x-axis in the image region, it is labeled as $\:x$. This $\:x$ point is located at a distance twice the focal distance away from the lens. Following the process of lens imaging, an actual image is formed with a height approximately equal to $\:\stackrel{\sim}{h}$. The projection of this image on the x-axis is denoted as$\:\:\stackrel{\sim}{x}$, indicated by the green point. By applying the fundamental principles of lens imaging, we can deduce the geometric equation as follows:

21 $$\:\frac{(lb+ub)/2-x}{\stackrel{\sim}{x}-(lb+ub)/2}=\frac{h}{\stackrel{\sim}{h}}$$

Let $\:k=h/\stackrel{\sim}{h}$, then Eq. (21) is converted into:

22 $$\:\stackrel{\sim}{x}=\frac{lb+ub}{2}+\frac{lb+ub}{2k}-\frac{x}{k}$$

When the value of k is equal to 1, Eq. (22) can be converted into the standard form of OBL in the following manner:

23 $$\:\stackrel{\sim}{x}=lb+ub-x$$

This implies that OBL is a specific example of LOBL, which not only possesses the benefits of OBL but also enhances solution variety and the probability of avoiding suboptimal solutions by adjusting the value of k. The extension of Eq. (23) to the D-dimensional space can be stated as follows:

24 $$\:{\stackrel{\sim}{x}}_{i,j}=\frac{l{b}_{j}+u{b}_{j}}{2}+\frac{l{b}_{j}+u{b}_{j}}{2k}-\frac{{x}_{i,j}}{k}$$

The variable $\:{\stackrel{\sim}{x}}_{i,j}$ denotes the opposing solution of the i-th individual in the j-th dimension. $\:l{b}_{j}$ and $\:u{b}_{j}$ represent the upper and lower limits in the j-th dimension, respectively.

**Fig. 2:**
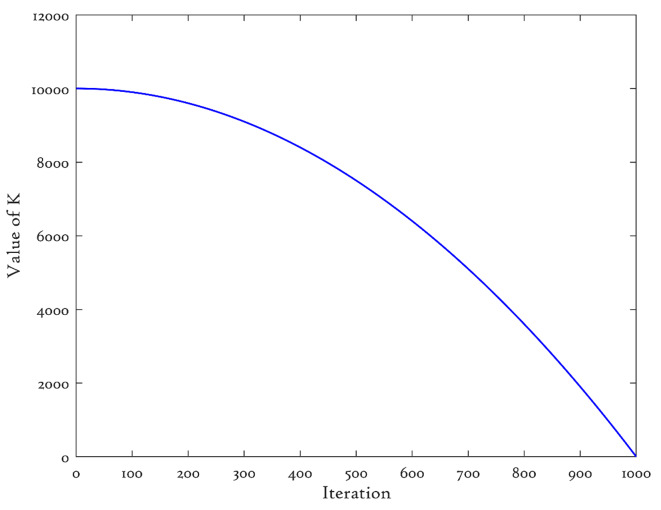
The value of proposed k with the progress of iterations.

In the basic LOBL, the variable $\:k$ is allocated a fixed value, which restricts its capacity to generate varied solutions during the iterations. The algorithm often prioritizes thorough exploration of the search space during the initial iterations in order to identify promising areas that contain optimal answers. Currently, increasing the value of $\:k$ significantly can enhance the search breadth and population diversity. During the later stages, a reduced $\:k$ value can be employed to improve the local search efficiency of the algorithm, resulting in a more accurate optimal solution. Thus, this paper suggests a nonlinear adaptive reduction mechanism for modifying the value of $\:k$ resulting in adaptive variant of LOBL named Adaptive Lens Opposite-based Learning (ALBOL) in which the parameter $\:k$ is defined in the following manner:

25 $$\:k={10}^{4}\times\:\left[1-{\left(\frac{t}{T}\right)}^{2}\right]+1$$

where $\:t$ represents the current iteration and $\:T$ represents the maximum number of iterations. Figure 2 illustrates the trajectory of the variable $\:k$. After completing all algorithm operations, the proposed ALOBL mechanism is utilized to gradually modify the current optimal solution $\:{X}_{B}$ dimension by dimension. This adjustment aims to bring the solution closer to the theoretical optimal solution and speed up the convergence process.

In other words, the strategy of ALOBL in AD-COA-L is applied only for the current best solution in the population, to avoid falling into the local optima strategically. The ALOBL generates an opposite solution to the current best solution by reflecting the best solution across the midpoint of the search space to create an alternative solution that explores another area of space that may lead to better optima. The application of ALOBL in this regard ensures that the algorithm does not disrupt the progress of the whole population, but indeed provides a critical exploration mechanism for the most promising candidate.

In addition, the ALOBL will be adaptive is the opposition strength, controlled by dynamically changing parameter $\:k$. Because this value of the parameter is higher at early iterations, it maintains a higher diversity in the opposite solutions that enable broader exploration. Furthermore, $\:k$ value fine-tunes the search to improve the exploitation around the best-found areas. When ALBOL applied to the best solution, AD-COA-L efficiently balances exploration and exploitation, leading to a better convergence behavior of this approach without the risk of premature stagnation in suboptimal regions. This strategy enhances the ability of the algorithm to pass through a complex search space and accelerates its convergence with maintained diversity in solutions.

### Local escaping Operator (LEO)

The Local Escaping Operator (LEO) is an additional local search algorithm introduced in^28^. Its main purpose is to enhance the exploration capabilities of the Gradient-based Optimizer by facilitating the exploration of new regions, especially in complex real-world problems. This leads to an improvement in the overall quality of the solution. LEO updates the positions of solutions based on specific criteria, effectively preventing the optimization algorithm from being trapped in local optima and improving its convergence behavior. To generate alternative solutions with superior performance, LEO utilizes critical solutions, including the best position $\:{X}_{B}$, two randomly generated solutions $\:{X}_{r1}$ and $\:{X}_{r2}$, two randomly chosen solutions $\:X{1}_{i}$ and $\:X{2}_{i}$, and a newly generated random solution $\:{X}_{z}$. The following scheme provides a mathematical formula for determining the value of $\:{X}_{LEO}$:

if rand < pr then

if rand < 0.5 then.

$$\:{X}_{LEO}\left(t\right)\leftarrow\:{X}_{i}(t+1)+{f}_{1}\times\:\left({u}_{1}\times\:{X}_{\text{best\:}}-{u}_{2}\times\:{\text{X}}_{\text{z}}\left(t\right)\right)+{f}_{2}\times\:{\rho\:}_{1}\times\:({u}_{3}\times\:\left(X{2}_{i}-X{1}_{i}\right)+{u}_{2}\times\:\left({X}_{r1}-{X}_{r2}\right))/2$$

$$\:{X}_{i}(t+1)\leftarrow\:{X}_{LEO}\left(t\right)$$

else.

$$\:{X}_{LEO}\left(t\right)\leftarrow\:{X}_{best}+{f}_{1}\times\:\left({u}_{1}\times\:{X}_{\text{best\:}}-{u}_{2}\times\:{\text{X}}_{\text{z}}\left(t\right)\right)+{f}_{2}\times\:{\rho\:}_{1}\times\:({u}_{3}\times\:\left(X{2}_{i}-X{1}_{i}\right)+{u}_{2}\times\:\left({X}_{r1}-{X}_{r2}\right))/2$$

$$\:{X}_{i}(t+1)\leftarrow\:{X}_{LEO}\left(t\right)$$

end if.

end if (26)

The given equations have several parameters including $\:f1$ which is a stochastic variable that can assume any value between − 1 and 1 inclusively, $\:f2$ is a random variable that follows a normal distribution with a mean of 0 and a standard deviation of 1. Furthermore, $\:{\rho\:}_{1}$ indicates the probability and there are three additional random variables, specifically ($\:{u}_{1}$, $\:{u}_{2}$, and $\:{u}_{3}$) which are defined as follows:

27 $$\:{u}_{1}=\left\{\begin{array}{ll}2\times\:\text{\:rand\:}&\:\text{\:if\:}{\mu\:}_{1}<0.5\\\:1&\:\text{\:otherwise\:}\end{array}\right.$$

28 $$\:{u}_{2}=\left\{\begin{array}{ll}\text{\:rand\:}&\:\text{\:if\:}{\mu\:}_{1}<0.5\\\:1&\:\text{\:otherwise\:}\end{array}\right.$$

29 $$\:{u}_{3}=\left\{\begin{array}{ll}\text{\:rand\:}&\:\text{\:if\:}{\mu\:}_{1}<0.5\\\:1&\:\text{\:otherwise\:}\end{array}\right.$$

where $\:rand$ represents a randomly generated number that falls within the range of 0 to 1. On the other hand, the variable $\:\mu\:$ represents a number that also falls within the range of 0 to 1. The provided equations can be simplified as shown in Eqs. (30–32):

30 $$\:{u}_{1}={Q}_{1}\times\:2\times\:\text{r}\text{a}\text{n}\text{d}+\left(1-{Q}_{1}\right)$$

31 $$\:{u}_{2}={Q}_{1}\times\:\text{r}\text{a}\text{n}\text{d}+\left(1-{Q}_{1}\right)$$

32 $$\:{u}_{3}={Q}_{1}\times\:\text{r}\text{a}\text{n}\text{d}+\left(1-{Q}_{1}\right)$$

The binary parameter, $\:{Q}_{1}$, can only have a value of either 0 or 1. This value is determined by a condition: if $\:{Q}_{1}$ is less than 0.5, then $\:{Q}_{1}$ is set to 1. Alternatively, it is given a value of 0. In addition, to maintain a proper equilibrium between exploration and exploitation in search processes, the variable $\:{\rho\:}_{1}$ is introduced which is defined by Eqs. (33–35):

33 $$\:{\rho\:}_{1}=2\times\:\text{r}\text{a}\text{n}\text{d}\times\:\alpha\:-\alpha\:$$

34 $$\:\alpha\:=\left|\text{s}\text{i}\text{n}\left(\text{s}\text{i}\text{n}\left(\beta\:\times\:\frac{3\pi\:}{2}\right)+\frac{3\pi\:}{2}\right)\times\:\beta\:\right|$$

35 $$\:\beta\:=\left({\beta\:}_{\text{m}\text{a}\text{x}}-{\beta\:}_{\text{m}\text{i}\text{n}}\right)+{\beta\:}_{\text{m}\text{i}\text{n}}\times\:{\left(1-{\left(\frac{t}{\text{T}}\right)}^{3}\right)}^{2}$$

where the values of $\:{\beta\:}_{min}\:$ and $\:{\beta\:}_{max}$are fixed at 0.2 and 1.2, respectively. The variable denotes the present iteration, while $\:T$ signifies the maximum number of iterations. In order to maintain an equilibrium between exploration and exploitation, the parameter $\:{\rho\:}_{1}$ automatically adapts itself according to the sine function $\:\alpha\:$. The parameters $\:{\beta\:}_{min}$ and $\:{\beta\:}_{max}$​ influence the adaptation of the probability factor $\:\rho\:1$ inside the strategy of LEO. These modulate the function $\:\alpha\:$ of the sine that applies the perturburbation step to the solutions. The smaller value of $\:{\beta\:}_{min}$, in the initial iterations, promotes wider exploration. A higher value of $\:{\beta\:}_{max}$ during the ending iterations gives smaller, finer perturbations, shifting the focus toward exploitation. The adaptive mechanism provides an effective balance between exploration and exploitation, improves the convergence, and gives more accurate solutions using the AD-COA-L algorithm. It is proposed to calculate the solution, $\:{X}_{z}$, in the prior scheme by following the indicated strategy in Eqs. (36) and (37):

36 $$\:{X}_{z}=\left\{\begin{array}{ll}{X}_{\text{rand\:}}&\:\text{\:if\:}{\mu\:}_{2}<0.5\\\:{\text{X}}_{\text{p}}&\:\text{\:otherwise\:}\end{array}\right.$$

37 $$\:{X}_{\text{rand\:}}={X}_{\text{min\:}}+\text{r}\text{a}\text{n}\text{d}\left(\text{0,1}\right)\times\:\left({X}_{\text{max\:}}-{X}_{\text{min\:}}\right)$$

where $\:{X}_{rand}$ denotes a fresh generated solution, while $\:{X}_{p}$ refers to a solution that has been chosen randomly from a population. Additionally, $\:\mu\:$ represents a random number that falls within the range of values between 0 and 1. Equation (37) can be simplified in the following manner:

38 $$\:{X}_{z}={Q}_{2}\times\:{X}_{p}+\left(1-{Q}_{2}\right)\times\:{X}_{\text{rand\:}}$$

Here, the parameter $\:{Q}_{2}$ is a binary variable that can only take the values of 0 or 1. Its value is decided by whether the variable $\:\mu\:$ is smaller than 0.5 or not. The stochastic selection of parameter values $\:{u}_{1}$, $\:{u}_{2}$, and $\:{u}_{3}$ enhances population variety and aids in avoiding local optimal solutions.

COA may struggle to achieve optimal performance as a result of insufficient information sharing among individuals. Relying solely on the dominant solution for guidance is a type of greedy search, which increases the likelihood of becoming trapped in local minima. To effectively discourage the deployment of isolated solutions and encourage the exchange of information in the search area, it is imperative for all participating parties to maintain communication via harnessing collective intelligence. In order to address this problem, the AD-COA-L algorithm utilizes the LEO operator at the end of each iteration to enhance the exploitation and search capabilities of COA. Additionally, the LEO strategy provides a controlled perturbations to the solutions’ positions by stochastic variables and probability factors. This leads to easily escaping local minima, encouraging the algorithm to explore parts of the search space not analyzed before. This could render LEO particularly effective during later iterations when the algorithm can refine the search with a maintained diversity in the population. Enriching its general convergence speed and solution accuracy to enable the algorithm to plunge even into global optima for highly complex optimization problems, AD-COA-L is applied with LEO at the end of every iteration.

**Fig. 3:**
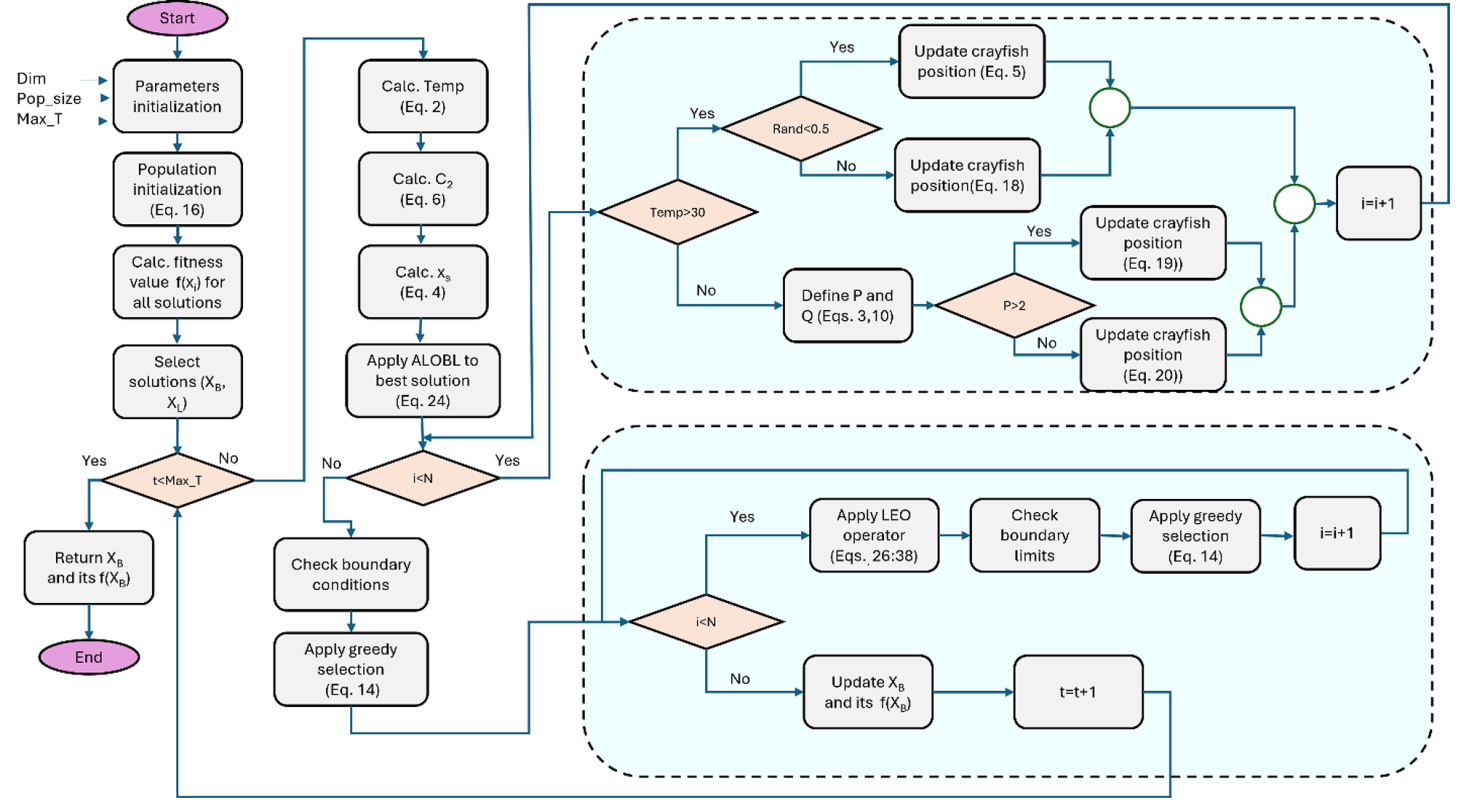
The proposed AD-COA-L algorithm.

Consequently, the new AD-COA-L allows the population to discard inefficient options and perform the local search process more efficiently. The main steps and operators of the proposed AD-COA-L is depicted in Algorithm 1 and Fig. 3.

**Table:** 

Algorithm 1: AD-COA-L algorithm	
**Input**: Maximum number of iterations $\:T$, Population size $\:N$. **Output**: Optimal solution $\:{X}_{B}$, fitness value of optimal solution $\:{f}_{B}$	
1.	Initialize the initial population $\:X$ using Eq. (16)
2.	Compute the fitness value $\:f\left({X}_{i}\right)$ of each solution
3.	Obtain the required solutions $\:{X}_{B}$ and $\:{X}_{L}\:$
4.	**for** $\:t\le\:\text{T}$ **do**
5.	Calculate the temperature $\:temp$ using Eq. (2)
6.	Calculate $\:{C}_{2}$ using Eq. (6)
7.	Calculate $\:{X}_{S}$ using Eq. (4)
8.	**Apply ALOBL strategy to the best solution using** Eq. (24)
9.	$\:\mathbf{f}\mathbf{o}\mathbf{r}\:i=1\:\text{t}\text{o}\:N\:\mathbf{d}\mathbf{o}$
10.	**if** $\:temp$ $\:>30$ **then**
11.	**if** $\:rand<0.5$ **then**
12.	Update position of crayfish using Eq. (5)
13.	**Else**
14.	Update position of crayfish using Eq. (18)
15.	**end if**
16.	**else**
17.	Define the food intake $\:p$ and size $\:Q$ using Eq. (3) and Eq. (10)
18.	**if** $\:p>2$ **then**
19.	Update the position of crayfish using Eq. (19)
20.	**else**
21.	Update position of crayfish using Eq. (20)
22.	**end if**
23.	**end if**
24.	**end for**
25.	Check the boundary conditions
26.	Apply greedy selection using Eq. (14)
27.	$\:\mathbf{f}\mathbf{o}\mathbf{r}\:i=1\:\text{t}\text{o}\:N\:\mathbf{d}\mathbf{o}$
28.	**Apply LEO operator using** Eqs. (26–38)
29.	Check boundary limits
30.	Apply greedy selection using Eq. (14)
31.	**end for**
32.	Update the current optimal solution $\:{X}_{B}$ and its fitness value $\:{f}_{B}$.
33.	$\:t=t+1$
34.	**end for**
35.	**Return**$\:{X}_{B}$ and its fitness value $\:{f}_{B}$;

## Experimental results and analysis

This study evaluates the effectiveness of the AD-COA-L algorithm by comparing it against eleven conventional and recent algorithms, as well as eight state-of-the-art similar algorithms that are recognized for their exceptional performance. A total of 29 benchmark functions from CEC2017 ^67^ were tested. The comparison includes six conventional algorithms: Particle Swarm Optimizer (PSO)^38^, Sine-Cosine Algorithm (SCA)^30^, Slime Mould Optimizer (SMA)^39^, Arithmetic Optimization Algorithm (AOA)^27^, Whale Optimization Algorithm (WOA)^68^, and Harris Hawk Algorithm (HHO)^41^. Additionally, it incorporates five recent algorithms: White Shark Optimizer (WSO)^69^, Gradient Based Optimizer (GBO)^28^, Spider Wasp Optimizer (SWO)^42^, Weighted Mean of Vectors (INFO)^29^, and the original COA. The specifications for the comparison methods’ parameters can be found in Table 2. In order to achieve fairness, every algorithm is given a consistent maximum iteration limit of 1000 and an initial population size of 30. To minimize variability, every algorithm is run 30 times for each test function, and the standard deviation (STD) and average (AVG) of the results are recorded.

The trials are conducted on a system including an Intel(R) i7-10750 H CPU, 32 GB of RAM, and running Microsoft Windows 10. Furthermore, MATLAB (R2020a) functions as the programming environment for coding, ensuring dependability and computational power throughout experimentation. The rows in the Tables indicate the ranking of the average values. A rank of 1 signifies that the algorithm achieved the lowest average solution value out of 30 trials, indicating a higher search capability.

**Table 2:** Comparative algorithms parameters’ values.

Algorithm	Setting values
PSO	$\:W=[0.4,\:0.9],\:c1=c2=2$
AOA	$\:MOP\:limit=\text{0.2,1}$ $\:\alpha\:=5,\:\mu\:=0.499$
WOA	$\:Q=[-1,\:1]\:and\:k=1$
SCA	$\:A\:is\:set\:to\:2$
WSO	$\:fmax=0.75\:and\:fmin=0.07$
SWO	$\:TR=0.3\:and\:CR=0.2$
COA, AD-COA-L	$\:C3=3,\:\mu\:=25,\:\sigma\:=3$
SMA	$\:z=0.03,\:p=0.03$
AOA	$\:{C}_{1}=2,{C}_{2}=6,{C}_{3}=2,\:{C}_{4}=0.5$
INFO	$\:c=2,\:d=4$
GBO	$\:{\beta\:}_{min}=0.2,\:{\beta\:}_{max}=1.2,\:pr=0.5$

### Parameter sensitivity analysis

Two main parameters affect the performance of AD-COA-L including the probability $\:p$ and the LEO limit parameters βmin and βmax. Therefore, in this section an experiment is conducted to test the sensitivity analysis of these parameters. First, we perform the sensitivity analysis of the AD-COA-L algorithm with respect to the probability parameter $\:p$, that controls the size of position updates in both competitive and foraging phases. To this end, we conducted experiments over 29 benchmark functions of CEC 2017 suite with nine different values of p within the interval [0.1, 0.9] with a step size of 0.1. Table 3 reports the STD and AVG of different variations of parameter $\:p$. In all functions, the results include the computation of the average fitness AVG and STD over multiple runs and present the results for each value of $\:p$. Further, performance across all functions has been ranked based on the Friedman rank test where lower ranks correspond to a better overall performance. The well marked variation of performance when the value of $\:p$ changes are reported. For instance, for lower values of $\:p$, such as 0.1 and 0.2, the algorithm tends to explore more widely, which indicates that with larger steps, while the exploration capability of the algorithm is wider in the search space, convergence towards the optimal solution is less precise.

Because $\:p$ increases to 0.3 and 0.4, the drastic improvement of the algorithm’s performance is evident. In particular, when $\:p$ = 0.4, the lowest AVG values for many benchmark functions compared to other variations are obtained by the algorithm, where the STD values of most benchmark functions depict steady convergence behavior. The fact that $\:p$=0.4 presents the best performance is further corroborated by the Friedman rank, reaching the minimum average rank there, which maintains an effective balance between exploration and exploitation. This lets the algorithm efficiently explore the search space and fine-tune solutions in later iterations.

Beyond $\:p$ = 0.4, the performance of the algorithm starts to degrade slightly, which could also be seen from increased AVG and STD values when $\:p$ = 0.5 and beyond. That is indicative of the fact that the higher values of $\:p$ shift the algorithm to a more conservative strategy of search in favour of exploitation at the expense of global exploration. Thus, the algorithm is then prone to local optima, particularly on multimodal functions, which call for a broader search.

This sensitivity analysis introduces that the selection of probability parameter $\:p$ significantly influences the performances of the AD-COA-L algorithm. In this work, the optimal value identified is $\:p$=0.4, since the optimal trade-off between exploration and exploitation occurs for the algorithm, with superior performance evident over most of the CEC2017 benchmark functions. Therefore, in applications of the AD-COA-L algorithm, the utilization of $\:p$=0.4 will be taken into consideration to ensure robustness in the performances of optimization.

**Table 3:** Sensitivity analysis of parameter *p*

F		*p*=0.1	*p*=0.2	*p*=0.3	*p*=0.4	*p*=0.5	*p*=0.6	*p*=0.7	*p*=0.8	*p*=0.9
F1	AVG	2914.970	2790.631	2370.477	2016.222	3080.839	2745.107	2984.092	1955.950	**1785.844**
	STD	3067.888	2621.845	2365.965	2739.999	3037.802	2842.528	2638.295	**1801.859**	1842.695
F3	AVG	300.000	300.000	300.000	**300.000**	300.000	300.000	300.000	300.000	300.000
	STD	0.000	0.000	0.000	**0.000**	0.000	0.000	0.000	0.000	0.000
F4	AVG	404.996	402.433	402.154	402.114	404.574	**402.061**	404.482	402.152	404.481
	STD	12.157	0.828	1.080	0.970	12.028	1.074	12.750	**0.799**	11.754
F5	AVG	514.725	515.678	512.674	511.543	514.213	515.160	512.533	**510.604**	511.504
	STD	6.816	6.507	**4.764**	5.751	6.352	6.554	7.207	5.459	5.650
F6	AVG	600.005	600.007	600.007	**600.001**	600.007	600.026	600.004	600.066	600.074
	STD	0.022	0.029	0.029	**0.002**	0.019	0.119	0.011	0.260	0.389
F7	AVG	732.884	730.476	729.012	727.287	728.611	**727.268**	728.482	729.400	727.354
	STD	12.888	9.431	8.066	8.166	**6.564**	8.052	9.762	8.336	8.656
F8	AVG	820.297	818.175	816.649	816.483	815.787	818.308	816.417	820.562	**813.797**
	STD	5.210	5.616	5.969	5.535	6.313	**4.941**	6.747	9.487	6.951
F9	AVG	900.570	**900.091**	900.488	900.261	900.231	900.258	900.148	900.106	900.284
	STD	2.781	**0.346**	1.202	0.728	0.589	0.710	0.515	0.371	1.091
F10	AVG	1796.412	**1720.963**	1773.530	1783.348	1822.633	1840.329	1868.146	1855.204	1825.656
	STD	324.757	278.732	281.498	280.044	297.047	235.682	**204.305**	295.473	244.190
F11	AVG	1114.407	1110.341	1111.510	1109.740	**1108.841**	1111.268	1111.852	1109.778	1112.042
	STD	10.635	7.742	11.686	5.510	6.599	10.989	6.577	**5.377**	7.786
F12	AVG	1.109E + 04	1.243E + 04	**1.099E + 04**	1.294E + 04	1.448E + 04	1.193E + 04	1.359E + 04	1.188E + 04	2.482E + 04
	STD	**7.557E + 03**	9.866E + 03	9.934E + 03	1.045E + 04	1.080E + 04	8.517E + 03	1.152E + 04	9.139E + 03	4.752E + 04
F13	AVG	7319.643	9452.287	7555.361	7322.329	7960.247	6937.152	7433.426	**6853.058**	7646.979
	STD	5166.980	5813.553	4917.643	5548.804	4399.696	**4333.280**	4867.852	5112.088	4662.249
F14	AVG	1456.690	1463.982	1456.455	1464.498	1466.125	**1455.323**	1467.344	1466.175	1511.596
	STD	**23.494**	38.935	23.761	35.069	31.725	33.837	28.646	44.535	164.483
F15	AVG	**1539.660**	1581.334	1580.945	1661.276	1587.780	1596.697	1611.015	1583.903	1671.012
	STD	**32.877**	88.755	67.285	183.605	80.153	77.588	121.319	103.876	389.450
F16	AVG	1715.579	1722.061	1692.191	1679.430	1698.432	1688.219	1668.952	1698.046	**1666.663**
	STD	117.040	107.498	109.071	**83.413**	103.975	120.341	95.413	118.366	83.824
F17	AVG	1737.922	1736.327	**1735.392**	1738.064	1737.101	1741.193	1743.505	1747.238	1740.305
	STD	20.574	18.010	19.442	21.392	15.741	18.604	19.466	28.317	**15.090**
F18	AVG	1.029E + 04	9.393E + 03	1.211E + 04	1.059E + 04	1.019E + 04	1.221E + 04	**9.135E + 03**	1.169E + 04	9.884E + 03
	STD	9.197E + 03	7.682E + 03	1.051E + 04	8.706E + 03	**7.206E + 03**	1.233E + 04	8.077E + 03	9.703E + 03	9.452E + 03
F19	AVG	4635.888	5114.007	4388.294	5825.227	4620.957	3934.643	5413.622	5432.556	**3798.480**
	STD	2913.909	3773.489	3528.607	5801.014	4399.353	**2540.750**	3678.078	4216.590	2759.922
F20	AVG	2058.041	2060.533	2058.633	2059.613	2068.792	2064.003	2064.347	**2056.833**	2068.118
	STD	53.794	53.131	55.057	60.764	60.269	54.967	75.268	61.240	**48.689**
F21	AVG	2274.929	2263.345	2263.778	2228.304	2227.895	2231.466	2250.757	2256.763	**2220.703**
	STD	57.802	58.590	58.888	47.797	48.363	49.932	56.655	59.820	**41.094**
F22	AVG	2301.898	2298.926	2296.440	2296.126	**2295.419**	2301.779	2295.712	2295.696	2301.204
	STD	0.904	13.129	19.487	20.576	24.472	1.035	21.865	23.053	**0.558**
F23	AVG	**2614.667**	2617.113	2615.311	2615.403	2616.685	2616.896	2615.965	2616.408	2618.894
	STD	5.862	10.113	6.157	7.735	7.847	7.181	**5.746**	6.039	9.044
F24	AVG	2748.502	2738.693	2739.975	2738.123	2750.403	2749.353	**2721.769**	2722.450	2739.040
	STD	8.604	45.496	45.839	45.649	8.502	**7.064**	75.416	75.860	45.711
F25	AVG	2929.767	2926.761	2927.735	2932.863	2928.393	**2926.720**	2934.886	2926.951	2931.257
	STD	29.382	23.555	22.509	**20.706**	22.824	23.114	23.166	23.681	21.769
F26	AVG	2918.634	**2896.783**	2962.660	2955.677	2903.703	2907.698	2967.895	2898.328	2898.653
	STD	206.513	79.111	207.953	241.552	53.876	52.042	273.238	**48.618**	78.304
F27	AVG	3097.828	3098.844	3099.497	3092.709	3092.203	3093.596	**3091.954**	3095.301	3092.807
	STD	17.995	22.933	17.283	2.631	**2.322**	10.989	2.632	16.475	3.088
F28	AVG	3330.768	3359.143	3368.113	**3318.037**	3349.360	3380.123	3355.468	3350.177	3362.445
	STD	153.060	160.850	153.606	159.558	118.861	**118.293**	143.109	186.773	181.344
F29	AVG	3208.716	3199.846	3210.029	3219.014	3208.830	3198.291	**3198.231**	3198.817	3200.998
	STD	**33.125**	45.961	63.445	59.219	48.905	45.834	53.011	46.787	42.707
F30	AVG	1.681E + 05	2.379E + 05	**1.053E + 05**	4.641E + 05	3.048E + 05	5.831E + 05	3.527E + 05	4.640E + 05	2.400E + 05
	STD	3.072E + 05	4.152E + 05	**2.088E + 05**	9.019E + 05	5.297E + 05	9.073E + 05	4.265E + 05	6.565E + 05	2.638E + 05
FR rank		5.41	5.11	4.93	**4.48**	5.01	4.89	5.08	4.87	5.22

Together with the sensitivity analysis of the probability parameter $\:p$, an experiment also will be carried out that analyzes the impact of the parameters $\:{\beta\:}_{min}$d $\:{\beta\:}_{max}$. This would give which governs the adaptation of the probability factor $\:\rho\:1$ within LEO strategy. Six different combinations of $\:{\beta\:}_{min}$ and $\:{\beta\:}_{max}$re evaluated, described in detail in Table 4.

**Table 4:** Different scenarios for the parameters $\:{\beta\:}_{min}$ and $\:{\beta\:}_{max}$

Scenario	βmin	βmax
Scenario 1	0.2	1.0
Scenario 2	0.2	1.1
Scenario 3	0.2	1.2
Scenario 4	0.3	1.0
Scenario 5	0.3	1.1
Scenario 6	0.3	1.2

Additionally, Table 5 captures the STD and AVG values of different combinations for the parameters $\:{\beta\:}_{min}$ and $\:{\beta\:}_{max}$. The sensitivity analysis has shown that the algorithm’s performance significantly depends on the choice of $\:{\beta\:}_{min}$ and $\:{\beta\:}_{max}$. Among the six scenarios, the best performance of the algorithm is obtained for Scenario 3, since this scenario has minimum AVG and STD values for many of the CEC 2017 benchmark functions compared to other scenarios. It finds reflection in its Friedman rank of 1.87, thereby confirming that it has the best balance between exploration and exploitation, which has translated into better convergence behavior.

In contrast, the AVG and STD values were higher for Scenario 1 since the smaller range of $\:\beta\:$ resulted in limited exploration. The Friedman rank achieved for this scenario is 4.62, indicating relatively poor performance. Similar behavior was obtained for Scenario 4 with a Friedman rank of 4.28, since the increase of βmin to 0.3 yielded poor exploration capabilities of the algorithm in the search space.

Scenario 2 and Scenario 5 provided a moderate performance, ranking Friedman at 3.43 and 3.15, respectively. While they turned out better than the results for Scenario 1, their balance between exploration and exploitation was still far from being as ideal as in Scenario 3. In Scenario 6, the wider range allowed the Friedman rank to become as low as 2.65 due to effective exploration and exploitation of $\:\beta\:$, though less well-balanced compared to the Scenario 3 configuration.

The Friedman rank test categorizes Scenario 3 as the best among all parameter combinations tested. Therefore, the sensitivity analysis conducted within the study revealed that the choices of $\:{\beta\:}_{min}$ and $\:{\beta\:}_{max}$ are crucial for the performance delivered by AD-COA-L. The best performing variants were, in fact, obtained by the setting introduced in Scenario 3 with Friedman rank of 1.87 reached a good balance between exploration and exploitation, resulting in enhanced optimization performance of the benchmark functions.

**Table 5:** Sensitivity analysis of the parameters $\:{\beta\:}_{min}$ and $\:{\beta\:}_{max}$

F		Scenario 1	Scenario 2	Scenario 3	Scenario 4	Scenario 5	Scenario 6
F1	AVG	3249.980	**1884.484**	2564.724	2602.047	2316.105	2083.979
	STD	3063.281	2473.937	2616.219	2956.188	2332.908	**2061.324**
F3	AVG	300.000	300.000	**300.000**	300.000	300.000	300.000
	STD	0.000	0.000	**0.000**	0.000	0.000	0.000
F4	AVG	406.615	404.886	404.641	404.388	404.880	**402.083**
	STD	16.270	11.749	12.601	12.025	12.232	**0.998**
F5	AVG	517.001	517.864	516.981	520.363	519.023	**516.350**
	STD	**6.867**	9.922	8.748	9.621	8.699	7.639
F6	AVG	600.095	600.048	**600.001**	600.047	600.040	600.066
	STD	0.408	0.255	**0.005**	0.214	0.170	0.204
F7	AVG	**729.866**	732.022	730.430	731.410	730.620	731.164
	STD	8.225	12.356	**7.154**	8.103	9.479	9.124
F8	AVG	817.544	819.999	817.578	818.407	818.971	**816.914**
	STD	**6.046**	7.181	7.332	6.505	8.377	7.283
F9	AVG	900.294	900.601	900.139	900.200	**900.118**	900.124
	STD	0.666	1.406	0.309	0.456	0.414	**0.299**
F10	AVG	1666.399	1755.451	1794.644	**1650.378**	1740.848	1813.138
	STD	276.410	285.118	340.923	**246.766**	260.343	252.345
F11	AVG	1111.970	1111.133	1112.685	1113.263	1111.381	**1109.898**
	STD	9.574	**4.175**	8.896	13.237	8.817	5.777
F12	AVG	1.282E + 04	1.194E + 04	1.375E + 04	1.232E + 04	**1.105E + 04**	1.380E + 04
	STD	9.639E + 03	**6.926E + 03**	9.751E + 03	8.122E + 03	7.525E + 03	1.056E + 04
F13	AVG	7.712E + 03	8.061E + 03	6.958E + 03	**6.609E + 03**	8.483E + 03	8.358E + 03
	STD	5243.024	4976.950	4289.342	**4264.100**	4969.781	5094.372
F14	AVG	1459.933	1455.562	**1451.157**	1461.718	1454.935	1462.153
	STD	33.376	24.621	**21.114**	42.699	27.959	31.612
F15	AVG	1560.125	1548.950	**1548.520**	1563.955	1550.480	1562.455
	STD	70.473	45.246	65.323	50.719	**43.575**	63.181
F16	AVG	1722.224	**1719.137**	1756.630	1732.078	1737.260	1784.496
	STD	125.121	**110.412**	150.588	129.688	132.981	129.439
F17	AVG	1746.013	1741.960	1742.751	1744.755	1739.671	**1739.451**
	STD	22.347	28.214	21.858	27.654	32.271	**21.117**
F18	AVG	1.249E + 04	1.133E + 04	**8.910E + 03**	1.145E + 04	9.333E + 03	1.144E + 04
	STD	1.152E + 04	1.079E + 04	**7.141E + 03**	8.989E + 03	8.770E + 03	1.114E + 04
F19	AVG	5346.020	5248.418	5811.505	**3830.378**	4433.477	4298.046
	STD	3601.822	3610.759	4518.454	2736.605	**2696.526**	3730.923
F20	AVG	2065.215	2071.315	**2048.261**	2064.562	2059.317	2061.875
	STD	59.808	62.194	**49.510**	50.540	52.295	53.796
F21	AVG	2301.552	**2287.715**	2313.057	2308.170	2295.181	2292.825
	STD	41.235	53.729	**31.756**	37.045	48.986	52.003
F22	AVG	2298.636	2301.316	2300.009	2301.501	2299.058	**2298.536**
	STD	15.775	**0.656**	7.794	0.820	14.394	15.312
F23	AVG	2615.773	2616.088	2614.798	**2614.022**	2615.625	2615.681
	STD	7.933	**5.240**	6.456	6.533	7.330	7.984
F24	AVG	2747.400	2729.996	2737.983	**2714.093**	2730.498	2737.914
	STD	**6.744**	62.883	45.471	85.723	63.092	45.601
F25	AVG	2929.513	2929.746	2932.090	2926.550	2933.128	**2925.301**
	STD	22.162	22.167	27.802	23.242	**21.118**	23.640
F26	AVG	3012.865	2901.459	**2897.367**	2898.257	2897.375	2918.368
	STD	337.581	224.031	78.903	101.261	**68.794**	215.475
F27	AVG	3096.576	3096.283	3096.307	3096.735	**3095.849**	3097.025
	STD	18.560	18.969	15.207	15.232	**13.224**	17.906
F28	AVG	**3308.714**	3349.607	3342.790	3344.003	3317.424	3342.396
	STD	183.521	119.064	**111.080**	113.920	128.693	147.237
F29	AVG	3214.929	3209.557	3207.533	3206.326	**3203.550**	3211.961
	STD	44.248	**40.942**	58.281	49.514	46.002	53.084
F30	AVG	3.850E + 05	1.553E + 05	1.219E + 05	**1.089E + 05**	2.338E + 05	1.777E + 05
	STD	5.725E + 05	3.987E + 05	**2.987E + 05**	3.061E + 05	4.299E + 05	4.219E + 05
FR rank		4.32	3.58	**2.99**	3.46	3.11	3.55

### CEC2017 results analysis

The CEC2017 benchmark suite consists of 29 benchmark test functions, each specifically tailored to fulfill certain objectives within its class. F1 and F3 are functions that have a single peak, making optimization straightforward. Functions F4 to F10 exhibit several modes with numerous peaks and valleys. Functions F11 to F20 are composite, incorporating a variety of landscapes, which adds complexity to the optimization problem. Functions F21 to F30 are composed of multiple sub-components, which collectively produce intricate optimization landscapes. The experimental validity of F2 has been compromised by uncontrollable factors, rendering it unsuitable for experimentation. Therefore, we refrained from doing tests on F2. The next part presents a comprehensive analysis of the test findings derived from the experiments conducted on these functions.

#### CEC2017 statistical performance

Tables 6 and 7 present the empirical results for the situations with sizes of 50 and 100. Tables 6 and 7 depict the mean, ranking, and standard deviation of objective function values for each algorithm. The AD-COA-L algorithm has demonstrated outstanding performance in locating the global optimum, particularly in experiments involving the single-peaked issue F1.

During the trials conducted in a 50-dimensional space, AD-COA-L initially demonstrates a minor advantage over INFO in terms of F1 performance. Nevertheless, it quickly exceeds the original state and progresses towards the optimal solution. Nevertheless, in the case of trials done in a 100-dimensional space, AD-COA-L continuously surpassed INFO in terms of performance on the F1 function, retaining a persistent advantage throughout. The INFO algorithm demonstrated the highest average value in problems with a dimension of 50 in the example of F3. Nevertheless, AD-COA-L exhibited superior performance compared to all other algorithms when applied to the tested functions in a 100-dimensional space. The expanded power of AD-COA-L to discover and converge towards the most optimal solutions for problems with a single highest point is proven, confirming its robust potential to both explore and exploit global optima.

AD-COA-L exhibits superior performance in the majority of functions for multimodal problems F4-F10 when compared to the other eleven comparison algorithms. When comparing the AD-COA-L algorithm to the INFO, PSO, and WSO algorithms, it becomes apparent that the AD-COA-L approach demonstrates a slower convergence and achieves inferior results in the 50-dimensional trials. However, it shows a comparatively lower level of performance on function F6. Within the function F7, the efficiency of the INFO and PSO methods surpasses that of AD-COA-L. Nevertheless, the disparity between AD-COA-L and these algorithms is minimal, indicating that AD-COA-L exhibits commendable performance in F7. However, AD-COA-L has lower performance than INFO on functions F5, F6, and F8. Nevertheless, AD-COA-L demonstrates exceptional performance in the remaining functions, proving its supremacy and resilience in effectively resolving complex challenges. AD-COA-L demonstrates excellent competence in effectively managing a diverse variety of mixed functions, ranging from F11 to F20. When tested in a 50-dimensional configuration, the performance of AD-COA-L is similar to that of INFO on F11, but slightly worse on F12, F14, and F18. However, the standard deviation of AD-COA-L at F18 exceeds that of INFO, suggesting that AD-COA-L demonstrates more stability in this function. AD-COA-L outperforms other algorithms in most functions when considering 100 dimensions, except for F12 and F13, which are effectively handled by INFO. Significantly, there is a slight discrepancy in the performance of AD-COA-L and INFO at F12 and F13, with AD-COA-L exhibiting superior stability compared to INFO at F12. The outstanding success of AD-COA-L can be credited to its wide array of solution search strategies, namely its immensely powerful global search capabilities, which is remarkably effective in addressing intricate problems.

AD-COA-L is capable of effectively resolving complex problems, namely those related to functions F21-F30. In a study involving 50 dimensions, the AD-COA-L algorithm has outstanding performance, outperforming all others except for F21, F27, and F30, which have higher rankings. AD-COA-L demonstrates superior performance compared to all other algorithms across all functions, with the exception of the INFO function in F26, F29, and F30, while testing with 100-dimensional data. The results clearly demonstrate that AD-COA-L is exceptional and highly versatile in efficiently addressing a wide range of challenges. It enables a thorough examination and enhancement of intricate search domains.

**Table 6:** Comparative analysis between AD-COA-L and its rivals using CEC2017, D = 50.

F		AD-COA-L	COA	HHO	WSO	AOA	WOA	PSO	INFO	SWO	SMA	SCA	GBO
F1	AVG	**1.55** **L** **EL + 04**	1.25LEL + 09	4.01LEL + 10	2.81LEL + 10	1.11LEL + 11	8.18LEL + 09	5.29LEL + 08	2.09LEL + 06	6.81LEL + 10	4.09LEL + 10	5.98LEL + 10	6.77LEL + 9
	STD	1.07LEL + 04	8.50LEL + 08	1.04LEL + 10	7.27LEL + 09	1.11LEL + 10	2.84LEL + 09	5.49LEL + 07	**1.95LEL + 06**	1.03LEL + 10	1.97LEL + 10	9.55LEL + 09	2.67LEL + 9
F3	AVG	1.31LEL + 05	2.33LEL + 05	1.39LEL + 05	1.16LEL + 05	1.69LEL + 05	2.68LEL + 05	1.15LEL + 05	**4.88LEL + 04**	2.28LEL + 05	3.84LEL + 05	1.79LEL + 05	3.25LEL + 5
	STD	**1.96LEL + 04**	4.38LEL + 04	1.32LEL + 04	1.78LEL + 04	2.04LEL + 04	7.62LEL + 04	2.39LEL + 04	1.05LEL + 04	5.38LEL + 04	9.74LEL + 04	2.55LEL + 04	4.38LEL + 4
F4	AVG	**5.29LEL + 02**	9.67LEL + 02	4.85LEL + 03	4.48LEL + 03	3.42LEL + 04	2.75LEL + 03	6.32LEL + 02	5.47LEL + 02	1.48LEL + 04	5.22LEL + 03	1.13LEL + 04	2.22LEL + 03
	STD	**5.35LEL + 01**	2.23LEL + 02	1.59LEL + 03	1.54LEL + 03	6.39LEL + 03	8.16LEL + 02	7.69LEL + 01	5.89LEL + 01	3.69LEL + 03	3.06LEL + 03	2.45LEL + 03	1.12LEL + 03
F5	AVG	**7.90LEL + 02**	8.98LEL + 02	9.54LEL + 02	8.09LEL + 02	1.16LEL + 03	1.07LEL + 03	1.00LEL + 03	8.13LEL + 02	1.14LEL + 03	9.87LEL + 02	1.12LEL + 03	8.67LEL + 02
	STD	3.21LEL + 01	9.10LEL + 01	4.69LEL + 01	4.57LEL + 01	3.71LEL + 01	9.64LEL + 01	4.35LEL + 01	**5.07LEL + 01**	4.75LEL + 01	8.98LEL + 01	3.02LEL + 01	3.37LEL + 01
F6	AVG	6.45LEL + 02	6.67LEL + 02	6.71LEL + 02	6.56LEL + 02	6.95LEL + 02	6.94LEL + 02	6.82LEL + 02	**6.43LEL + 02**	6.84LEL + 02	6.62LEL + 02	6.82LEL + 02	6.69LEL + 02
	STD	9.96LEL + 00	1.19LEL + 01	7.92LEL + 00	8.71LEL + 00	9.45LEL + 00	1.05LEL + 01	5.95LEL + 00	**4.99LEL + 00**	8.90LEL + 00	1.12LEL + 01	5.16LEL + 00	7.13 + LEL00
F7	AVG	1.26LEL + 03	1.37LEL + 03	1.60LEL + 03	1.60LEL + 03	1.97LEL + 03	1.87LEL + 03	1.24LEL + 03	**1.22LEL + 03**	1.93LEL + 03	2.14LEL + 03	1.81LEL + 03	1.76LEL + 03
	STD	**1.14LEL + 02**	9.85LEL + 01	7.96LEL + 01	1.02LEL + 02	5.97LEL + 01	8.43LEL + 01	4.05LEL + 01	1.02LEL + 02	1.08LEL + 02	4.21LEL + 02	1.04LEL + 02	6.67LEL + 01
F8	AVG	**1.10LEL + 03**	1.30LEL + 03	1.26LEL + 03	1.12LEL + 03	1.50LEL + 03	1.33LEL + 03	1.31LEL + 03	1.10LEL + 03	1.44LEL + 03	1.29LEL + 03	1.44LEL + 03	1.19LEL + 03
	STD	**4.39LEL + 01**	9.66LEL + 01	5.36LEL + 01	4.42LEL + 01	4.16LEL + 01	7.10LEL + 01	5.19LEL + 01	3.71LEL + 01	5.26LEL + 01	9.93LEL + 01	3.50LEL + 01	3.21 + LEL01
F9	AVG	**1.10LEL + 04**	2.20LEL + 04	2.08LEL + 04	2.81LEL + 04	2.99LEL + 04	3.44LEL + 04	2.87LEL + 04	1.12LEL + 04	3.84LEL + 04	2.01LEL + 04	2.88LEL + 04	1.69LEL + 04
	STD	**2.29LEL + 03**	6.81LEL + 03	3.59LEL + 03	4.21LEL + 03	3.24LEL + 03	1.05LEL + 04	5.48LEL + 03	5.40LEL + 03	4.87LEL + 03	4.88LEL + 03	4.26LEL + 03	4.62LEL + 03
F10	AVG	**7.88LEL + 03**	1.03LEL + 04	1.24LEL + 04	8.49LEL + 03	1.38LEL + 04	1.27LEL + 04	1.20LEL + 04	8.64LEL + 03	1.54LEL + 04	8.95LEL + 03	1.53LEL + 04	1.08LEL + 04
	STD	**2.03LEL + 03**	2.11LEL + 03	9.97LEL + 02	2.31LEL + 03	7.70LEL + 02	1.17LEL + 03	8.42LEL + 02	1.27LEL + 03	6.24LEL + 02	9.03LEL + 02	4.57LEL + 02	1.48LEL + 03
F11	AVG	**1.32LEL + 03**	3.18LEL + 03	8.00LEL + 03	4.95LEL + 03	2.34LEL + 04	5.33LEL + 03	1.63LEL + 03	1.37LEL + 03	1.80LEL + 04	1.55LEL + 04	1.05LEL + 04	4.38LEL + 03
	STD	5.23LEL + 01	2.18LEL + 03	2.82LEL + 03	2.36LEL + 03	3.29LEL + 03	1.68LEL + 03	6.18LEL + 01	**7.48LEL + 01**	4.94LEL + 03	1.32LEL + 04	2.27LEL + 03	1.50LEL + 03
F12	AVG	1.52LEL + 07	4.74LEL + 08	6.00LEL + 09	4.72LEL + 09	7.35LEL + 10	1.58LEL + 09	3.15LEL + 08	**9.58LEL + 06**	1.65LEL + 10	7.07LEL + 09	1.91LEL + 10	2.56LEL + 09
	STD	**5.77LEL + 06**	3.80LEL + 08	3.03LEL + 09	3.71LEL + 09	1.41LEL + 10	6.99LEL + 08	9.77LEL + 07	5.44LEL + 06	4.99LEL + 09	5.73LEL + 09	4.79LEL + 09	4.98LEL + 09
F13	AVG	**9.97LEL + 03**	5.25LEL + 07	1.08LEL + 09	2.59LEL + 08	3.95LEL + 10	1.34LEL + 08	4.99LEL + 07	8.16LEL + 04	5.82LEL + 09	1.13LEL + 09	5.47LEL + 09	4.39LEL + 08
	STD	6.96LEL + 03	8.61LEL + 07	1.44LEL + 09	3.97LEL + 08	1.12LEL + 10	9.72LEL + 07	9.86LEL + 06	**5.54LEL + 04**	2.50LEL + 09	2.06LEL + 09	2.20LEL + 09	2.00LEL + 09
F14	AVG	2.91LEL + 05	3.45LEL + 06	1.80LEL + 06	9.95LEL + 05	8.80LEL + 07	4.72LEL + 06	3.04LEL + 05	**2.36LEL + 05**	9.66LEL + 06	5.15LEL + 06	5.15LEL + 06	1.05 + LEL07
	STD	**1.65LEL + 05**	3.39LEL + 06	1.51LEL + 06	1.07LEL + 06	6.20LEL + 07	4.09LEL + 06	1.29LEL + 05	1.42LEL + 05	5.82LEL + 06	1.04LEL + 07	2.51LEL + 06	9.02LEL + 06
F15	AVG	**1.53LEL + 04**	3.56LEL + 07	2.41LEL + 08	3.43LEL + 07	6.04LEL + 09	1.29LEL + 07	1.32LEL + 07	2.37LEL + 04	1.05LEL + 09	1.46LEL + 08	8.71LEL + 08	1.03LEL + 08
	STD	**5.57LEL + 03**	1.19LEL + 08	4.80LEL + 08	1.19LEL + 08	3.17LEL + 09	1.73LEL + 07	3.48LEL + 06	1.01LEL + 04	6.73LEL + 08	4.24LEL + 08	3.35LEL + 08	3.87LEL08
F16	AVG	**3.36LEL + 03**	4.68LEL + 03	4.44LEL + 03	3.50LEL + 03	8.40LEL + 03	6.15LEL + 03	4.32LEL + 03	3.84LEL + 03	6.46LEL + 03	4.13LEL + 03	6.20LEL + 03	5.44LEL + 03
	STD	4.03LEL + 02	6.53LEL + 02	5.35LEL + 02	4.50LEL + 02	1.78LEL + 03	8.16LEL + 02	4.27LEL + 02	**5.62LEL + 02**	5.09LEL + 02	4.64LEL + 02	4.10LEL + 02	1.00LEL + 03
F17	AVG	3.28LEL + 03	4.20LEL + 03	3.89LEL + 03	3.07LEL + 03	1.16LEL + 04	4.23LEL + 03	3.59LEL + 03	**3.05LEL + 03**	4.90LEL + 03	4.18LEL + 03	4.78LEL + 03	4.00 + LEL03
	STD	3.36LEL + 02	4.05LEL + 02	4.02LEL + 02	2.34LEL + 02	4.67LEL + 03	5.45LEL + 02	3.80LEL + 02	**3.88LEL + 02**	4.90LEL + 02	4.55LEL + 02	3.72LEL + 02	4.02LEL + 02
F18	AVG	2.09LEL + 06	8.30LEL + 06	1.00LEL + 07	2.13LEL + 06	1.49LEL + 08	3.41LEL + 07	2.59LEL + 06	**1.50LEL + 06**	4.42LEL + 07	1.74LEL + 07	4.32LEL + 07	3.31LEL + 07
	STD	**4.80LEL + 05**	7.47LEL + 06	8.99LEL + 06	1.97LEL + 06	1.30LEL + 08	2.38LEL + 07	1.35LEL + 06	1.81LEL + 06	2.52LEL + 07	2.99LEL + 07	2.40LEL + 07	4.49LEL + 07
F19	AVG	**2.41LEL + 04**	5.55LEL + 06	1.39LEL + 08	1.58LEL + 05	4.16LEL + 09	9.13LEL + 06	9.35LEL + 06	3.20LEL + 04	2.71LEL + 08	2.24LEL + 08	4.94LEL + 08	5.57LEL + 07
	STD	1.29LEL + 04	6.98LEL + 06	2.88LEL + 08	**4.44LEL + 05**	2.05LEL + 09	1.09LEL + 07	4.08LEL + 06	1.46LEL + 04	1.58LEL + 08	4.78LEL + 08	2.66LEL + 08	2.60LEL + 08
F20	AVG	3.05LEL + 03	3.75LEL + 03	3.82LEL + 03	**2.91LEL + 03**	3.71LEL + 03	3.82LEL + 03	3.59LEL + 03	3.09LEL + 03	4.50LEL + 03	3.67LEL + 03	4.19LEL + 03	3.77LEL + 03
	STD	2.38LEL + 02	3.68LEL + 02	4.44LEL + 02	4.31LEL + 02	3.08LEL + 02	2.74LEL + 02	2.72LEL + 02	**3.98LEL + 02**	2.42LEL + 02	3.09LEL + 02	1.52LEL + 02	3.20LEL + 02
F21	AVG	2.61LEL + 03	2.87LEL + 03	2.76LEL + 03	2.70LEL + 03	3.11LEL + 03	3.04LEL + 03	2.86LEL + 03	**2.54LEL + 03**	2.96LEL + 03	2.77LEL + 03	2.94LEL + 03	2.90LEL + 03
	STD	**4.62LEL + 01**	8.69LEL + 01	5.24LEL + 01	7.28LEL + 01	8.43LEL + 01	1.26LEL + 02	6.78LEL + 01	5.18LEL + 01	4.63LEL + 01	6.54LEL + 01	4.13LEL + 01	1.05LEL + 02
F22	AVG	**9.37LEL + 03**	1.23LEL + 04	1.45LEL + 04	1.03LEL + 04	1.60LEL + 04	1.43LEL + 04	1.36LEL + 04	9.61LEL + 03	1.67LEL + 04	1.11LEL + 04	1.70LEL + 04	1.30LEL + 04
	STD	3.42LEL + 02	2.26LEL + 03	1.18LEL + 03	2.00LEL + 03	5.83LEL + 02	9.37LEL + 02	7.89LEL + 02	**1.72LEL + 03**	2.02LEL + 03	1.17LEL + 03	3.00LEL + 03	1.54LEL + 03
F23	AVG	3.30LEL + 03	3.50LEL + 03	3.27LEL + 03	3.73LEL + 03	4.47LEL + 03	3.74LEL + 03	3.81LEL + 03	**3.01LEL + 03**	3.77LEL + 03	3.21LEL + 03	3.66LEL + 03	4.01LEL + 03
	STD	**9.20LEL + 01**	1.64LEL + 02	7.43LEL + 01	1.56LEL + 02	2.29LEL + 02	1.72LEL + 02	3.30LEL + 02	6.48LEL + 01	1.03LEL + 02	6.01LEL + 01	6.45LEL + 01	1.92LEL + 02
F24	AVG	**3.13LEL + 03**	3.66LEL + 03	3.35LEL + 03	4.21LEL + 03	5.01LEL + 03	3.86LEL + 03	3.80LEL + 03	3.47LEL + 03	4.00LEL + 03	3.25LEL + 03	3.83LEL + 03	4.42LEL + 03
	STD	**4.87LEL + 01**	1.38LEL + 02	7.47LEL + 01	2.09LEL + 02	3.37LEL + 02	1.48LEL + 02	1.85LEL + 02	1.50LEL + 02	1.01LEL + 02	6.40LEL + 01	6.52LEL + 01	1.77LEL + 02
F25	AVG	**3.07LEL + 03**	3.78LEL + 03	5.96LEL + 03	5.29LEL + 03	1.56LEL + 04	4.22LEL + 03	3.08LEL + 03	3.10LEL + 03	1.06LEL + 04	5.12LEL + 03	8.28LEL + 03	3.88LEL + 03
	STD	**2.23LEL + 01**	1.82LEL + 03	9.26LEL + 02	7.91LEL + 02	1.63LEL + 03	3.37LEL + 02	5.25LEL + 01	2.69LEL + 01	1.39LEL + 03	1.55LEL + 03	1.03LEL + 03	3.24LEL + 02
F26	AVG	**7.10LEL + 03**	1.02LEL + 04	8.99LEL + 03	1.11LEL + 04	1.69LEL + 04	1.45LEL + 04	9.50LEL + 03	9.50LEL + 03	1.44LEL + 04	8.79LEL + 03	1.35LEL + 04	1.17LEL + 04
	STD	2.62LEL + 03	2.10LEL + 03	4.18LEL + 02	2.09LEL + 03	9.95LEL + 02	1.84LEL + 03	3.63LEL + 03	**2.22LEL + 03**	9.99LEL + 02	6.92LEL + 02	6.92LEL + 02	2.22LEL + 03
F27	AVG	3.56LEL + 03	4.05LEL + 03	3.88LEL + 03	4.57LEL + 03	6.98LEL + 03	4.64LEL + 03	3.69LEL + 03	**3.36LEL + 03**	5.33LEL + 03	3.65LEL + 03	4.81LEL + 03	5.06LEL + 03
	STD	**1.19LEL + 02**	2.92LEL + 02	1.63LEL + 02	2.83LEL + 02	7.12LEL + 02	5.08LEL + 02	5.56LEL + 02	1.04LEL + 02	3.51LEL + 02	1.69LEL + 02	2.68LEL + 02	1.00LEL + 03
F28	AVG	**3.32LEL + 03**	5.40LEL + 03	8.74LEL + 03	5.54LEL + 03	1.26LEL + 04	5.06LEL + 03	3.34LEL + 03	3.33LEL + 03	8.65LEL + 03	7.74LEL + 03	8.19LEL + 03	5.43LEL + 03
	STD	**2.33LEL + 01**	2.27LEL + 03	1.93LEL + 03	5.36LEL + 02	1.53LEL + 03	4.32LEL + 02	4.21LEL + 01	3.75LEL + 01	6.76LEL + 02	1.75LEL + 03	9.42LEL + 02	1.18LEL + 03
F29	AVG	**4.61LEL + 03**	5.95LEL + 03	7.11LEL + 03	5.66LEL + 03	6.67LEL + 04	8.65LEL + 03	5.99LEL + 03	5.02LEL + 03	9.15LEL + 03	5.73LEL + 03	8.04LEL + 03	1.19LEL + 04
	STD	4.08LEL + 02	8.79LEL + 02	1.31LEL + 03	4.71LEL + 02	1.30LEL + 05	1.50LEL + 03	5.94LEL + 02	**4.20LEL + 02**	8.87LEL + 02	6.52LEL + 02	7.31LEL + 02	5.04LEL + 03
F30	AVG	3.01LEL + 06	3.31LEL + 07	2.89LEL + 08	4.46LEL + 07	5.78LEL + 09	2.88LEL + 08	1.30LEL + 08	**2.35LEL + 06**	7.60LEL + 08	3.06LEL + 08	1.02LEL + 09	2.18LEL + 08
	STD	**1.38LEL + 06**	2.46LEL + 07	1.77LEL + 08	2.08LEL + 07	3.43LEL + 09	1.14LEL + 08	2.21LEL + 07	8.05LEL + 05	6.03LEL + 08	6.01LEL + 08	2.54LEL + 08	1.97LEL + 08
	FR rank	2.28	6.46	6.74	5.44	10.10	7.72	4.92	3.15	9.25	7.44	7.62	7.31
	Final rank	**1**	5	6	4	12	10	3	2	11	8	9	7

**Table 7:** Comparative analysis between AD-COA-L and its rivals using CEC2017, D = 100.

F		AD-COA-L	COA	HHO	WSO	AOA	WOA	PSO	INFO	SWO	SMA	SCA	GBO
F1	AVG	**8.09LEL + 06**	6.68LEL + 10	1.50LEL + 11	1.30LEL + 11	2.67LEL + 11	6.63LEL + 10	2.08LEL + 09	2.03LEL + 07	2.18LEL + 11	1.31LEL + 11	1.99LEL + 11	4.73LEL + 10
	STD	**3.18LEL + 06**	6.98LEL + 10	1.13LEL + 10	1.55LEL + 10	1.22LEL + 10	6.88LEL + 09	1.67LEL + 08	1.06LEL + 07	1.64LEL + 10	4.06LEL + 10	1.30LEL + 10	8.41LEL + 09
F3	AVG	**2.73LEL + 05**	4.84LEL + 05	4.74LEL + 05	3.07LEL + 05	3.52LEL + 05	9.16LEL + 05	4.54LEL + 05	3.43LEL + 05	4.90LEL + 05	9.77LEL + 05	5.08LEL + 05	7.46LEL + 05
	STD	2.21LEL + 04	1.83LEL + 05	1.13LEL + 05	3.90LEL + 04	**1.68LEL + 04**	9.73LEL + 04	6.42LEL + 04	2.07LEL + 04	6.41LEL + 04	1.96LEL + 05	6.96LEL + 04	6.29LEL + 04
F4	AVG	**8.24LEL + 02**	7.70LEL + 03	2.18LEL + 04	1.95LEL + 04	9.03LEL + 04	1.15LEL + 04	9.87LEL + 02	8.56LEL + 02	4.97LEL + 04	2.49LEL + 04	4.50LEL + 04	1.02LEL + 04
	STD	7.43LEL + 01	9.97LEL + 03	4.43LEL + 03	4.14LEL + 03	2.14LEL + 04	2.23LEL + 03	1.30LEL + 02	**6.44LEL + 01**	9.33LEL + 03	1.57LEL + 04	6.74LEL + 03	2.24LEL + 03
F5	AVG	1.27LEL + 03	1.59LEL + 03	1.68LEL + 03	1.39LEL + 03	2.06LEL + 03	1.85LEL + 03	1.77LEL + 03	**1.27LEL + 03**	2.05LEL + 03	1.88LEL + 03	2.02LEL + 03	1.46LEL + 03
	STD	**5.93LEL + 01**	2.44LEL + 02	6.36LEL + 01	7.32LEL + 01	7.57LEL + 01	1.19LEL + 02	8.37LEL + 01	8.36LEL + 01	7.83LEL + 01	1.60LEL + 02	6.14LEL + 01	7.35LEL + 01
F6	AVG	6.58LEL + 02	6.78LEL + 02	6.86LEL + 02	6.71LEL + 02	7.09LEL + 02	7.03LEL + 02	6.97LEL + 02	**6.55LEL + 02**	7.07LEL + 02	6.78LEL + 02	7.01LEL + 02	6.76LEL + 02
	STD	**4.35LEL + 00**	1.26LEL + 01	4.65LEL + 00	8.76LEL + 00	5.99LEL + 00	1.20LEL + 01	5.52LEL + 00	5.71LEL + 00	6.26LEL + 00	7.94LEL + 00	4.45LEL + 00	5.32LEL + 00
F7	AVG	2.58LEL + 03	2.84LEL + 03	3.30LEL + 03	3.31LEL + 03	3.94LEL + 03	3.74LEL + 03	**2.03LEL + 03**	2.46LEL + 03	3.88LEL + 03	5.52LEL + 03	3.87LEL + 03	3.46LEL + 03
	STD	1.86LEL + 02	1.89LEL + 02	1.37LEL + 02	2.40LEL + 02	**8.12LEL + 01**	1.58LEL + 02	1.25LEL + 02	2.63LEL + 02	1.24LEL + 02	8.41LEL + 02	1.77LEL + 02	1.29LEL + 02
F8	AVG	1.67LEL + 03	2.08LEL + 03	2.04LEL + 03	1.82LEL + 03	2.50LEL + 03	2.33LEL + 03	2.16LEL + 03	**1.60LEL + 03**	2.47LEL + 03	2.19LEL + 03	2.36LEL + 03	1.89LEL + 03
	STD	9.30LEL + 01	2.19LEL + 02	7.86LEL + 01	8.56LEL + 01	6.93LEL + 01	1.56LEL + 02	7.52LEL + 01	8.96LEL + 01	8.52LEL + 01	1.63LEL + 02	**6.59LEL + 01**	6.64LEL + 01
F9	AVG	**2.57LEL + 04**	6.80LEL + 04	5.96LEL + 04	7.37LEL + 04	6.84LEL + 04	7.88LEL + 04	7.21LEL + 04	4.70LEL + 04	8.89LEL + 04	5.20LEL + 04	8.43LEL + 04	3.92LEL + 04
	STD	**1.52LEL + 03**	1.27LEL + 04	7.23LEL + 03	6.65LEL + 03	5.65LEL + 03	1.69LEL + 04	8.37LEL + 03	1.18LEL + 04	6.15LEL + 03	6.51LEL + 03	8.56LEL + 03	4.01LEL + 03
F10	AVG	**1.58LEL + 04**	2.52LEL + 04	2.84LEL + 04	2.15LEL + 04	3.09LEL + 04	2.78LEL + 04	2.71LEL + 04	2.15LEL + 04	3.32LEL + 04	1.85LEL + 04	3.26LEL + 04	2.53LEL + 04
	STD	1.74LEL + 03	5.73LEL + 03	1.82LEL + 03	4.66LEL + 03	1.16LEL + 03	1.55LEL + 03	1.41LEL + 03	4.75LEL + 03	7.48LEL + 02	1.47LEL + 03	**5.52LEL + 02**	3.06LEL + 03
F11	AVG	**3.60LEL + 03**	1.72LEL + 05	1.02LEL + 05	7.32LEL + 04	1.67LEL + 05	1.97LEL + 05	1.31LEL + 04	3.26LEL + 04	2.00LEL + 05	2.25LEL + 05	1.50LEL + 05	3.18LEL + 05
	STD	**9.91LEL + 02**	3.87LEL + 04	1.84LEL + 04	1.70LEL + 04	2.43LEL + 04	7.59LEL + 04	4.74LEL + 03	1.03LEL + 04	4.01LEL + 04	1.13LEL + 05	2.12LEL + 04	5.43LEL + 04
F12	AVG	1.04LEL + 08	3.04LEL + 09	4.88LEL + 10	4.64LEL + 10	1.87LEL + 11	1.39LEL + 10	1.44LEL + 09	**9.48LEL + 07**	9.90LEL + 10	4.69LEL + 10	8.87LEL + 10	1.87LEL + 10
	STD	**3.56LEL + 07**	9.26LEL + 08	1.37LEL + 10	1.67LEL + 10	2.59LEL + 10	4.69LEL + 09	2.76LEL + 08	5.57LEL + 07	1.66LEL + 10	2.30LEL + 10	1.15LEL + 10	6.71LEL + 09
F13	AVG	1.93LEL + 05	1.57LEL + 08	7.22LEL + 09	5.87LEL + 09	4.87LEL + 10	6.92LEL + 08	1.20LEL + 08	**1.05LEL + 05**	1.59LEL + 10	7.02LEL + 09	1.53LEL + 10	4.58LEL + 08
	STD	6.01LEL + 05	1.55LEL + 08	3.24LEL + 09	2.66LEL + 09	6.15LEL + 09	3.71LEL + 08	1.51LEL + 07	**5.73LEL + 04**	3.39LEL + 09	4.35LEL + 09	2.58LEL + 09	3.77LEL + 08
F14	AVG	**7.52LEL + 05**	1.09LEL + 07	1.17LEL + 07	6.74LEL + 06	1.18LEL + 08	1.41LEL + 07	4.35LEL + 06	2.52LEL + 06	3.50LEL + 07	1.67LEL + 07	4.74LEL + 07	1.97LEL + 07
	STD	**2.14LEL + 05**	8.14LEL + 06	5.52LEL + 06	3.34LEL + 06	9.66LEL + 07	7.20LEL + 06	1.76LEL + 06	2.49LEL + 06	1.27LEL + 07	2.52LEL + 07	2.08LEL + 07	1.04LEL + 07
F15	AVG	**1.67LEL + 04**	3.22LEL + 07	2.19LEL + 09	1.54LEL + 09	2.51LEL + 10	1.03LEL + 08	4.69LEL + 07	4.45LEL + 04	4.71LEL + 09	1.35LEL + 09	5.19LEL + 09	8.32LEL + 07
	STD	4.11LEL + 04	6.52LEL + 07	1.62LEL + 09	1.99LEL + 09	5.11LEL + 09	9.29LEL + 07	6.79LEL + 06	**2.83LEL + 04**	1.90LEL + 09	1.24LEL + 09	1.41LEL + 09	3.26LEL + 08
F16	AVG	**6.14LEL + 03**	8.74LEL + 03	9.44LEL + 03	7.77LEL + 03	2.10LEL + 04	1.53LEL + 04	9.14LEL + 03	6.69LEL + 03	1.48LEL + 04	8.32LEL + 03	1.43LEL + 04	9.97LEL + 03
	STD	**7.25LEL + 02**	1.33LEL + 03	8.62LEL + 02	9.67LEL + 02	3.46LEL + 03	2.43LEL + 03	9.98LEL + 02	8.82LEL + 02	1.32LEL + 03	1.15LEL + 03	8.16LEL + 02	1.82LEL + 03
F17	AVG	**5.45LEL + 03**	8.21LEL + 03	8.80LEL + 03	1.53LEL + 04	6.98LEL + 06	1.37LEL + 04	6.74LEL + 03	6.44LEL + 03	6.01LEL + 04	1.74LEL + 04	4.56LEL + 04	9.35LEL + 03
	STD	**4.92LEL + 02**	1.15LEL + 03	2.43LEL + 03	2.28LEL + 04	5.39LEL + 06	5.46LEL + 03	5.80LEL + 02	8.01LEL + 02	7.01LEL + 04	1.73LEL + 04	4.35LEL + 04	2.92LEL + 03
F18	AVG	**1.46LEL + 06**	2.75LEL + 07	1.53LEL + 07	6.13LEL + 06	1.75LEL + 08	1.25LEL + 07	5.41LEL + 06	3.63LEL + 06	5.65LEL + 07	2.70LEL + 07	9.46LEL + 07	1.75LEL + 07
	STD	**4.83LEL + 05**	1.95LEL + 07	8.70LEL + 06	4.43LEL + 06	1.10LEL + 08	5.95LEL + 06	2.09LEL + 06	2.43LEL + 06	2.70LEL + 07	2.89LEL + 07	4.28LEL + 07	2.00LEL + 07
F19	AVG	**1.74LEL + 04**	4.22LEL + 07	1.83LEL + 09	8.98LEL + 08	2.40LEL + 10	1.07LEL + 08	6.38LEL + 07	2.54LEL + 04	5.08LEL + 09	1.14LEL + 09	4.77LEL + 09	6.03LEL + 07
	STD	**1.32LEL + 04**	4.01LEL + 07	1.01LEL + 09	1.12LEL + 09	5.59LEL + 09	4.25LEL + 07	1.35LEL + 07	2.26LEL + 04	1.72LEL + 09	1.44LEL + 09	1.49LEL + 09	4.01LEL + 07
F20	AVG	**5.16LEL + 03**	6.99LEL + 03	6.76LEL + 03	5.43LEL + 03	7.28LEL + 03	7.04LEL + 03	6.59LEL + 03	5.41LEL + 03	8.19LEL + 03	5.89LEL + 03	7.86LEL + 03	6.57LEL + 03
	STD	4.00LEL + 02	7.08LEL + 02	8.77LEL + 02	9.69LEL + 02	3.65LEL + 02	5.73LEL + 02	4.26LEL + 02	6.92LEL + 02	4.46LEL + 02	8.12LEL + 02	**2.93LEL + 02**	6.44LEL + 02
F21	AVG	**2.96LEL + 03**	3.97LEL + 03	3.65LEL + 03	3.86LEL + 03	4.72LEL + 03	4.29LEL + 03	3.99LEL + 03	3.15LEL + 03	4.08LEL + 03	3.70LEL + 03	4.12LEL + 03	4.39LEL + 03
	STD	**8.00LEL + 01**	1.88LEL + 02	1.07LEL + 02	1.34LEL + 02	2.07LEL + 02	2.05LEL + 02	1.40LEL + 02	1.32LEL + 02	1.07LEL + 02	1.47LEL + 02	1.04LEL + 02	2.43LEL + 02
F22	AVG	**2.07LEL + 04**	2.84LEL + 04	3.10LEL + 04	2.17LEL + 04	3.35LEL + 04	3.04LEL + 04	3.00LEL + 04	2.32LEL + 04	3.56LEL + 04	2.09LEL + 04	3.50LEL + 04	2.73LEL + 04
	STD	2.37LEL + 03	5.95LEL + 03	1.52LEL + 03	1.53LEL + 03	9.69LEL + 02	1.60LEL + 03	1.47LEL + 03	4.27LEL + 03	8.85LEL + 02	1.34LEL + 03	**5.73LEL + 02**	2.83LEL + 03
F23	AVG	**3.46LEL + 03**	4.78LEL + 03	4.16LEL + 03	5.03LEL + 03	7.31LEL + 03	5.18LEL + 03	5.16LEL + 03	4.03LEL + 03	5.53LEL + 03	3.87LEL + 03	5.13LEL + 03	6.05LEL + 03
	STD	**1.02LEL + 02**	1.94LEL + 02	1.22LEL + 02	3.54LEL + 02	4.61LEL + 02	2.62LEL + 02	3.81LEL + 02	2.40LEL + 02	2.40LEL + 02	1.21LEL + 02	1.07LEL + 02	3.15LEL + 02
F24	AVG	**4.14LEL + 03**	6.01LEL + 03	4.95LEL + 03	6.78LEL + 03	1.15LEL + 04	6.48LEL + 03	6.43LEL + 03	5.01LEL + 03	8.22LEL + 03	4.52LEL + 03	7.19LEL + 03	9.29LEL + 03
	STD	**1.06LEL + 02**	4.86LEL + 02	1.73LEL + 02	4.70LEL + 02	1.10LEL + 03	3.73LEL + 02	4.87LEL + 02	4.42LEL + 02	4.49LEL + 02	1.59LEL + 02	3.12LEL + 02	9.00LEL + 02
F25	AVG	**3.42LEL + 03**	8.24LEL + 03	1.41LEL + 04	1.18LEL + 04	2.93LEL + 04	8.10LEL + 03	3.60LEL + 03	3.51LEL + 03	2.21LEL + 04	1.47LEL + 04	2.05LEL + 04	6.58LEL + 03
	STD	5.96LEL + 01	6.21LEL + 03	1.33LEL + 03	1.63LEL + 03	2.56LEL + 03	7.95LEL + 02	**4.03LEL + 01**	4.96LEL + 01	1.56LEL + 03	6.06LEL + 03	2.67LEL + 03	6.00LEL + 02
F26	AVG	1.75LEL + 04	2.60LEL + 04	2.20LEL + 04	3.33LEL + 04	5.25LEL + 04	3.64LEL + 04	**1.70LEL + 04**	2.33LEL + 04	4.03LEL + 04	1.95LEL + 04	3.95LEL + 04	3.22LEL + 04
	STD	5.06LEL + 03	3.61LEL + 03	**1.51LEL + 03**	2.27LEL + 03	3.95LEL + 03	3.38LEL + 03	9.98LEL + 03	2.89LEL + 03	2.71LEL + 03	2.24LEL + 03	2.35LEL + 03	2.09LEL + 03
F27	AVG	3.79LEL + 03	4.65LEL + 03	4.71LEL + 03	6.77LEL + 03	1.39LEL + 04	5.82LEL + 03	**3.39LEL + 03**	3.78LEL + 03	8.67LEL + 03	4.02LEL + 03	8.24LEL + 03	8.78LEL + 03
	STD	1.37LEL + 02	4.66LEL + 02	3.28LEL + 02	8.37LEL + 02	1.43LEL + 03	8.03LEL + 02	**9.44LEL + 01**	2.15LEL + 02	5.72LEL + 02	2.56LEL + 02	5.41LEL + 02	2.55LEL + 03
F28	AVG	**3.54LEL + 03**	1.73LEL + 04	2.47LEL + 04	1.58LEL + 04	3.45LEL + 04	1.10LEL + 04	3.60LEL + 03	3.60LEL + 03	2.60LEL + 04	1.96LEL + 04	2.57LEL + 04	1.07LEL + 04
	STD	**4.29LEL + 01**	7.02LEL + 03	5.26LEL + 03	1.87LEL + 03	2.90LEL + 03	1.06LEL + 03	6.67LEL + 01	5.54LEL + 01	2.04LEL + 03	1.73LEL + 03	2.77LEL + 03	2.74LEL + 03
F29	AVG	7.91LEL + 03	1.09LEL + 04	1.52LEL + 04	1.13LEL + 04	6.27LEL + 05	1.87LEL + 04	1.10LEL + 04	**7.87LEL + 03**	3.02LEL + 04	1.20LEL + 04	2.57LEL + 04	1.62LEL + 04
	STD	8.12LEL + 02	2.52LEL + 03	3.15LEL + 03	2.10LEL + 03	4.26LEL + 05	3.32LEL + 03	**6.37LEL + 02**	7.39LEL + 02	1.43LEL + 04	6.69LEL + 03	4.45LEL + 03	3.69LEL + 03
F30	AVG	2.06LEL + 06	1.27LEL + 08	4.08LEL + 09	4.00LEL + 09	4.06LEL + 10	1.36LEL + 09	2.52LEL + 08	**1.16LEL + 06**	1.14LEL + 10	3.52LEL + 09	1.15LEL + 10	2.57LEL + 09
	STD	1.68LEL + 06	8.11LEL + 07	1.59LEL + 09	2.40LEL + 09	8.22LEL + 09	4.50LEL + 08	8.51LEL + 07	**6.56LEL + 05**	3.53LEL + 09	3.31LEL + 09	3.00LEL + 09	4.66LEL + 09
Friedman Rank		2.11	6.93	6.62	6.42	9.72	7.52	4.61	3.52	8.82	7.57	7.83	6.66
Final rank		**1**	**7**	**5**	**4**	**12**	**8**	**3**	**2**	**11**	**9**	**10**	**6**

#### CEC2017 convergence analysis

**Fig. 4:**
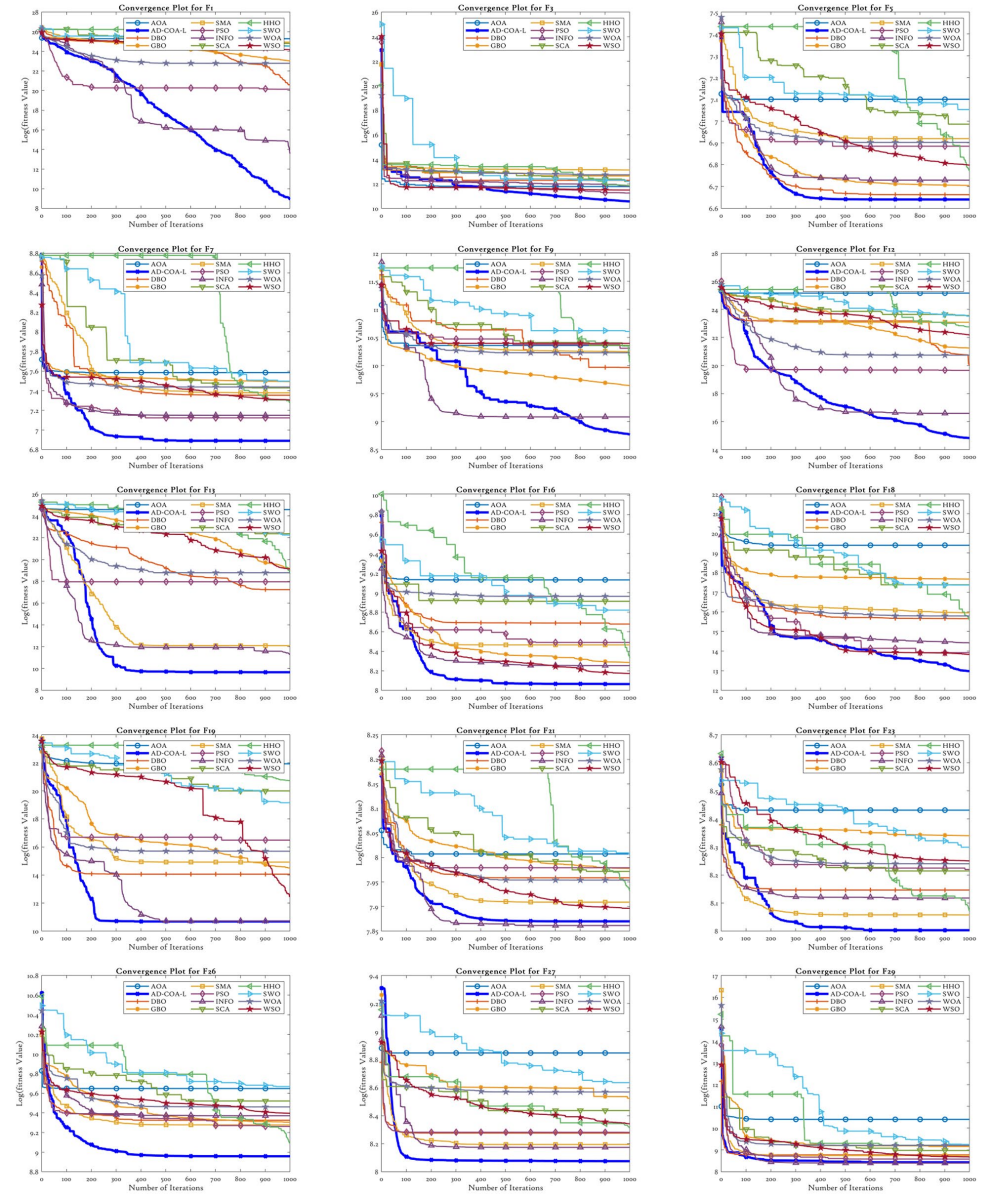
Convergence analysis for AD-COA-L and its rival algorithms using CEC2017, D = 50.

**Fig. 5:**
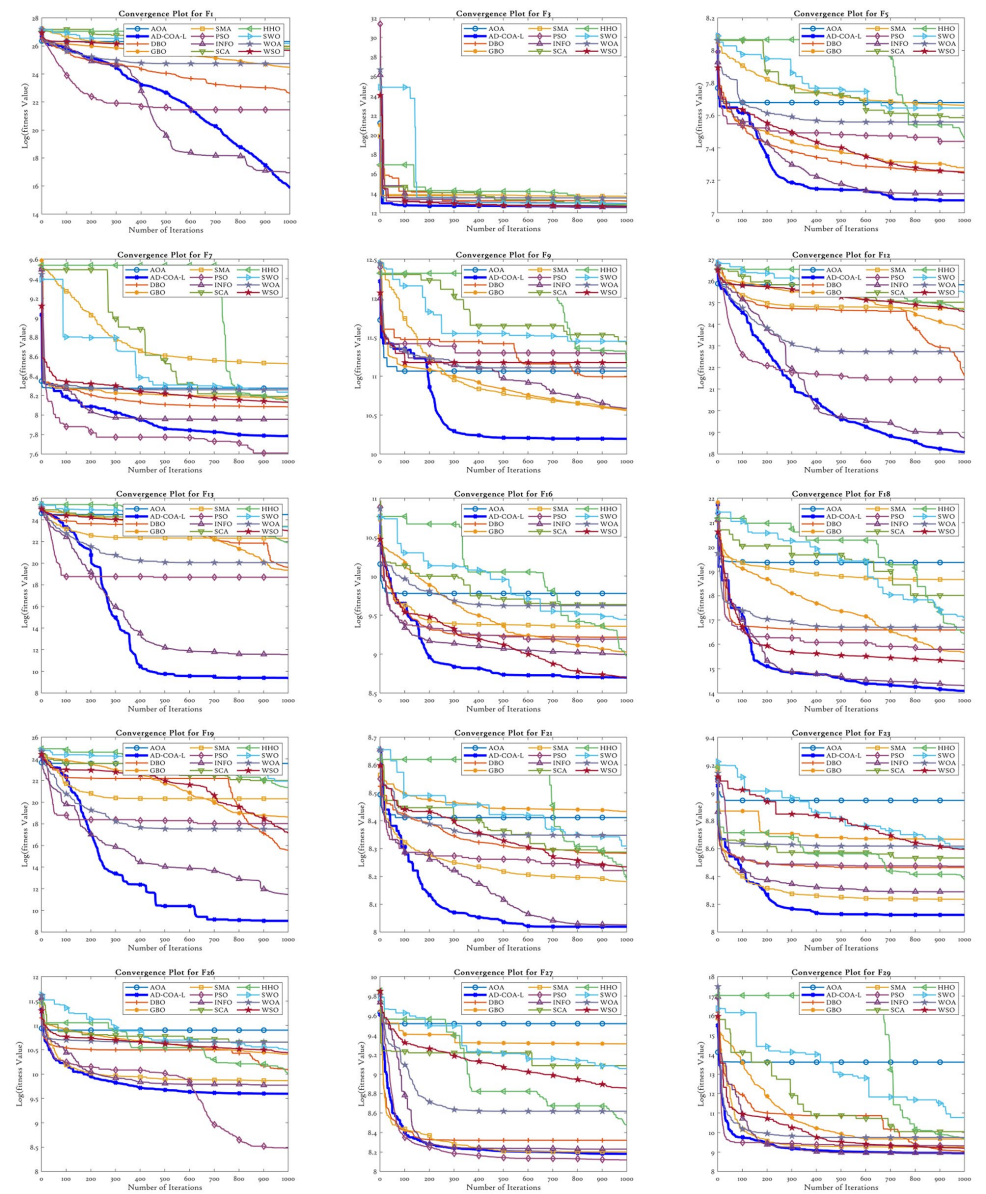
Convergence analysis for AD-COA-L and its rival algorithms using CEC2017, D = 100.

Figures 4 and 5 depict the convergence rate and accuracy of the AD-COA-L, SWO, COA, SO, AOA, HHO, INFO, PSO, SMA, SCA, and GBO algorithms in comparison to CEC2017 for the dimensions D = 50 and D = 100. The data demonstrates that AD-COA-L has a higher rate of convergence, reduced variability, and greater stability when compared to the other algorithms. Hence, AD-COA-L possesses the capacity to quickly attain the most favorable answers, thereby improving problem-solving effectiveness and adaptability. AD-COA-L consistently exhibits improved convergence on the convergence curve in the majority of test cases. This suggests that its search capacity gradually improves with each repetition, allowing it to effectively locate the best solutions for optimization problems. When applied to unimodal functions F1 and F3, the AD-COA-L algorithm has a higher convergence rate compared to other techniques. While INFO may outperform AD-COA-L in the early iterations, AD-COA-L ultimately gets higher results due to its new Bernoulli approach for population initialization. AD-COA-L demonstrates a higher rate of convergence in comparison to all alternative approaches. Function F5 provides evidence that AD-COA-L consistently obtains the lowest fitness values before the 300th iteration, surpassing all other algorithms in performance. Therefore, it can be deduced that AD-COA-L demonstrates a swift convergence rate, most likely because of its innovative exploitation technique and improved exploration formula. This enhances the algorithm’s ability to both explore and exploit. Once again, when assessing the performance of function F7 at CEC2017 with a dimensionality of 50, AD-COA-L demonstrates superior performance compared to all other rivals. This is attributed to its faster convergence rate and the attainment of the lowest value. Nevertheless, when evaluating F7 with D = 100, PSO exhibits remarkable performance. However, the difference in convergence rate between AD-COA-L and PSO is negligible. Thus, we can infer that the performance of AD-COA-L is praiseworthy.

### Comparison of AD-COA-L with advanced algorithms

This experiment is undertaken to further evaluate the performance of AD-COA-L in comparison to high-performing algorithms. Eight sophisticated and high-performing algorithms are employed to thoroughly assess the accuracy and effectiveness of AD-COA-L. The algorithms can be categorized into two groups. The first group consists of five advanced optimization algorithms: CSOAOA^48^, CJADE^70^, RLTLBO^71^, ASMA^72^, and TLABC^73^, and the second group constitute three winning algorithms in IEEE CEC, which are proven to perform excellently, namely, CMAES^74^, IMODE^75^, and AGSK^76^. These algorithms have been demonstrated to perform exceptionally well. The table labeled Table 8 contains the statistical standard deviation and mean of fitness values. These data were acquired from 30 independent runs using twenty-nine benchmark functions from CEC2017. The dimensionality of these functions is 50.

The analysis reveals that the AD-COA-L algorithm achieves the lowest average fitness value in 17 out of 29 functions, surpassing all other algorithms. The AD-COA-L algorithm has the greatest number of superior functions compared to all other algorithms. For instance, AD-COA-L demonstrates outstanding performance in unimodal functions F1 and F3, indicating superior values for both standard deviation and mean of fitness. The IMODE algorithm reports the optimal STD values in F4, while the AD-COA-L function guarantees the highest average fitness values.

**Fig. 6:**
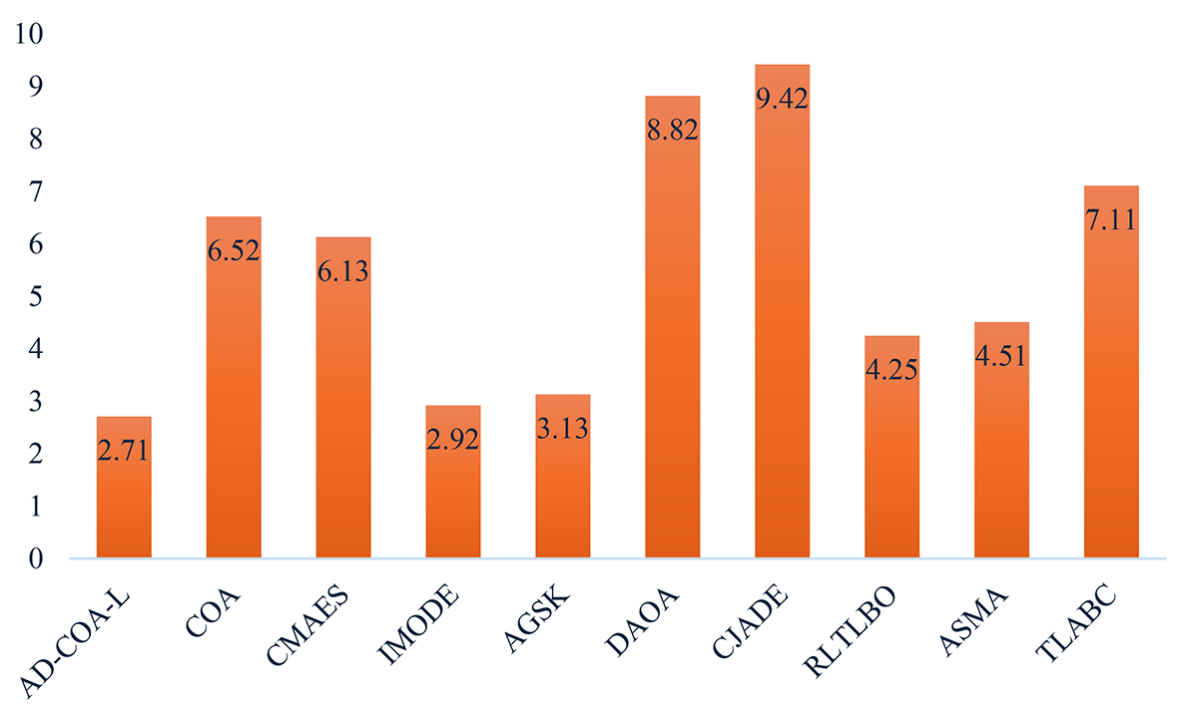
Friedman rank comparison between AD-COA-L and other algorithms.

However, the original COA approach does not demonstrate exceptional performance in any function. The suggested modifications in AD-COA-L enhance the balance between exploration and exploitation, resulting in the highest quality overall solution in terms of the ideal global optima. According to the findings in Table 8, the AD-COA-L algorithm shows great potential in the field of optimization. It outperforms algorithms that are very proficient in the field by being capable of solving global optimization tasks. The last row in Table 8; Fig. 6 represents the Friedman rank between the comparative algorithms where AD-COA-L is ranked the first with 2.71 while IMODE is the second with rank of 2.92 indicating the superior performance of AD-COA-L compared to a set of advanced and champion algorithms.

**Table 8:** Comparative analysis between AD-COA-L and its high-performing rivals using CEC2017.

F		AD-COA-L	COA	CMAES	IMODE	AGSK	DAOA	CJADE	RLTLBO	ASMA	TLABC
F1	AVG	**1.43LEL + 04**	4.73LEL + 09	1.37LEL + 10	6.05LEL + 05	1.06LEL + 07	2.57LEL + 11	2.68LEL + 11	2.65LEL + 09	5.22LEL + 09	8.89LEL + 10
	STD	**9.26LEL + 03**	1.10LEL + 10	2.41LEL + 10	4.32LEL + 05	1.63LEL + 07	2.73LEL + 10	2.57LEL + 10	2.58LEL + 09	2.52LEL + 09	1.05LEL + 10
F3	AVG	**8.87LEL + 04**	2.34LEL + 05	3.87LEL + 05	2.33LEL + 05	2.18LEL + 05	9.60LEL + 07	5.11LEL + 11	1.24LEL + 05	1.26LEL + 05	1.84LEL + 05
	STD	**1.43LEL + 04**	5.09LEL + 04	4.82LEL + 04	2.64LEL + 04	3.33LEL + 04	4.71LEL + 08	1.10LEL + 12	2.38LEL + 04	1.97LEL + 04	3.19LEL + 04
F4	AVG	**5.17LEL + 02**	9.67LEL + 02	7.68LEL + 03	5.58LEL + 02	6.12LEL + 02	1.18LEL + 05	1.22LEL + 05	9.36LEL + 02	9.35LEL + 02	2.81LEL + 04
	STD	5.69LEL + 01	3.77LEL + 02	1.96LEL + 03	**4.01LEL + 01**	4.82LEL + 01	1.92LEL + 04	2.17LEL + 04	2.02LEL + 02	2.12LEL + 02	5.80LEL + 03
F5	AVG	7.99LEL + 02	9.92LEL + 02	**5.92LEL + 02**	7.47LEL + 02	9.16LEL + 02	1.74LEL + 03	1.72LEL + 03	8.44LEL + 02	7.28LEL + 02	1.10LEL + 03
	STD	**1.89LEL + 01**	9.58LEL + 01	1.44LEL + 02	5.09LEL + 01	3.31LEL + 01	7.44LEL + 01	7.46LEL + 01	4.91LEL + 01	7.12LEL + 01	4.83LEL + 01
F6	AVG	**6.01LEL + 02**	6.64LEL + 02	6.37LEL + 02	6.43LEL + 02	6.19LEL + 02	7.56LEL + 02	7.56LEL + 02	6.43LEL + 02	6.16LEL + 02	6.83LEL + 02
	STD	**2.79LEL-01**	1.33LEL + 01	3.33LEL + 01	8.46LEL + 00	4.77LEL + 00	9.07LEL + 00	1.07LEL + 01	9.75LEL + 00	4.67LEL + 00	5.28LEL + 00
F7	AVG	**9.00LEL + 02**	1.47LEL + 03	1.26LEL + 03	1.03LEL + 03	1.21LEL + 03	6.15LEL + 03	6.10LEL + 03	1.39LEL + 03	1.07LEL + 03	1.88LEL + 03
	STD	1.22LEL + 02	1.58LEL + 02	1.25LEL + 02	**2.65LEL + 01**	5.04LEL + 01	4.03LEL + 02	5.58LEL + 02	1.62LEL + 02	8.27LEL + 01	9.69LEL + 01
F8	AVG	1.08LEL + 03	1.29LEL + 03	**1.01LEL + 03**	1.05LEL + 03	1.22LEL + 03	1.97LEL + 03	2.08LEL + 03	1.17LEL + 03	1.06LEL + 03	1.40LEL + 03
	STD	4.37LEL + 01	8.26LEL + 01	2.45LEL + 02	**2.42LEL + 01**	3.72LEL + 01	9.97LEL + 01	7.57LEL + 01	6.26LEL + 01	8.12LEL + 01	4.36LEL + 01
F9	AVG	1.25LEL + 04	2.16LEL + 04	**2.89LEL + 03**	8.89LEL + 03	5.51LEL + 03	1.06LEL + 05	1.07LEL + 05	1.95LEL + 04	8.97LEL + 03	2.55LEL + 04
	STD	4.99LEL + 03	9.01LEL + 03	5.27LEL + 03	**2.25LEL + 03**	2.76LEL + 03	1.33LEL + 04	1.13LEL + 04	7.17LEL + 03	4.74LEL + 03	3.49LEL + 03
F10	AVG	1.22LEL + 04	1.07LEL + 04	1.49LEL + 04	8.71LEL + 03	**7.48LEL + 03**	1.71LEL + 04	1.85LEL + 04	1.16LEL + 04	8.80LEL + 03	1.40LEL + 04
	STD	1.57LEL + 03	1.97LEL + 03	4.12LEL + 02	5.79LEL + 02	**3.64LEL + 02**	4.83LEL + 02	7.56LEL + 02	1.79LEL + 03	2.74LEL + 03	8.16LEL + 02
F11	AVG	**1.40LEL + 03**	2.62LEL + 03	7.14LEL + 04	1.40LEL + 03	1.56LEL + 03	9.02LEL + 04	8.17LEL + 06	1.51LEL + 03	3.91LEL + 03	1.93LEL + 04
	STD	1.05LEL + 02	1.31LEL + 03	1.51LEL + 04	8.61LEL + 01	**7.61LEL + 01**	3.03LEL + 04	2.12LEL + 07	1.55LEL + 02	1.23LEL + 03	4.30LEL + 03
F12	AVG	**3.66LEL + 06**	6.75LEL + 08	2.07LEL + 10	7.02LEL + 06	9.15LEL + 06	1.45LEL + 11	1.46LEL + 11	2.72LEL + 07	8.54LEL + 08	4.73LEL + 10
	STD	**2.43LEL + 06**	7.47LEL + 08	5.34LEL + 09	3.86LEL + 06	5.49LEL + 06	2.38LEL + 10	3.12LEL + 10	3.02LEL + 07	1.39LEL + 09	1.11LEL + 10
F13	AVG	5.48LEL + 04	5.13LEL + 07	1.07LEL + 10	1.87LEL + 04	**1.24LEL + 04**	9.18LEL + 10	9.00LEL + 10	3.14LEL + 04	1.22LEL + 08	2.36LEL + 10
	STD	3.19LEL + 04	6.95LEL + 07	2.90LEL + 09	2.37LEL + 04	**1.45LEL + 04**	1.87LEL + 10	2.96LEL + 10	4.66LEL + 04	1.49LEL + 08	9.25LEL + 09
F14	AVG	2.58LEL + 05	3.11LEL + 06	2.13LEL + 07	3.16LEL + 05	6.22LEL + 04	3.91LEL + 08	5.19LEL + 08	**6.15LEL + 04**	1.29LEL + 06	2.34LEL + 07
	STD	**5.09LEL + 04**	3.73LEL + 06	1.30LEL + 07	4.07LEL + 05	1.64LEL + 05	1.62LEL + 08	2.53LEL + 08	5.80LEL + 04	1.37LEL + 06	2.52LEL + 07
F15	AVG	**1.31LEL + 04**	2.15LEL + 07	1.82LEL + 09	1.33LEL + 04	2.02LEL + 04	2.86LEL + 10	3.85LEL + 10	1.56LEL + 04	1.10LEL + 07	3.94LEL + 09
	STD	7.63LEL + 03	7.20LEL + 07	7.82LEL + 08	1.10LEL + 04	1.26LEL + 04	9.26LEL + 09	9.04LEL + 09	**5.52LEL + 03**	1.69LEL + 07	2.29LEL + 09
F16	AVG	**3.23LEL + 03**	4.87LEL + 03	6.79LEL + 03	3.64LEL + 03	4.10LEL + 03	1.55LEL + 04	1.69LEL + 04	3.27LEL + 03	3.74LEL + 03	6.62LEL + 03
	STD	5.25LEL + 02	5.40LEL + 02	4.35LEL + 02	2.61LEL + 02	**1.97LEL + 02**	3.48LEL + 03	3.72LEL + 03	4.18LEL + 02	5.22LEL + 02	6.87LEL + 02
F17	AVG	3.31LEL + 03	4.19LEL + 03	**2.85LEL + 03**	3.02LEL + 03	3.39LEL + 03	1.41LEL + 06	2.07LEL + 06	3.18LEL + 03	2.93LEL + 03	4.31LEL + 03
	STD	3.32LEL + 02	4.76LEL + 02	3.58LEL + 02	**1.43LEL + 02**	1.61LEL + 02	1.92LEL + 06	2.01LEL + 06	3.49LEL + 02	3.66LEL + 02	7.02LEL + 02
F18	AVG	**7.78LEL + 05**	8.16LEL + 06	1.05LEL + 08	2.33LEL + 06	1.24LEL + 06	6.47LEL + 08	1.56LEL + 09	1.33LEL + 06	5.25LEL + 06	3.98LEL + 07
	STD	**4.27LEL + 05**	9.49LEL + 06	5.63LEL + 07	1.68LEL + 06	7.78LEL + 05	2.47LEL + 08	7.40LEL + 08	8.64LEL + 05	4.80LEL + 06	2.63LEL + 07
F19	AVG	2.70LEL + 04	5.69LEL + 06	1.08LEL + 09	2.17LEL + 04	**1.14LEL + 04**	1.42LEL + 10	1.51LEL + 10	1.94LEL + 04	1.75LEL + 06	1.35LEL + 09
	STD	1.44LEL + 04	5.57LEL + 06	7.36LEL + 08	**5.94LEL + 03**	8.23LEL + 03	3.95LEL + 09	4.37LEL + 09	1.07LEL + 04	1.67LEL + 06	7.25LEL + 08
F20	AVG	3.16LEL + 03	3.72LEL + 03	3.77LEL + 03	3.18LEL + 03	3.45LEL + 03	5.33LEL + 03	5.77LEL + 03	3.13LEL + 03	**3.03LEL + 03**	3.56LEL + 03
	STD	3.69LEL + 02	2.96LEL + 02	2.60LEL + 02	2.17LEL + 02	**1.81LEL + 02**	2.20LEL + 02	3.42LEL + 02	2.31LEL + 02	3.57LEL + 02	3.14LEL + 02
F21	AVG	**2.51LEL + 03**	2.85LEL + 03	2.62LEL + 03	2.54LEL + 03	2.70LEL + 03	3.58LEL + 03	3.59LEL + 03	2.57LEL + 03	2.58LEL + 03	2.98LEL + 03
	STD	5.36LEL + 01	8.75LEL + 01	2.66LEL + 02	**2.64LEL + 01**	3.10LEL + 01	9.25LEL + 01	1.11LEL + 02	5.78LEL + 01	4.75LEL + 01	6.53LEL + 01
F22	AVG	9.97LEL + 03	1.25LEL + 04	1.65LEL + 04	8.78LEL + 03	1.36LEL + 04	1.86LEL + 04	2.00LEL + 04	**5.61LEL + 03**	9.97LEL + 03	1.54LEL + 04
	STD	2.65LEL + 03	2.19LEL + 03	**5.77LEL + 02**	2.10LEL + 03	1.80LEL + 03	6.22LEL + 02	7.89LEL + 02	3.23LEL + 03	2.35LEL + 03	9.15LEL + 02
F23	AVG	**2.98LEL + 03**	3.51LEL + 03	3.44LEL + 03	3.01LEL + 03	3.14LEL + 03	5.37LEL + 03	5.29LEL + 03	3.12LEL + 03	3.25LEL + 03	4.00LEL + 03
	STD	1.35LEL + 02	1.26LEL + 02	4.15LEL + 01	**2.41LEL + 01**	4.79LEL + 01	4.34LEL + 02	3.88LEL + 02	8.96LEL + 01	3.68LEL + 01	1.42LEL + 02
F24	AVG	**3.18LEL + 03**	3.64LEL + 03	3.52LEL + 03	3.22LEL + 03	3.28LEL + 03	6.09LEL + 03	6.08LEL + 03	3.34LEL + 03	3.42LEL + 03	4.37LEL + 03
	STD	1.35LEL + 02	1.32LEL + 02	3.84LEL + 01	**2.61LEL + 01**	4.92LEL + 01	5.25LEL + 02	4.80LEL + 02	8.86LEL + 01	1.07LEL + 02	3.03LEL + 02
F25	AVG	**3.07LEL + 03**	3.99LEL + 03	3.99LEL + 03	3.11LEL + 03	3.11LEL + 03	6.36LEL + 04	6.52LEL + 04	3.41LEL + 03	3.56LEL + 03	1.36LEL + 04
	STD	**3.15LEL + 01**	1.94LEL + 03	1.49LEL + 03	3.20LEL + 01	3.68LEL + 01	9.65LEL + 03	1.05LEL + 04	1.55LEL + 02	3.00LEL + 02	1.60LEL + 03
F26	AVG	**6.55LEL + 03**	1.04LEL + 04	1.14LEL + 04	7.52LEL + 03	7.88LEL + 03	3.40LEL + 04	3.46LEL + 04	1.06LEL + 04	6.72LEL + 03	1.60LEL + 04
	STD	**2.63LEL + 02**	1.40LEL + 03	4.87LEL + 02	2.73LEL + 03	9.72LEL + 02	4.96LEL + 03	5.33LEL + 03	2.20LEL + 03	7.58LEL + 02	8.77LEL + 02
F27	AVG	3.65LEL + 03	3.92LEL + 03	3.87LEL + 03	3.55LEL + 03	**3.50LEL + 03**	9.01LEL + 03	9.28LEL + 03	3.69LEL + 03	3.61LEL + 03	5.59LEL + 03
	STD	1.65LEL + 02	2.47LEL + 02	8.89LEL + 01	**5.16LEL + 01**	7.67LEL + 01	1.22LEL + 03	1.16LEL + 03	1.09LEL + 02	1.07LEL + 02	5.07LEL + 02
F28	AVG	**3.34LEL + 03**	6.10LEL + 03	9.71LEL + 03	3.41LEL + 03	3.40LEL + 03	2.62LEL + 04	2.52LEL + 04	3.85LEL + 03	4.16LEL + 03	1.10LEL + 04
	STD	3.78LEL + 01	2.48LEL + 03	3.74LEL + 02	**2.96LEL + 01**	4.88LEL + 01	4.05LEL + 03	3.65LEL + 03	1.86LEL + 02	3.39LEL + 02	1.09LEL + 03
F29	AVG	**4.14LEL + 03**	6.27LEL + 03	1.22LEL + 04	5.14LEL + 03	4.94LEL + 03	2.28LEL + 06	4.51LEL + 06	5.16LEL + 03	4.61LEL + 03	1.56LEL + 04
	STD	**2.24LEL + 02**	9.21LEL + 02	2.86LEL + 03	4.15LEL + 02	2.40LEL + 02	2.14LEL + 06	4.26LEL + 06	4.33LEL + 02	3.21LEL + 02	6.79LEL + 03
F30	AVG	3.32LEL + 06	4.42LEL + 07	1.88LEL + 09	5.05LEL + 06	3.49LEL + 06	1.97LEL + 10	2.38LEL + 10	**1.43LEL + 06**	1.21LEL + 08	2.53LEL + 09
	STD	1.70LEL + 06	4.81LEL + 07	6.47LEL + 08	1.10LEL + 06	1.56LEL + 06	5.27LEL + 09	6.49LEL + 09	**6.99LEL + 05**	4.23LEL + 07	1.76LEL + 09
Friedman rank		2.71	6.52	6.13	2.92	3.13	8.82	9.42	4.25	4.51	7.11
Final rank		1	7	6	2	3	9	10	4	5	8

### Statistical analysis of AD-COA-L

The Wilcoxon rank sum test^77^ can be employed as a nonparametric statistical test to assess if the comparative AD-COA-L approach is statistically distinct from the other methods. The objective was achieved by doing 30 individual runs for each of the competing algorithms, utilizing a standardized set of 29 test functions. The Wilcoxon rank sum test is performed at a significance level of 0.05 to assess the significant difference between the solution results of the six algorithms being studied and those of the AD-COA-L algorithm. The statistical test results are aggregated and presented in Tables 9 and 10. To confirm these findings, if the $\:p$-value is less than 0.05, we can reject the null hypothesis and conclude that there is a significant difference between the algorithms being studied. Alternatively, if the $\:p$-value is greater than 0.05, the search results obtained from the two methods are compared. Tables 9 and 10 clearly demonstrate that the AD-COA-L algorithm exhibits substantial disparities when compared to the other approaches. AD-COA-L exhibits significant superiority when compared to WOA, SWO, WSO, HHO, PSO, COA, INFO, AOA, SMA, SCA, and GBO. The statistical significance of the advantage of the AD-COA-L algorithm has been determined.

**Table 9:** Statistical analysis based on Wilcoxon test between AD-COA-L and its rivals using CEC2017, D = 50.

F	AOA	COA	GBO	SMA	PSO	INFO	SCA	HHO	SWO	WOA	WSO
C17-G1	1.734398LEL-06	1.734398LEL-06	1.734398LEL-06	1.734398LEL-06	1.734398LEL-06	1.734398LEL-06	1.734398LEL-06	1.734398LEL-06	1.734398LEL-06	1.734398LEL-06	1.734398LEL-06
C17-G3	1.734398LEL-06	9.271025LEL-03	6.319757LEL-05	1.254382LEL-01	1.734398LEL-06	1.483928LEL-03	1.734398LEL-06	1.734398LEL-06	1.734398LEL-06	1.734398LEL-06	9.777219LEL-02
C17-G4	1.734398LEL-06	1.734398LEL-06	1.734398LEL-06	1.734398LEL-06	1.734398LEL-06	6.835856LEL-03	1.734398LEL-06	1.734398LEL-06	1.734398LEL-06	1.734398LEL-06	1.734398LEL-06
C17-G5	1.734398LEL-06	1.734398LEL-06	1.734398LEL-06	1.734398LEL-06	1.734398LEL-06	1.382036LEL-03	1.734398LEL-06	1.734398LEL-06	1.734398LEL-06	1.734398LEL-06	1.734398LEL-06
C17-G6	1.734398LEL-06	1.734398LEL-06	1.734398LEL-06	1.734398LEL-06	1.734398LEL-06	1.734398LEL-06	1.734398LEL-06	1.734398LEL-06	1.734398LEL-06	1.734398LEL-06	1.734398LEL-06
C17-G7	1.734398LEL-06	4.285686LEL-06	1.734398LEL-06	6.339136LEL-06	1.254382LEL-01	2.613431LEL-04	1.734398LEL-06	1.734398LEL-06	1.734398LEL-06	1.734398LEL-06	8.944301LEL-04
C17-G8	1.734398LEL-06	1.920921LEL-06	1.126540LEL-05	2.353421LEL-06	1.734398LEL-06	1.964581LEL-03	1.734398LEL-06	1.734398LEL-06	1.734398LEL-06	1.734398LEL-06	1.197338LEL-03
C17-G9	1.734398LEL-06	3.882182LEL-06	1.734398LEL-06	1.972948LEL-05	2.126636LEL-06	2.584559LEL-03	1.734398LEL-06	2.126636LEL-06	1.734398LEL-06	1.734398LEL-06	2.369362LEL-01
C17-G10	1.734398LEL-06	1.493564LEL-05	1.286631LEL-03	2.596713LEL-05	3.609433LEL-03	8.466082LEL-06	1.734398LEL-06	7.730944LEL-03	1.734398LEL-06	8.187753LEL-05	8.466082LEL-06
C17-G11	1.734398LEL-06	1.890972LEL-04	1.734398LEL-06	1.238080LEL-05	3.872303LEL-03	1.734398LEL-06	1.734398LEL-06	3.181679LEL-06	1.734398LEL-06	1.734398LEL-06	8.466082LEL-06
C17-G12	1.734398LEL-06	1.734398LEL-06	1.734398LEL-06	4.285686LEL-06	1.734398LEL-06	1.734398LEL-06	1.734398LEL-06	1.734398LEL-06	1.734398LEL-06	1.734398LEL-06	5.304401LEL-01
C17-G13	1.734398LEL-06	1.734398LEL-06	1.734398LEL-06	1.734398LEL-06	5.709650LEL-02	1.734398LEL-06	1.734398LEL-06	1.734398LEL-06	1.734398LEL-06	1.734398LEL-06	1.734398LEL-06
C17-G14	2.603328LEL-06	4.729202LEL-06	3.181679LEL-06	4.729202LEL-06	4.729202LEL-06	7.970983LEL-01	1.734398LEL-06	1.734398LEL-06	1.734398LEL-06	1.734398LEL-06	4.491890LEL-02
C17-G15	1.734398LEL-06	1.734398LEL-06	1.734398LEL-06	2.126636LEL-06	1.734398LEL-06	3.064999LEL-04	1.734398LEL-06	1.734398LEL-06	1.734398LEL-06	1.734398LEL-06	3.405257LEL-05
C17-G16	1.734398LEL-06	2.848596LEL-02	5.306992LEL-05	1.319417LEL-02	5.751653LEL-06	1.890972LEL-04	1.734398LEL-06	1.734398LEL-06	4.285686LEL-06	1.920921LEL-06	8.466082LEL-06
C17-G17	1.734398LEL-06	1.920921LEL-06	1.734398LEL-06	1.734398LEL-06	1.920921LEL-06	1.734398LEL-06	1.734398LEL-06	1.734398LEL-06	1.734398LEL-06	1.734398LEL-06	1.734398LEL-06
C17-G18	1.734398LEL-06	1.734398LEL-06	1.734398LEL-06	2.603328LEL-06	1.734398LEL-06	2.126636LEL-06	1.734398LEL-06	1.734398LEL-06	1.734398LEL-06	1.734398LEL-06	1.734398LEL-06
C17-G19	1.734398LEL-06	2.603328LEL-06	1.734398LEL-06	1.734398LEL-06	6.583305LEL-04	1.742281LEL-04	1.734398LEL-06	1.734398LEL-06	1.734398LEL-06	1.734398LEL-06	1.734398LEL-06
C17-G20	1.734398LEL-06	2.414704LEL-03	5.792446LEL-05	3.854236LEL-03	3.600388LEL-01	6.268281LEL-02	1.734398LEL-06	1.779074LEL-01	1.734398LEL-06	1.734398LEL-06	2.843424LEL-05
C17-G21	1.734398LEL-06	3.112315LEL-05	1.238080LEL-05	2.224827LEL-04	1.149922LEL-04	2.105260LEL-03	2.126636LEL-06	4.071512LEL-05	2.126636LEL-06	3.181679LEL-06	1.798848LEL-05
C17-G22	1.734398LEL-06	1.734398LEL-06	1.920921LEL-06	1.734398LEL-06	8.220647LEL-02	1.734398LEL-06	1.734398LEL-06	1.734398LEL-06	1.734398LEL-06	1.734398LEL-06	1.734398LEL-06
C17-G23	1.734398LEL-06	7.513662LEL-05	1.734398LEL-06	2.613431LEL-04	8.466082LEL-06	2.957462LEL-03	1.734398LEL-06	1.734398LEL-06	1.734398LEL-06	1.734398LEL-06	7.513662LEL-05
C17-G24	1.734398LEL-06	1.734398LEL-06	1.734398LEL-06	1.734398LEL-06	1.734398LEL-06	1.734398LEL-06	1.734398LEL-06	1.734398LEL-06	1.734398LEL-06	1.734398LEL-06	1.734398LEL-06
C17-G25	1.734398LEL-06	1.734398LEL-06	1.734398LEL-06	1.734398LEL-06	1.734398LEL-06	2.613431LEL-04	1.734398LEL-06	1.734398LEL-06	1.734398LEL-06	1.734398LEL-06	1.734398LEL-06
C17-G26	1.734398LEL-06	1.734398LEL-06	1.734398LEL-06	1.734398LEL-06	6.319757LEL-05	1.846219LEL-01	1.734398LEL-06	1.734398LEL-06	1.734398LEL-06	1.734398LEL-06	1.734398LEL-06
C17-G27	1.734398LEL-06	4.071512LEL-05	6.983783LEL-06	1.734398LEL-06	1.734398LEL-06	7.521331LEL-02	1.734398LEL-06	1.734398LEL-06	1.734398LEL-06	1.734398LEL-06	1.734398LEL-06
C17-G28	1.734398LEL-06	1.024633LEL-05	3.493456LEL-01	1.020107LEL-01	1.493564LEL-05	6.339136LEL-06	1.126540LEL-05	6.564114LEL-02	5.216493LEL-06	2.603328LEL-06	2.411796LEL-04
C17-G29	1.734398LEL-06	2.596713LEL-05	1.734398LEL-06	1.734398LEL-06	1.734398LEL-06	1.986102LEL-01	1.734398LEL-06	1.734398LEL-06	1.734398LEL-06	1.734398LEL-06	1.734398LEL-06
C17-G30	1.734398LEL-06	3.181679LEL-06	3.181679LEL-06	1.734398LEL-06	1.734398LEL-06	7.655193LEL-01	1.734398LEL-06	1.734398LEL-06	1.734398LEL-06	1.734398LEL-06	1.734398LEL-06

**Table 10:** Statistical analysis based on Wilcoxon test between AD-COA-L and its rivals using CEC2017, D = 100.

F	AOA	COA	GBO	SMA	PSO	INFO	SCA	HHO	SWO	WOA	WSO
C17-G1	1.734398LEL-06	1.734398LEL-06	1.734398LEL-06	1.734398LEL-06	1.734398LEL-06	1.734398LEL-06	1.734398LEL-06	1.734398LEL-06	1.734398LEL-06	1.734398LEL-06	1.734398LEL-06
C17-G3	1.734398LEL-06	9.271025LEL-03	6.319757LEL-05	1.254382LEL-01	1.734398LEL-06	1.483928LEL-03	1.734398LEL-06	1.734398LEL-06	1.734398LEL-06	1.734398LEL-06	9.777219LEL-02
C17-G4	1.734398LEL-06	1.734398LEL-06	1.734398LEL-06	1.734398LEL-06	1.734398LEL-06	6.835856LEL-03	1.734398LEL-06	1.734398LEL-06	1.734398LEL-06	1.734398LEL-06	1.734398LEL-06
C17-G5	1.734398LEL-06	1.734398LEL-06	1.734398LEL-06	1.734398LEL-06	1.734398LEL-06	1.382036LEL-03	1.734398LEL-06	1.734398LEL-06	1.734398LEL-06	1.734398LEL-06	1.734398LEL-06
C17-G6	1.734398LEL-06	1.734398LEL-06	1.734398LEL-06	1.734398LEL-06	1.734398LEL-06	1.734398LEL-06	1.734398LEL-06	1.734398LEL-06	1.734398LEL-06	1.734398LEL-06	1.734398LEL-06
C17-G7	1.734398LEL-06	4.285686LEL-06	1.734398LEL-06	6.339136LEL-06	1.734398LEL-06	1.846219LEL-01	1.734398LEL-06	1.734398LEL-06	1.734398LEL-06	1.734398LEL-06	8.944301LEL-04
C17-G8	1.734398LEL-06	1.920921LEL-06	1.126540LEL-05	2.353421LEL-06	1.734398LEL-06	1.964581LEL-03	1.734398LEL-06	1.734398LEL-06	1.734398LEL-06	1.734398LEL-06	1.197338LEL-03
C17-G9	1.734398LEL-06	3.882182LEL-06	1.734398LEL-06	1.972948LEL-05	2.126636LEL-06	2.584559LEL-03	1.734398LEL-06	2.126636LEL-06	1.734398LEL-06	1.734398LEL-06	1.734398LEL-06
C17-G10	1.734398LEL-06	1.493564LEL-05	1.286631LEL-03	2.596713LEL-05	3.609433LEL-03	8.466082LEL-06	1.734398LEL-06	7.730944LEL-03	1.734398LEL-06	8.187753LEL-05	8.466082LEL-06
C17-G11	1.734398LEL-06	1.890972LEL-04	1.734398LEL-06	1.238080LEL-05	1.734398LEL-06	2.058882LEL-01	1.734398LEL-06	3.181679LEL-06	1.734398LEL-06	1.734398LEL-06	8.944301LEL-04
C17-G12	1.734398LEL-06	1.734398LEL-06	1.734398LEL-06	4.285686LEL-06	1.734398LEL-06	6.564114LEL-02	1.734398LEL-06	1.734398LEL-06	1.734398LEL-06	1.734398LEL-06	5.304401LEL-01
C17-G13	1.734398LEL-06	1.734398LEL-06	1.734398LEL-06	1.734398LEL-06	1.734398LEL-06	1.734398LEL-06	1.734398LEL-06	1.734398LEL-06	1.734398LEL-06	1.734398LEL-06	1.734398LEL-06
C17-G14	2.603328LEL-06	4.729202LEL-06	3.181679LEL-06	4.729202LEL-06	4.729202LEL-06	7.970983LEL-01	1.734398LEL-06	1.734398LEL-06	1.734398LEL-06	1.734398LEL-06	1.734398LEL-06
C17-G15	1.734398LEL-06	1.734398LEL-06	1.734398LEL-06	2.126636LEL-06	1.734398LEL-06	3.064999LEL-04	1.734398LEL-06	1.734398LEL-06	1.734398LEL-06	1.734398LEL-06	3.405257LEL-05
C17-G16	1.734398LEL-06	2.848596LEL-02	5.306992LEL-05	2.224827LEL-04	5.751653LEL-06	1.890972LEL-04	1.734398LEL-06	1.734398LEL-06	4.285686LEL-06	1.920921LEL-06	1.734398LEL-06
C17-G17	1.734398LEL-06	1.920921LEL-06	1.734398LEL-06	1.734398LEL-06	1.920921LEL-06	1.734398LEL-06	1.734398LEL-06	1.734398LEL-06	1.734398LEL-06	1.734398LEL-06	1.734398LEL-06
C17-G18	1.734398LEL-06	1.734398LEL-06	1.734398LEL-06	2.603328LEL-06	1.734398LEL-06	2.126636LEL-06	1.734398LEL-06	1.734398LEL-06	1.734398LEL-06	1.734398LEL-06	1.734398LEL-06
C17-G19	1.734398LEL-06	2.603328LEL-06	1.734398LEL-06	1.734398LEL-06	6.583305LEL-01	1.742281LEL-04	1.734398LEL-06	1.734398LEL-06	1.734398LEL-06	1.734398LEL-06	1.734398LEL-06
C17-G20	1.734398LEL-06	2.414704LEL-03	5.792446LEL-05	3.854236LEL-03	3.600388LEL-01	1.742281LEL-04	1.734398LEL-06	1.779074LEL-01	1.734398LEL-06	1.734398LEL-06	2.843424LEL-05
C17-G21	1.734398LEL-06	3.112315LEL-05	1.238080LEL-05	2.224827LEL-04	1.149922LEL-04	2.105260LEL-03	2.126636LEL-06	4.071512LEL-05	2.126636LEL-06	3.181679LEL-06	1.798848LEL-05
C17-G22	1.734398LEL-06	1.734398LEL-06	1.920921LEL-06	1.734398LEL-06	8.220647LEL-02	1.734398LEL-06	1.734398LEL-06	1.734398LEL-06	1.734398LEL-06	1.734398LEL-06	1.734398LEL-06
C17-G23	1.734398LEL-06	7.513662LEL-05	1.734398LEL-06	2.613431LEL-04	8.466082LEL-06	2.957462LEL-03	1.734398LEL-06	1.734398LEL-06	1.734398LEL-06	1.734398LEL-06	7.513662LEL-05
C17-G24	1.734398LEL-06	1.734398LEL-06	1.734398LEL-06	1.734398LEL-06	1.734398LEL-06	1.734398LEL-06	1.734398LEL-06	1.734398LEL-06	1.734398LEL-06	1.734398LEL-06	1.734398LEL-06
C17-G25	1.734398LEL-06	1.734398LEL-06	1.734398LEL-06	1.734398LEL-06	1.734398LEL-06	5.193067LEL-02	1.734398LEL-06	1.734398LEL-06	1.734398LEL-06	1.734398LEL-06	1.734398LEL-06
C17-G26	1.734398LEL-06	1.734398LEL-06	1.734398LEL-06	1.734398LEL-06	6.319757LEL-05	1.742281LEL-04	1.734398LEL-06	1.734398LEL-06	1.734398LEL-06	1.734398LEL-06	1.734398LEL-06
C17-G27	1.734398LEL-06	4.071512LEL-05	6.983783LEL-06	1.734398LEL-06	1.734398LEL-06	1.734398LEL-06	1.734398LEL-06	1.734398LEL-06	1.734398LEL-06	1.734398LEL-06	1.734398LEL-06
C17-G28	1.734398LEL-06	1.024633LEL-05	3.493456LEL-01	1.020107LEL-01	1.493564LEL-05	6.339136LEL-06	1.126540LEL-05	6.564114LEL-02	5.216493LEL-06	2.603328LEL-06	2.411796LEL-04
C17-G29	1.734398LEL-06	2.596713LEL-05	1.734398LEL-06	1.734398LEL-06	1.734398LEL-06	1.986102LEL-01	1.734398LEL-06	1.734398LEL-06	1.734398LEL-06	1.734398LEL-06	1.734398LEL-06
C17-G30	1.734398LEL-06	3.181679LEL-06	3.181679LEL-06	1.734398LEL-06	1.734398LEL-06	7.655193LEL-01	1.734398LEL-06	1.734398LEL-06	1.734398LEL-06	1.734398LEL-06	3.000989LEL-02

### Computational analysis

Computational analysis of different algorithms is a crucial factor that needs to be studies to assess the overall performance of the novel proposed algorithms. The computational analysis includes two main folds which are the time complexity and space complexity. The time complexity studies the theoretical computational runtime of different algorithms according to the most significant operations while the space complexity denotes the memory space required by the main variables and vectors of algorithms. This section studies the compactional analysis of the proposed AD-COA-l compared to the original COA and other compared algorithms.

#### Time complexity

There are three key parameters that directly influence the time complexity of the original COA including population size ($\:N$), dimensionality ($\:D$), and the number of iterations ($\:T$). COA generates an initial population in the initialization stage, and the main computational cost occurs in the updating of positions in the stages of summer resort, competition, and foraging. This updating process is done for each solution in the population and this whole process is repeated for all iterations. So, for original COA time complexity can be represented as:

$$\:O\left(COA\right)=\:O\left(ND\:+\:TND\right)=O\left(TND\right)$$

On the other hand, the general time complexity of the proposed AD-COA-L algorithm is similar to that of the original COA, with several enhancements added to it. Namely, Bernoulli Map-based Population Initialization, Adaptive Lens Opposite-Based Learning (ALOBL), and the Local Escaping Operator (LEO). In the Bernoulli map-based population initialization, the complexity remains $\:O\left(ND\right)$, similar to the original initialization process. In the adaptive lens opposite-based learning (ALOBL), it applied only to the best solution, contributing $\:O\left(TD\right)$ over the iterations. Furthermore, the local escaping operator (LEO) updates the positions of all solutions, contributing $\:O\left(TND\right)$. The dynamic inertia weight coefficient does not add more complexity since it is part of the position update equation of the original COA. Therefore, the total time complexity of the AD-COA-L algorithm is bound by:

$\:O\left(AD-COA-L\right)=\:O\left(ND\:+\:TND\right)=O\left(TND\right)$It is apparent that from the time complexity of each the original COA and the improved AD-COA-L that there is no major difference between them in the time complexity consumed by the CPU, but the performance obtained by AD-COA-L is much better than COA as conducted in the experiments.

#### Space complexity

Regarding space complexity, the original COA and the proposed AD-COA-L have the same space complexity because both algorithms deal with a population of size $\:N$ and a problem of dimensionality $\:D$. The number of memory usage used at any instant of time during the run of the algorithm increases linearly with respect to the population size and the number of dimensions under optimization. Hence, the following ensures the space complexity for both algorithms:

$$\:Space\:Complexity\:=\:O\left(N\:\times\:\:D\right)$$

Finally, Table 11 compare between the proposed AD-COA-L and its rivals regarding the time and space complexity obtained during different iterations. As shown by Table 11, the time complexity for all algorithms under comparison, including the proposed AD-COA-L algorithm, is $\:O\left(TND\right)$. This means that, even with added strategies in AD-COA-L, such as Bernoulli map-based initialization, ALOBL, and LEO, the time complexity level is still within the same magnitude with other popular algorithms such as PSO, AOA, and WOA.

Additionally, in all algorithms, the space complexity is $\:O\left(ND\right)$, which suggests that the proposed enhancements in AD-COA-L do not consume more of the memory resource than those used by the other compared algorithms and hence is competitive both in time and space efficiency.

This implies that the new AD-COA-L introduces some new strategies for the improvement of exploration and exploitation and retains the same computational complexity as other well-established algorithms. This underlines its efficiency since the improvement in optimization performance does not involve any increase in either time or space complexity. The AD-COA-L therefore provides a well-balanced compromise between performance and computational cost; hence, it should be considered seriously when trying to solve challenging global optimization problems.

**Table 11:** Time and space complexity of AD-COA-L compared to other comparative algorithms.

Algorithm	Time Complexity O	Space Complexity O
PSO ^38^	T · N · D	N · D
AOA ^27^	T · N · D	N · D
WOA ^78^	T · N · D	N · D
SCA ^30^	T · N · D	N · D
SMA ^39^	T · N · D	N · D
WSO ^69^	T · N · D	N · D
SWO ^42^	T · N · D	N · D
INFO ^29^	T · N · D	N · D
GBO ^79^	T · N · D	N · D
CJADE ^70^	T · N · D	N · D
RLTLBO ^71^	T · N · D	N · D
TLABC ^73^	T · N · D	N · D
**AD-COA-L**	T · N · D	N · D

## Application of AD-COA-L to Engineering problems

This section assesses the practical performance of the proposed AD-COA-L by examining its efficacy in solving engineering optimization challenges. The problems encompass tension/compression string design, welded beam design, speed reducer design, tubular column design, piston lever design (PLD), and robot gripper. The research utilizes the static penalty method^80^ to address the limitations in the optimization problem:

39 $$\:\zeta\:\left(z\right)=f\left(z\right)\pm\:\left[\sum\:_{i=1}^{m}\:\:{l}_{i}\cdot\:\text{m}\text{a}\text{x}{\left(0,{t}_{i}\left(z\right)\right)}^{\alpha\:}+\sum\:_{j=1}^{n}\:\:{o}_{j}{\left|{U}_{j}\left(z\right)\right|}^{\beta\:}\right]$$

where the parameter $\:\zeta\:\left(z\right)$ represents the objective function, while $\:{o}_{j}$ and $\:{l}_{j}$ are two positive penalty constants. The functions $\:{U}_{j}\left(z\right)$ and $\:{T}_{i}\left(z\right)$ represent constraint conditions. The parameters $\:\alpha\:$ and $\:\beta\:$ can take on values of either 1 or 2. The resolution of all engineering issues is achieved by employing the parameter configurations specified in Sect. 4.2. The population size, maximum iteration count, and number of independent runs are 50, 500, and 30, respectively.

### Welded beam design

**Fig. 7:**
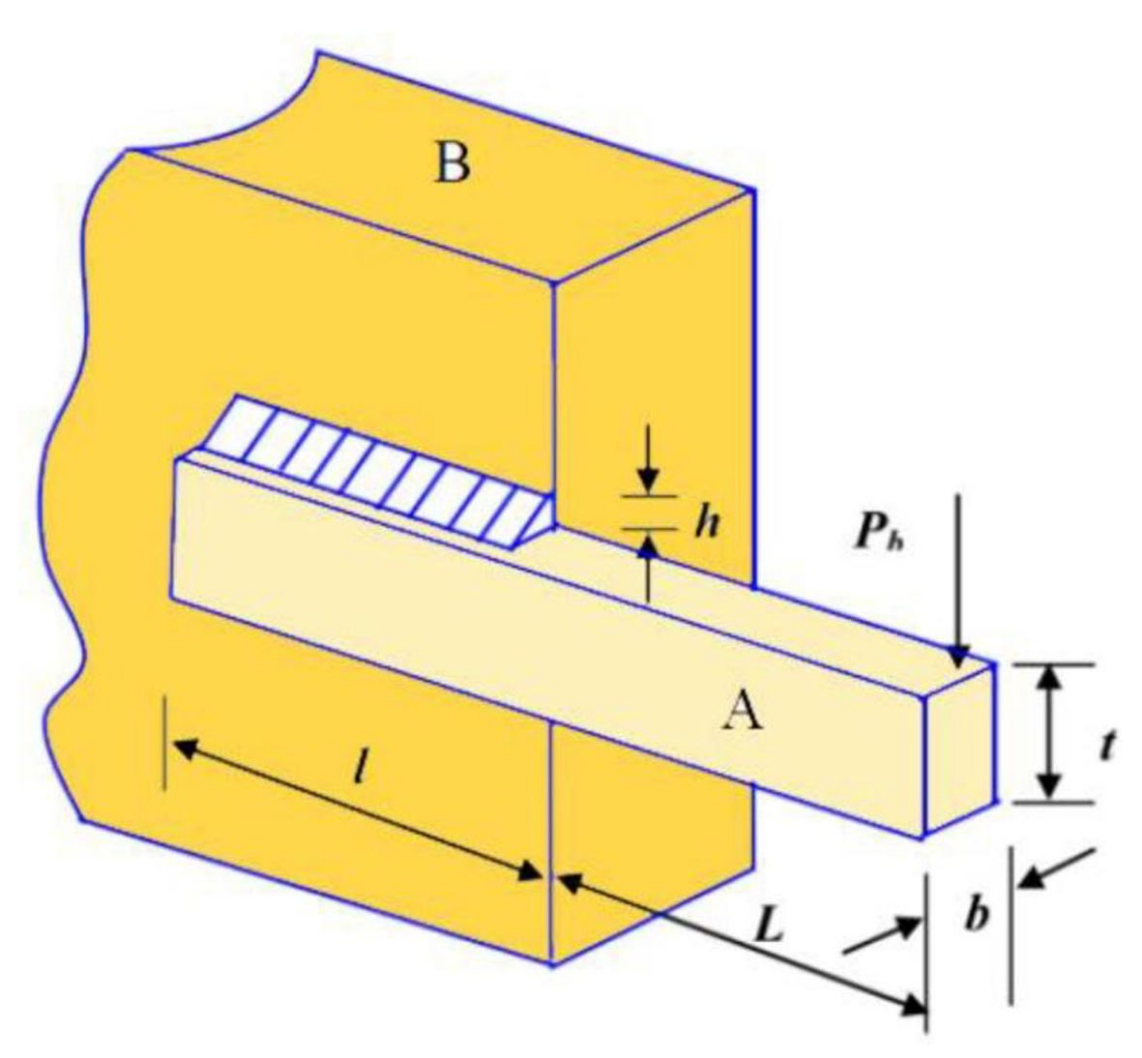
Schematic of Welded beam.

The welded beam structure is a pragmatic design problem frequently employed to assess different optimization techniques. The structure comprises of beam A and the welds that fasten it to member B, as illustrated in Fig. 7. The aim of this design is to determine the most efficient design factors that result in the lowest production costs^81^. The minimization method is constrained by limitations on shear stress (), bending stress in the beam (), buckling load on the bar (-), and the final deflection of the beam (). The optimization process includes four parameters: the length of the clamping bar (), the thickness of the weld (ℎ), the thickness of the bar (), and the height (). The model is presented in the following manner:

Consider

$$\:x\:=\:\left[\:{x}_{1},\:{x}_{2},\:{x}_{3},\:{x}_{4}\right]=\:\left[\:h,\:l,\:t,\:b\:\right]$$

Objective function

$$\:f\left(x\right)=1.10471{x}_{1}^{2}{x}_{2}+0.04811{x}_{3}{x}_{4}(14.0+{x}_{2})$$

Subject to

$$\:{g}_{1}\left(\overrightarrow{x}\right)=\:\tau\:\left(\overrightarrow{x}\right)-{\tau\:}_{max}\le\:0$$

$$\:{g}_{2}\left(\overrightarrow{x}\right)=\:\sigma\:\left(\overrightarrow{x}\right)-{\sigma\:}_{max}\le\:0$$

$$\:{g}_{4}\left(\overrightarrow{x}\right)={x}_{1}-{x}_{4}\le\:0$$

$$\:{g}_{5}\left(\overrightarrow{x}\right)=\:P-{P}_{c}\left(\overrightarrow{x}\right)\le\:0$$

$$\:{g}_{6}\left(\overrightarrow{x}\right)=0.125-{x}_{1}\le\:0$$

$$\:{g}_{1}\left(\overrightarrow{x}\right)=\:\tau\:\left(\overrightarrow{x}\right)-{\tau\:}_{max}\le\:0$$

$$\:{g}_{7}\left(\overrightarrow{x}\right)=1.10471{x}_{1}^{2}+0.04811{x}_{3}{x}_{4}\left(14.0+{x}_{2}\right)-0.5\le\:0$$

Where

$$\:\tau\:\left(\overrightarrow{x}\right)=\sqrt{{\left({\tau\:}^{{\prime\:}}\right)}^{2}+2{\tau\:}^{{\prime\:}}{\tau\:}^{{\prime\:}{\prime\:}}\frac{{x}_{2}}{2R}+\left({\tau\:}^{{\prime\:}{\prime\:}}\right)},{\tau\:}^{{\prime\:}}=\frac{P}{\sqrt{2{x}_{1}{x}_{2}}},{\tau\:}^{{\prime\:}{\prime\:}}=\frac{MR}{J},$$

$$\:M=P(L+\frac{{x}_{2}}{2}),R=\sqrt{\frac{{x}_{2}^{2}}{4}+{\left(\frac{{x}_{1}+{x}_{3}}{2}\right)}^{2}},\sigma\:\left(\overrightarrow{x}\right)=\frac{6PL}{{x}_{4}{x}_{3}^{2}},$$

$$\:J=2\left(\sqrt{2{x}_{1}{x}_{2}}\left[\frac{{x}_{x}^{2}}{4}+{\left(\frac{{x}_{1}+{x}_{3}}{2}\right)}^{2}\right]\right),\delta\:\left(\overrightarrow{x}\right)=\frac{6P{L}^{3}}{E{x}_{4}{x}_{3}^{2}},$$

$$\:{P_c}\left( {\vec x} \right) = \frac{{\sqrt[{4.013E}]{{\frac{{x_3^2x_4^6}}{0}}}}}{{{L^2}}},(1 - \frac{{{x_3}}}{{2{\rm{}}L}}\sqrt {\frac{E}{{4G}}} ),(1 - \frac{{{x_3}}}{{2{\rm{}}L}}\sqrt {\frac{E}{{4G}}} ),$$

Boundaries

$$\:\begin{array}{c}0.1\le\:{x}_{i}\le\:2,i=\text{1,4}\\\:0.1\le\:{x}_{i}\le\:10,i=2.3\end{array}$$

When designing the welded beam, the AD-COA-L method was evaluated with other algorithms such as COA, GWO, HHO, RSA, GJO, jDE, WSO, WOA, PSO, and ASMA. Table 12 presents the minimum cost and the matching optimal variable values obtained by each approach. The welded beam design reached an ideal cost of 1.6702177263 using AD-COA-L.

**Table 12:** Optimization results of different algorithms on welded beam problem.

Algorithm	$\:{\varvec{x}}_{1}$	$\:{\varvec{x}}_{2}$	$\:{\varvec{x}}_{3}$	$\:{\varvec{x}}_{4}$	Optimum Cost
AD-COA-L	0.198685	3.337218	9.191877	0.198685	**1.670071**
COA	0.198756	3.337343	9.190771	0.198752	1.670521
GJO	0.198558	3.342662	9.193462	0.198785	1.671629
RSA	0.180683	3.824357	9.259838	0.208402	1.794052
WOA	0.218367	3.154853	8.667862	0.223452	1.765891
GWO	0.198437	3.342427	9.192508	0.198757	1.671047
HHO	0.199337	3.312611	9.251155	0.198793	1.678433
PSO	0.204328	3.291268	9.015522	0.211175	1.736801
WSO	0.198685	3.337218	9.191877	0.198685	1.670075
jDE	0.198685	3.337218	9.191877	0.198685	1.670079
ASMA	0.363187	2.37327	6.949511	0.361265	2.324504

### Piston lever design (PLD)

**Fig. 8:**
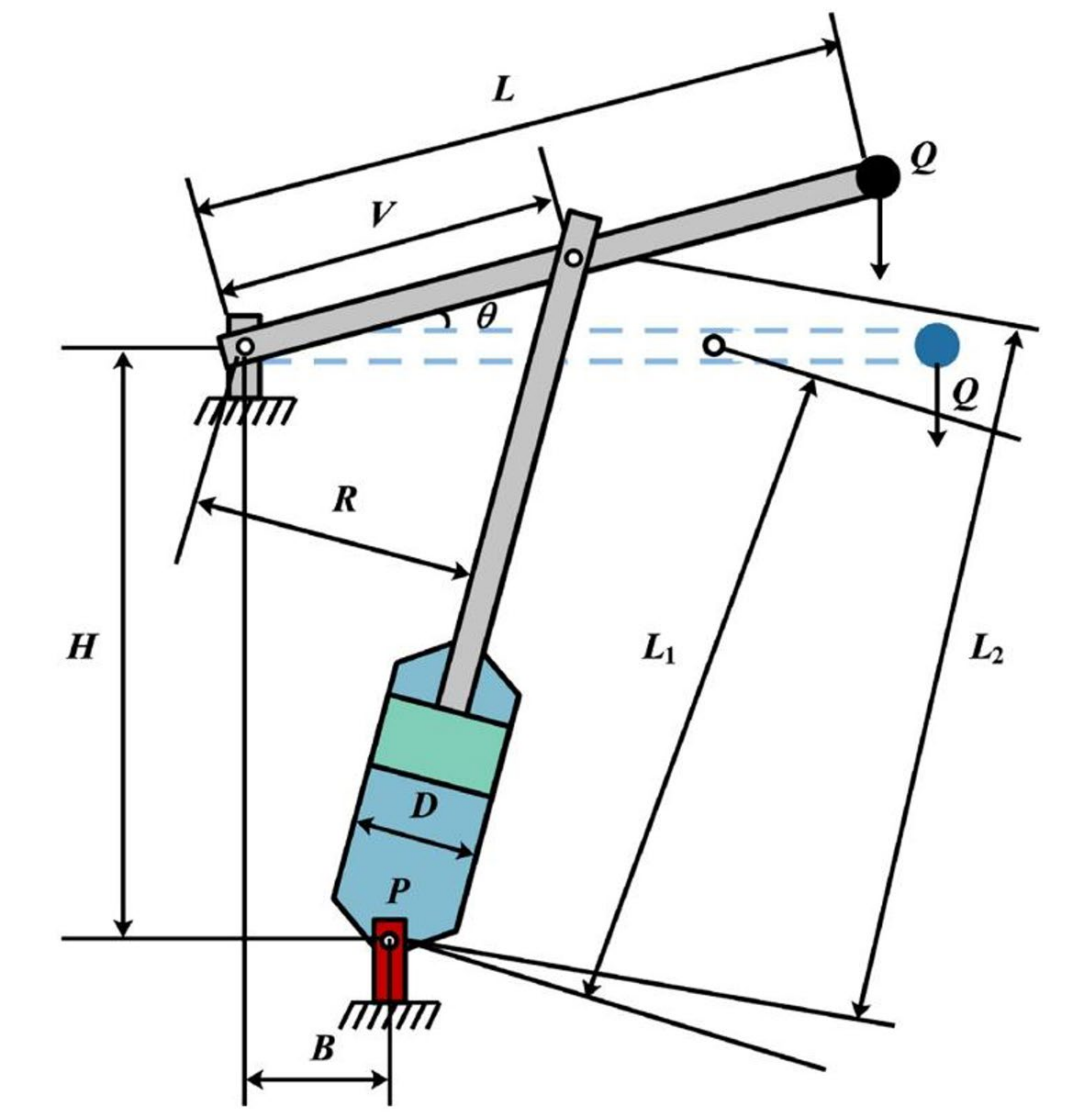
Schematic of Piston lever.

The aim of PLD is to decrease the amount of oil while the piston lever moves from 0° to 45°^82^. The optimization outcomes are influenced by the relative distances $\:H$, $\:B$, $\:D$, and $\:V$ between the piston components. Figure 8 depicts the schematic representation of PLD, and the related mathematical model is defined as follows:

Consider

$$\:x\:=\:\left[\:{x}_{1},\:{x}_{2},\:{x}_{3},\:{x}_{4}\right]=\:\left[\:H,\:B,\:D,\:V\right]$$

Objective function

$$\:f\left(X\right)=\:\left(\frac{1}{4}\right)\pi\:\:{x}_{3}^{2}\left({L}_{2}-\:{L}_{1}\right)$$

Subject to

$$\:{g}_{1\left(X\right)}=\:QL\:cos\left(\theta\:\right)-\:RF\:\le\:\:0,$$

$$\:{g}_{2\left(X\right)}=\:Q\left(L\:-\:{x}_{4}\right)-\:M\:\le\:\:0,$$

$$\:{g}_{3\left(X\right)}=\:1.2\left({L}_{2}-\:{L}_{1}\right)-\:{L}_{1}\le\:\:0,$$

$$\:{g}_{4\left(X\right)}=\:\left(\frac{{x}_{3}}{2}\right)-\:{x}_{2}\le\:\:0,$$

Where

$$\:F=\frac{\pi\:P{x}^{3}2}{4},\:{L}_{1}=\sqrt{{({x}_{4}-{x}_{2})}^{2}+{{x}_{1}}^{2}},$$

$$\:{L}_{2}=\sqrt{{({x}_{4}\text{s}\text{i}\text{n}\theta\:+{x}_{1})}^{2}+{({x}_{2}-{x}_{4}\text{c}\text{o}\text{s}\theta\:)}^{2}}$$

$$\:R=|-{x}_{4}({x}_{4}\text{s}\text{i}\text{n}\theta\:+{x}_{1})+{x}_{1}({x}_{2}-{x}_{4}\text{c}\text{o}\text{s}\theta\:\left)\right|/{L}_{1},\:\theta\:=45^\circ\:,\:Q=\text{10,000}\:\text{l}\text{b}\text{s}$$

$$\:M=1.8\times\:{10}^{6}lbs,P=1500psi,L=240in$$

Boundaries

$$\:0.05\le\:{x}_{1},{x}_{2},{x}_{3}\le\:500$$

$$\:0.05\le\:{x}_{4}\le\:120$$

Table 13 unequivocally shows that the cost of AD-COA-L is significantly lower than that of the comparative approaches. It is important to note that only PSO and SMA fail to accurately identify the optimal design method for PLD. This suggests that while most algorithms have adequate convergence accuracy, they lack the durability seen by AD-COA-L. The AD-COA-L algorithm attains an optimal fitness value of 8.411227.

**Table 13:** Optimization results of different algorithms on Piston Lever Design problem.

Algorithm	$\:{\varvec{x}}_{1}$	$\:{\varvec{x}}_{2}$	$\:{\varvec{x}}_{3}$	$\:{\varvec{x}}_{4}$	Optimum Cost
AD-COA-L	0.05	2.040038	4.081282	120	**8.411227**
COA	0.05	2.040046	4.081286	120	8.411275
GJO	0.05	2.040817	4.082166	119.1476	8.417951
RSA	0.05	2.043883	4.081242	120	8.432057
WOA	0.077454	2.047302	4.090786	119.485	8.739959
GWO	0.049881	2.043668	4.082328	120	8.430411
HHO	0.050445	2.043883	4.081242	120	8.432057
PSO	336.7383	471.7858	2.507971	62.25415	202.355
WSO	0.05	2.040038	4.081282	120	8.411227
jDE	2.782685	336.6265	4.085402	35.41586	166.6067
ASMA	442.2308	500	2.277671	76.54786	174.9272

### Three-bar truss design

The objective of this task is to determine the construction with the lowest weight required for constructing a three-bar truss. This problem consists of two distinct parameters that need to be optimized while considering other restrictions. The mathematical model and the necessary constraint for the parameters are specified as shown:

Consider:

$$\:\overrightarrow{x}=\left[{x}_{1}{x}_{2}\right]$$

Minimize:

$$\:f\left(\overrightarrow{x}\right)=\left(2\sqrt{2}{x}_{1}+{x}_{2}\right)\text{*}l$$

Subject to:

$$\:\begin{array}{l}{g}_{1}\left(\overrightarrow{x}\right)=\frac{\sqrt{2}{x}_{1}+{x}_{2}}{\sqrt{2}{x}_{1}^{2}+2{x}_{1}{x}_{2}}P-\sigma\:\le\:0\\\:{g}_{2}\left(\overrightarrow{x}\right)=\frac{{x}_{2}}{\sqrt{2}{x}_{1}^{2}+2{x}_{1}{x}_{2}}P-\sigma\:\le\:0\\\:{g}_{3}\left(\overrightarrow{x}\right)=\frac{1}{\sqrt{2}{x}_{2}+2{x}_{1}}P-\sigma\:\le\:0\end{array}$$

Where

$$\:\begin{array}{l}l=100\:\text{c}\text{m},P=2\text{K}\text{N}/{\text{c}\text{m}}^{2},\sigma\:=2\text{K}\text{N}/{\text{c}\text{m}}^{2}\\\:0\le\:{x}_{1},{x}_{2}\le\:1\end{array}$$

The comparative outcomes for AD-COA-L and other relevant algorithms for the Three-bar truss problem are displayed in Table 14. Table 14 shows that AD-COA-L achieved the lowest weight when designing the Three-bar truss with optimized 1 and 2. Several other algorithms, such as GWO and HHO had favorable outcomes. However, AD-COA-L surpasses them in performance. The statistical metrics pertaining to this problem are displayed in Table 14. The AD-COA-L algorithm achieved favorable statistical outcomes when compared to all other algorithms.

**Table 14:** Optimized parameters and the best-obtained value for the three-bar truss problem.

	x1	x2	Optimal value
AD-COA-L	0.7873	0.4069	**263.8944**
COA	0.7866	0.4089	263.8956
GJO	0.7757	0.442	264.1285
RSA	0.7875	0.4061	263.8945
WOA	0.7862	0.4098	263.8956
GWO	0.7872	0.4071	263.8945
HHO	0.7873	0.4068	263.8945
PSO	0.782	0.4221	263.9188
WSO	0.8092	0.352	264.5974
jDE	0.7885	0.4035	263.8955
ASMA	0.7831	0.419	263.9249

### Speed reducer design

**Fig. 9:**
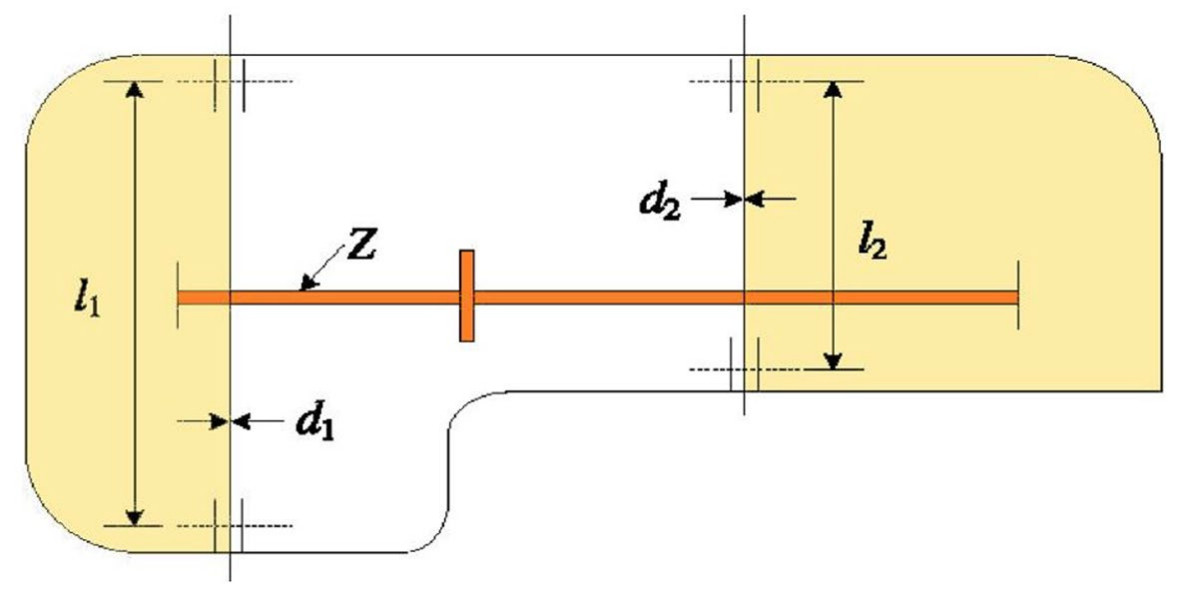
Speed reducer structure.

The design illustrated in Fig. 9 presents a complex optimization problem with the objective of decreasing the weight of the speed reducer^83^. This design issue encompasses 7 variables and is subject to 11 constraints. The variables consist of the teeth module (), face width (), length of the first shaft between bearings (-1), number of teeth on the pinion (), diameter of the first shaft (-1), length of the second shaft between bearings (-2), and diameter of the second shaft (-2). The objective function for this model is specified as follows:

Consider

$$\:x\:=\:\left[\:{x}_{1},\:{x}_{2},\:{x}_{3},\:{x}_{4}{x}_{5}{x}_{6}{x}_{7}\right]=\left[b,\:m,\:z,\:{l}_{1},\:{l}_{2},\:{d}_{1},\:{d}_{2}\right]$$

Objective function

$$\:f\left(x\right)=07854\times\:{x}_{1}\times\:{x}_{2}^{2}\times\:(3.3333\times\:{x}_{3}^{2}+14.9334\times\:{x}_{3}$$

$$\:-43.0934)-1.508\times\:{x}_{1}\times\:({x}_{6}^{2}+{x}_{7}^{2})+7.4777\times\:{x}_{6}^{3}+{x}_{7}^{3}$$

$$\:+0.7854\times\:{x}_{4}\times\:{x}_{6}^{2}+{x}_{5}\times\:{x}_{7}^{2}$$

Subject to

$$\:\begin{array}{ccc}{g}_{1}\left(\overrightarrow{x}\right)&\:=&\:\frac{27}{{x}_{1}\times\:{x}_{2}^{2}\times\:{x}_{3}}-1\le\:0\\\:{g}_{2}\left(\overrightarrow{x}\right)&\:=&\:\frac{397.5}{{x}_{1}\times\:{x}_{2}^{2}\times\:{x}_{3}^{2}}-1\le\:0\\\:{g}_{3}\left(\overrightarrow{x}\right)&\:=&\:\frac{1.93\times\:{x}_{4}^{3}}{{x}_{2}\times\:{x}_{3}\times\:{x}_{6}^{4}}-1\le\:0\\\:{g}_{4}\left(\overrightarrow{x}\right)&\:=&\:\frac{1.93\times\:{x}_{5}^{3}}{{x}_{2}\times\:{x}_{3}\times\:{x}_{7}^{4}}-1\le\:0\end{array}$$

$$\:{g}_{5}\left(\overrightarrow{x}\right)=\frac{1}{110\times\:{x}_{6}^{3}}\times\:\sqrt{{\left(\frac{745\times\:{x}_{4}}{{x}_{2}\times\:{x}_{3}}\right)}^{2}+16.9\times\:{10}^{6}}-1\le\:0$$

$$\:{g}_{6}\left(\overrightarrow{x}\right)=\frac{1}{85\times\:{x}_{7}^{3}}\times\:\sqrt{{\left(\frac{745\times\:{x}_{5}}{{x}_{2}\times\:{x}_{3}}\right)}^{2}+16.9\times\:{10}^{6}}-1\le\:0$$

$$\:{g}_{7}\left(\overrightarrow{x}\right)=\frac{{x}_{2}\times\:{x}_{3}}{40}-1\le\:0$$

$$\:{g}_{8}\left(\overrightarrow{x}\right)=\frac{5\times\:{x}_{2}}{{x}_{1}}-1\le\:0$$

$$\:{g}_{9}\left(\overrightarrow{x}\right)=\frac{{x}_{1}}{12\times\:{x}_{2}}-1\le\:0$$

$$\:{g}_{10}\left(\overrightarrow{x}\right)=\frac{1.5\times\:{x}_{6}+1.9}{{x}_{4}}-1\le\:0$$

$$\:{g}_{11}\left(\overrightarrow{x}\right)=\frac{1.1\times\:{x}_{7}+1.9}{{x}_{5}}-1\le\:0$$

Boundaries

$$\:\begin{array}{c}2.6\le\:{x}_{1}\le\:3.6\\\:0.7\le\:{x}_{2}\le\:0.8\\\:17\le\:{x}_{3}\le\:28\\\:7.3\le\:{x}_{4}\le\:8.3\\\:7.3\le\:{x}_{5}\le\:8.3\\\:2.9\le\:{x}_{6}\le\:3.9\\\:5\le\:{x}_{7}\le\:5.5\end{array}$$

The Speed Reducer problem design involved evaluating the performance of AD-COA-L approach in comparison to various other algorithms, namely COA, GWO, HHO, RSA, GJO, jDE, WSO, WOA, PSO, and ASMA. Table 15 displays the lowest cost and the related ideal variable values achieved by each algorithm. AD-COA-L achieved an optimal cost of 2675.413081664 for the design of the Speed Reducer problem.

**Table 15:** Optimization results of different algorithms on speed reducer problem design.

Algorithm	$\:{\varvec{x}}_{1}$	$\:{\varvec{x}}_{2}$	$\:{\varvec{x}}_{3}$	$\:{\varvec{x}}_{4}$	$\:{\varvec{x}}_{5}$	$\:{\varvec{x}}_{6}$	$\:{\varvec{x}}_{7}$	Optimum Cost	
AD-COA-L	2.606452	0.710209	7.3	7.3	3.381091	5.274581	2674.265		**2.606452**
COA	2.805034	0.7	7.331474	7.3	3.349004	5.286384	2712.064		2.805034
GJO	2.805279	0.7	7.545646	7.32871173	3.355445	5.286641	2716.623		2.805279
RSA	2.805527	0.7	7.3	7.30000003	3.348862	5.28637	2696.12		2.805527
WOA	2.646551	0.7	7.385085	7.39972896	3.348652	5.286488	2721.568		2.646551
GWO	2.793395	0.700026	7.334125	7.42196935	3.348564	5.287437	2715.745		2.793395
HHO	2.751335	0.703378	7.578303	7.3	3.368571	5.288383	2716.935		2.751335
PSO	2.813802	0.7	7.337899	7.3	3.354786	5.297043	2723.614		2.813802
WSO	2.805527	0.7	7.3	7.30000008	3.348862	5.28637	2711.884		2.805527
jDE	2.805527	0.7	7.300145	7.3	3.348862	5.28637	2711.884		2.805527
ASMA	2.735725	0.707382	7.891635	7.57565249	3.808051	5.359336	2933.446		2.735725

### The tension–compression spring design problem

**Fig. 10:**
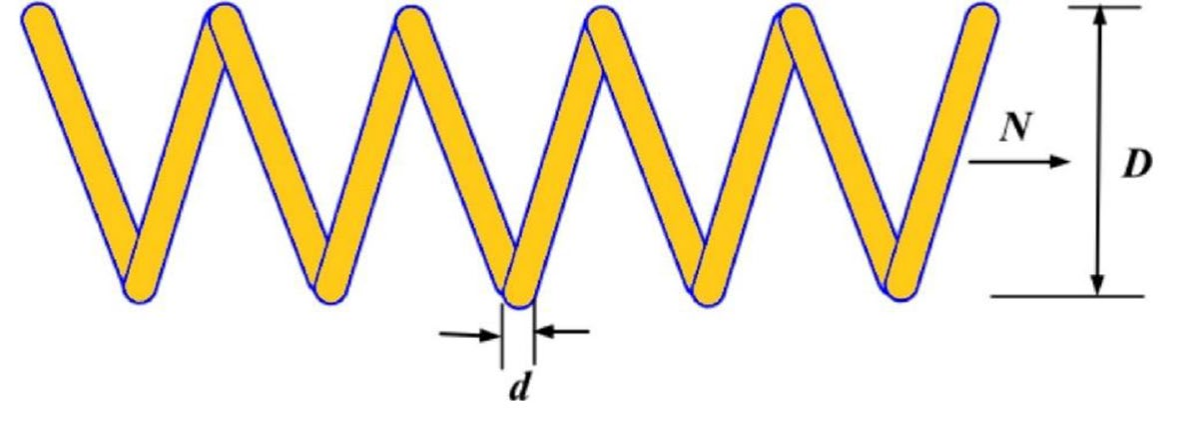
Tension/compressor spring.

The goal of the tension/compression spring design is to minimize the spring’s weight while meeting three specific limitations, as shown in Fig. 10^84^. This optimization involves three key variables: the wire diameter $\:d\left({x}_{1}\right)$, the mean coil diameter ( $\:D\left({x}_{2}\right)$, and the number of active coils $\:N\left({x}_{3}\right)$. These variables need to be optimized as follows:

Consider

$$\:x=\left[{x}_{1}{x}_{2}{x}_{3}\right]=\left[dDN\right]$$

Objective function

$$\:f\left(x\right)=\left({x}_{3}+2\right)\times\:{x}_{2}\times\:{x}_{1}^{2}$$

Subject to

$$\:{g}_{1}\left(x\right)=1-\frac{{x}_{3}\times\:{x}_{2}^{3}}{71785\times\:{x}_{1}^{4}}\le\:0$$

$$\:{g}_{2}\left(x\right)=\frac{4\times\:{x}_{2}^{2}-{x}_{1}\times\:{x}_{2}}{12566\times\:{x}_{1}^{4}}+\frac{1}{5108\times\:{x}_{1}^{2}}-1\le\:0$$

$$\:{g}_{3}\left(x\right)=1-\frac{140.45\times\:{x}_{1}}{{x}_{2}^{2}\times\:{x}_{3}}\le\:0$$

$$\:{g}_{4}\left(x\right)=\frac{{x}_{1}+{x}_{2}}{1.5}-1\le\:0$$

Boundaries

$$\:\begin{array}{ccc}0.05&\:\le\:&\:{x}_{1}\le\:2.0\\\:0.25&\:\le\:&\:{x}_{2}\le\:1.3\\\:2.0&\:\le\:&\:{x}_{3}\le\:15.0\end{array}$$

AD-COA-L was evaluated alongside COA, GWO, HHO, RSA, GJO, jDE, WSO, WOA, PSO, and ASMA algorithms. Table 16 displays the lowest cost and the related optimal variable values attained by each approach. AD-COA-L earned the lowest spring weight of 0.01266352 in the tension/compression spring design task.

**Table 16:** Optimization results of different algorithms on Tension/compression string design.

Algorithm	$\:{\varvec{x}}_{1}$	$\:{\varvec{x}}_{2}$	$\:{\varvec{x}}_{3}$	optimum cost
AD-COA-L	0.051563426	0.353702818	11.467935018	**0.011223223**
COA	0.051812077	0.359650899	11.120926789	0.011225979
GJO	0.051611771	0.354791095	11.462098793	0.011280799
RSA	0.050000000	0.310370833	15.000000000	0.011748750
WOA	0.052195899	0.369033835	10.601967699	0.011228010
GWO	0.051773458	0.358668362	11.183156880	0.011232373
HHO	0.050000000	0.317235532	14.071029568	0.011303744
PSO	0.050000000	0.317341851	14.044153230	0.011286693
WSO	0.051689172	0.356720412	11.288809111	0.011223223
jDE	0.051689034	0.356717092	11.289003739	0.011223511
ASMA	0.050000000	0.310470518	15.000000000	0.011752987

### Tubular column design problem

**Fig. 11:**
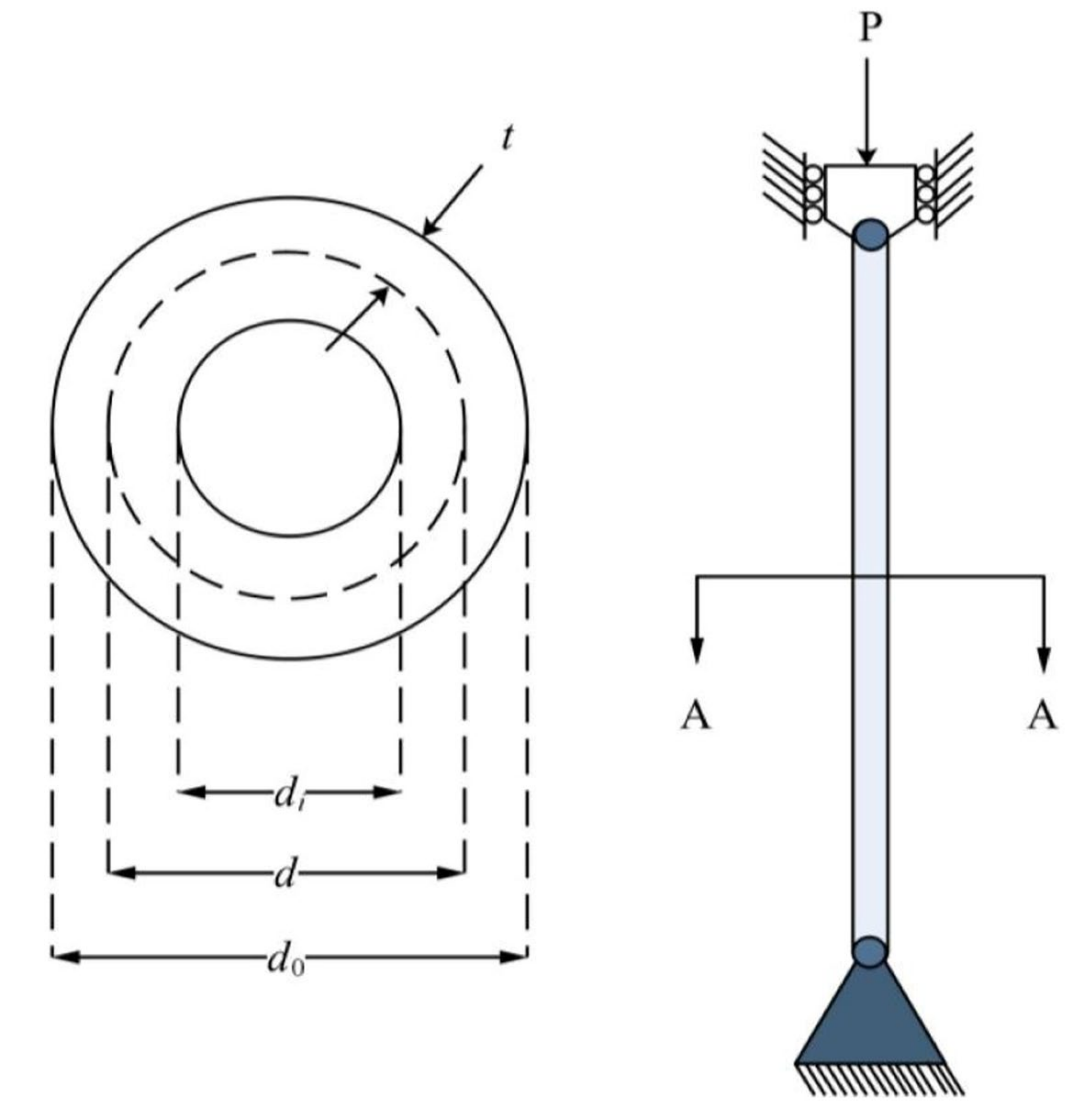
Tubular column design problem.

The challenge of tubular column design is centered around the creation of columns that are uniform in shape and capable of withstanding compression stresses of magnitude $$\:P$$, while simultaneously minimizing cost^85^. The design variables consist of the average diameter $$\:{t}_{1}$$ of the column and the thickness $$\:{t}_{2}$$ of the tube. The column is 250 cm long, has a modulus of elasticity of $$\:0.85\:\times\:\:{10}^{6}$$$$\:kgf/c{m}^{2}$$, and a yield stress of 500 $$\:kgf/c{m}^{2}$$. Figure 11 depicts a homogeneous tubular column structure together with its cross-section. The design model can be characterized as follows:

Consider

$$\:\text{X}=\left[{x}_{1}{x}_{2}\right]$$

Objective function

$$\:f\left(x\right)\:=\:9.8\:x_1\:x_2\:+\:2\:x_1,$$

Subject to

$$\:g_1\left(x\right)\:=\:P\:/\:(\pi\:\:x_1\:x_2\:\sigma\:\_y)\:-\:1\:\le\:\:0,$$

$$\,{g_2}\left( x \right)\, = \,(8\,P\,{L^2})\,/\,(\pi {\,^3}E\,{x_1}\,{x_2}\,({x_1}^2\, + \,{x_2}^2))\, - \,1\, \le \,\,0,$$

$$\:g_3\left(x\right)\:=\:2.0\:/\:x_1\:-\:1\:\le\:\:0,$$

$$\:g_4\left(x\right)\:=\:x_1\:/\:14\:-\:1\:\le\:\:0,$$

$$\:g_5\left(x\right)\:=\:0.2\:/\:x_2\:-\:1\:\le\:\:0,$$

$$\,{g_6}\left( x \right) = {x_2}/8\, - \,1\, \le \,\,0,$$

Boundaries

$$\,2\, \le \,\,{x_1} \le \,\,14,\,0.2\, \le \,\,{x_2} \le \,\,0.8.$$

AD-COA-L has been utilized to resolve problems related to tubular column design. The least cost and corresponding variables produced from the AD-COA-L scheme are compared with those acquired from other algorithms, as shown in Table 17. The data shown in Table 17 demonstrates that the AD-COA-L solution attains the most economical cost. This demonstrates that AD-COA-L has the capability to offer superior quality and more consistent solutions for this challenge, hence highlighting the exceptional performance of AD-COA-L.

**Table 17:** Optimization results of different algorithms on tubular column design problem.

Algorithm	$\:{x}_{1}$	$\:{x}_{2}$	Optimum cost
AD-COA-L	5.450720536	0.290166229	**26.484901272**
COA	5.450721174	0.290166369	26.484911863
GJO	5.451354757	0.290179861	26.488710724
RSA	5.450376472	0.290221043	26.486158443
WOA	5.450520272	0.290209613	26.486246384
GWO	5.450337289	0.290414994	26.496330393
HHO	5.450509448	0.290785382	26.516956690
PSO	5.450720536	0.290166229	26.484901273
WSO	2.069965819	0.458459491	51.701128794
jDE	5.696859619	0.283200952	27.291664238
ASMA	4.631667296	0.800000000	0.000000000

### Robot gripper (RG)

**Fig. 12:**
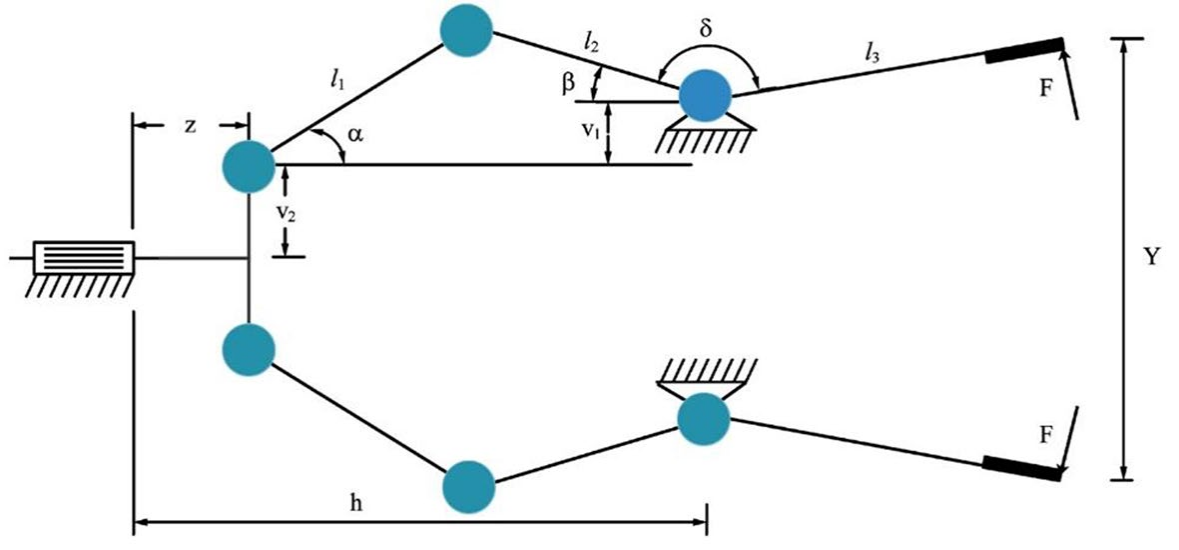
Schematic of Robot Gripper.

RG stands for a complex optimization problem in the field of mechanical structural engineering that deals with restrictions. The primary goal is to reduce the discrepancy between the maximum and minimum forces exerted by a fixture^11^. The optimization outcomes are impacted by six variables: the dimensions of the chain rods, the angular orientation of the chain rods, the vertical and horizontal distances, the clamping pressure, and the placement of the actuator in the robotic gripper. This problem involves seven decision variables: the vertical distance between the first and third link nodes (-1), the lengths of three chain rods (-) for =1,2,3, the horizontal distance between the actuator’s end and the third link node (ℎ), the vertical distance between the first link node and the actuator’s end (-2), and the angle between the second and third chain rods (). The objective function for RG integrates two antagonistic optimization functions. In order to address the issue of prolonged computation times during the iterative process of identifying the optimal value. Figure 12 illustrates the schematic depiction of RG, whereas the actual mathematical model is described as follows:

Consider

$$\:\mathbf{X}=\left[{x}_{1},{x}_{2},{x}_{3},{x}_{4},{x}_{5},{x}_{6},{x}_{7}\right]=\left[{l}_{1},{l}_{2},{l}_{3},{v}_{1},{v}_{2},h,\delta\:\right]$$

Objective function

$$\:\text{M}\text{i}\text{n}\text{i}\text{m}\text{i}\text{z}\text{e}f\left(\mathbf{X}\right)=\underset{z}{max}F(\mathbf{X},z)-\underset{z}{min}F(\mathbf{X},z)$$

Subject to

$$\:{g_1}\left( X \right)\: = \: - {Y_{\min }}\: + \:Y(X,\:{Z_{\min }})\: \le \:\:0,$$

$$\:{g_2}\left( X \right)\: = \: - Y(X,\:{Z_{\min }})\: \le \:\:0,$$

$$\:{g_3}\left( X \right)\: = \:{Y_{\max }}\: - \:Y(X,\:{Z_{\max }})\: \le \:\:0,$$

$$\,{g_4}\left( X \right)\, = \,Y(X,\,{Z_{\max }})\, - \,Y\_G\, \le \,\,0,$$

$$\:{g_5}\left( X \right)\: = \:{x_6}^2\: + \:{x_4}^2 - \:{({x_1}\: + \:{x_2})^2}\: \le \:\:0,$$

$$\,{g_6}\left( X \right)\, = \,x_2^2\, - \,{({x_1}\, - \,{x_4})^2}\, - \,{({x_6}\, - \,{Z_{\max }})^2}\, \le \,\,0,$$

$$\,{g_7}\left( X \right) = \,{Z_{\max }}\, - \,{x_6} \le \,\,0,$$

Where

$$\,\alpha \,\, = \,co{s^{ - 1}}(({x_1}^2\, - \,{x_2}^2\, + \,{g^2})\,/\,(2x1g))\, + \,\phi \,,$$

$$\,\beta \,\, = \,co{s^{ - 1}}(({x_2}^2\, - \,{x_1}^2\, + \,{g^2})\,/\,(2{x_2}g))\, - \,\phi \,,$$

$$\,g\, = \,\surd \,({x_4}^2\, + \,{(z\, - \,{x_6})^2}),$$

$$\,\phi \,\, = \,ta{n^{ - 1}}({x_4}\,/\,({x_6}\, - \,z)),\,Y(X,\,z)\, = \,2({x_5}\, + \,{x_4}\, + \,{x_3}\,sin(\beta \,\, + \,{x_7})),$$

$$\,F(X,\,z)\, = \,(P\,{x_2}\ sin(\alpha \,\, + \,\beta \,))\,/\,(2\,{x_3}cos(\alpha \,)),$$

$$\:{Y}_{min}=50,\:{Y}_{max}=100,\:{Y}_{G}=150,\:{Z}_{max}=100,\:P=100$$

Boundaries

$$\,10\, \le \,\,{x_1},\,{x_2},\,{x_5}\, \le \,\,150$$

$$\:100\:\le\:\:x_3\:\le\:\:200$$

$$\:0\:\le\:\:x_4\:\le\:\:50$$

$$\:100\:\le\:\:x_6\:\le\:\:300$$

$$\,1\, \le \,\,{x_7} \le \,\,3.14$$

Table 18 unambiguously demonstrates that the optimal cost of AD-COA-L is significantly lower than that of the other comparison methods. It is crucial to note that only ASMA did not achieve low optimal values compared to the other algorithms. This suggests that while most algorithms may have enough convergence accuracy, they lack the durability seen by AD-COA-L. The AD-COA-L algorithm achieved an ideal fitness value of 2.525423.

**Table 18:** Optimization results of different algorithms on Robot Gripper problem.

Algorithm	$\:{\varvec{x}}_{1}$	$\:{\varvec{x}}_{2}$	$\:{\varvec{x}}_{3}$	$\:{\varvec{x}}_{4}$	$\:{\varvec{x}}_{5}$	$\:{\varvec{x}}_{6}$	$\:{\varvec{x}}_{7}$	Optimum Cost
AD-COA-L	150	149.305453	199.9876	0.554889	111.5646	100.3744	2.508223	**2.525423**
COA	149.9876	127.240596	194.5136	22.13016	149.9876	121.3282	2.650971	3.220764
GJO	149.9876	149.846886	200	0	149.1175	103.3929	2.365144	2.592627
RSA	149.9876	149.9876	199.9876	0	149.9876	106.9643	2.287727	4.897705
WOA	149.9873	147.639939	158.7813	0	32.03828	151.2824	1.866606	4.163875
GWO	149.3097	149.150576	194.2868	0	84.63068	104.6169	1.987486	2.694334
SSA	149.0688	150	198.9275	0.212219	111.5646	104.7142	2.124441	2.631094
HHO	150	149.826237	197.2751	0	142.3795	105.1166	2.336818	2.659223
PSO	149.9876	97.9795128	186.8842	49.9876	149.9876	135.7171	3.1276	4.037625
SHO	129.61	129.467413	100.6067	0	9.9876	100.5476	1.402619	6.293852
WSO	149.8163	149.137294	200	0.541857	125.9246	101.6464	2.171224	2.558456
jDE	149.9789	149.816348	199.9329	0.032755	148.9041	100.9544	2.279773	2.533071
ASMA	133.9786	105.02752	147.399	14.01085	109.4035	178.4849	2.895224	7.194506

## Conclusion and future directions

This study presents AD-COA-L, an improved version of the crayfish optimization method specifically developed for addressing numerical optimization and real-world engineering issues. During the initialization phase, the Bernoulli map technique is employed to generate a population that is uniformly distributed and of good quality. Subsequently, a dynamic inertia weight is utilized to properly manage the trade-off between exploration and exploitation. Then, the LEO mechanism is employed in subsequent phases to revise specific places, broadening the scope of the search and enhancing the precision of the solution. In order to counteract the original algorithm’s inclination towards local optima, a novel ALOBL technique is introduced to carry out a dimension-by-dimension reversal of the present optimal solution. The efficacy of AD-COA-L is validated through numerical tests using 29 CEC2017 benchmark functions, demonstrating superior convergence rate, solution correctness, stability, and scalability in comparison to other sophisticated algorithms. Furthermore, AD-COA-L demonstrates its competitiveness in seven engineering optimization tasks, highlighting its practical usefulness.

Although the proposed AD-COA-L algorithm introduces accepted performance and advanced strategies, there are some limitations that should be taken into consideration. First, some parameters in AD-COA-L are fixed based on experimental results and keep constant during the optimization process. In spite of these parameters performing well on most test functions, they might not be universally optimal for all types of problems, especially when complex or large-scale optimization tasks are dealt with. Second, for highly diverse problem landscapes, AD-COA-L have some problems for example F26. The fixed parameters of the algorithm prevent it from being globally optimal in an efficient way in some cases. It would cancel this shortcoming by developing appropriate adaptive parameter control mechanisms that change dynamically depending on the problem at hand, which further increases the performance of the algorithm for a larger class of tasks. Third, while ALOBL and LEO were devised to improve exploration with the goal of preventing local optima, AD-COA-L may still experience difficulties converging towards the global optimum with acceptable speed on multimodal problems that exhibit a large number of local optima such as F27. Fourth, in the case of ALOBL, diversity is effective by a selective application to the best solution; it introduces extra computational complexity, enlarging the execution time in some high-dimensional or real-time optimization problems. Finally, the efficiency of the algorithm has been validated on benchmark standard functions mainly, while testing on more complex real-world problems requires further research which will help in evaluating all positive and negative features of the proposed algorithm.

Therefore, future enhancements will prioritize the integration of parallel computing techniques to further enhance the computational expenses while preserving convergence accuracy. In addition, the algorithm’s resilience could be improved by integrating improvements such as the quantum rotation gate and dynamic population development. Based on the encouraging outcomes, AD-COA-L has the potential to be utilized in a wider range of practical optimization tasks, such as feature selection, image segmentation, cloud job scheduling, and PID controller parameter tuning. One potential future goal is to develop a multi-objective version of AD-COA-L that can effectively handle complex multi-objective optimization issues.

## Data Availability

All data generated or analyzed during this study are included directly in the text of this submitted manuscript. There are no additional external files with datasets.
